# Palladium-catalyzed allylic etherification of phenols with vinyl ethylene carbonate

**DOI:** 10.3389/fchem.2022.962355

**Published:** 2022-07-22

**Authors:** Shibo Lin, Xiaotian Zhao, Lihui He, Xuanhao Li, Qian Jiang, Lan Xiang, Yongqin Ye, Xiaohong Gan

**Affiliations:** Department of Pharmacy, Chengdu Second People’s Hospital, Chengdu, China

**Keywords:** palladium-catalyzed, allylic etherification, phenols, vinyl ethylene carbonate, C-O bond formation

## Abstract

The palladium-catalyzed decarboxylative reactions of phenols and vinyl ethylene carbonate to produce allylic aryl ethers under mild conditions have been established. Adopting an inexpensive PdCl_2_(dppf) catalyst promotes the efficient conversion of phenols to the corresponding allylic aryl ethers *via* the formation of a new C-O bond in good isolated yields with complete regioselectivities, acceptable functional group tolerance and operational simplicity. The robust procedure could be completed smoothly by conducting a scaled-up reaction with comparable efficiency to afford the target product.

## Introduction

**FIGURE 1:**
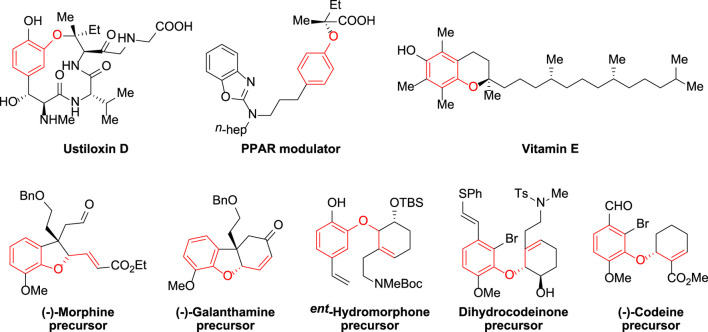
Representative important molecules containing aryl ether fragments.

Aryl ethers are common structural elements found in physiologically important molecules as well as complicated natural substances. (Trost and Toste 1998; Cao et al., 2002; Tanaka et al., 2003; Trost et al., 2003; Sawayama et al., 2004; Trost 2004; Trost et al., 2004; Pochetti et al., 2010). Among the many different varieties, allylic aryl ethers have gotten a lot of interest because of their wide range of uses in academia and industry (Trost and Tang 2002; Parker and Fokas 2006; Varghese and Hudlicky 2014; Zhang et al., 2019a; Zhang et al., 2019b), and selective functionalization of the motif has acted as a foundation for the synthesis of novel bioactive compounds (Figure 1 **)**. The divergent synthesis of allylic aryl ethers from simple substrates, on the other hand, remains a difficult process that has yet to be fully explored. As a result, developing effective synthetic techniques and strategies to produce such therapeutically relevant compounds is of tremendous synthetic and applied importance (Consiglio and Waymouth 1989; Malkov et al., 1999; Trost et al., 2002; López et al., 2003; Mbaye et al., 2003; Ashfeld et al., 2004; Miyabe et al., 2005; Helmchen et al., 2007; Kawatsura et al., 2007; Trost and Brennan 2007; Bandini and Eichholzer 2009).

**FIGURE 2:**
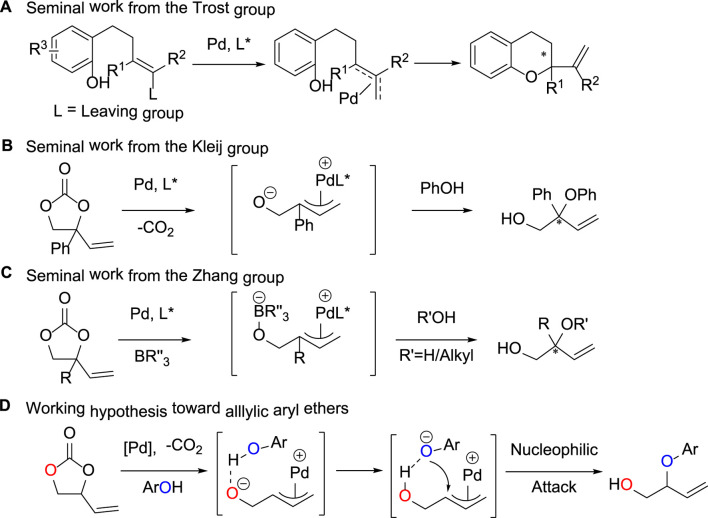
Strategy for Pd-catalyzed decarboxylative reactions of VEC with phenols.

Wandless and Trost, respectively, pioneered Pd-catalyzed allylic alkylation of phenol with π-allyl precursors (Sawayama et al., 2004) and intramolecular cyclization of phenol allyl carbonates (Trost and Toste 1998), which give effective methodologies for the synthesis of allylic C-O bonds. Nonetheless, the response yield is frequently inadequate. Trost (Trost et al., 2003) reported a viable decarboxylative allyl etherification process that employed vinyl epoxides as allylic donors and 4-methoxybenzyl alcohol as an interceptor to produce tertiary-alcohol derivatives in high yields (Figure 2A). For vinyl epoxides, methyl-substituted vinyl epoxides can be readily prepared by isoprene oxidation. Other 2-vinyl oxiranes with various 2-substituents, on the other hand, are difficult to obtain and rather unstable, limiting the reaction substrate’s extension. Recently, vinyl ethylene carbonates (VECs) have been created as adaptable allylic donors with more stable five-membered ring topologies and easier preparation techniques (Khan et al., 2017; Khan et al., 2019). The decarboxylative transformation of vinyl ethylene carbonates catalyzed by transition metals has shown to be a flexible approach for the creation of diverse allylic scaffolds (Shang and Liu 2011; Zhao and Szostak 2019). Kleij’s group reported the palladium-catalyzed decarboxylative allylation of VEC with phenol-based nucleophilic reagents (Xie et al., 2017), which afforded the branched product in high yields in the strong base condition (Figure 2B). It was shown that Cs_2_CO_3_ was critical for the formation of the branched products, and the presence of metal cations induced nucleophilic attack to occur at the central carbon of the palladium intermediate, thus generating the target compounds. This mild protocol is characterized by a fair scope in reaction partners, overall good yields and appreciable enantioinduction. In the same year, Zhang’s group reported the formation of tertiary alcohols or ethers from the reaction of water or alcohol as oxygen donors with VECs (Khan et al., 2017). The experimental process allowed smooth access in high yields, and the mechanism study indicated that the boron reagent played a key part in the reaction (Figure 2C). The process allowed rapid access to valuable tertiary alcohols and ethers in high yields with complete regioselectivities and high enantioselectivities. Work done by the Trost group (Trost and Toste 1999) had established that Pd π-allyl species undergo nucleophilic attack at the secondary carbon in preference to the primary carbon to deliver tertiary centers over secondary ones. This selectivity presumably arises from the greater carbocation-like character of the more highly substituted carbon center following ionization by the Pd (0) metal (Sawayama et al., 2004).

Inspired by the above research results, we envisioned that, in the presence of an appropriate transition-metal catalyst, allylic aryl ethers could be constructed through decarboxylative etherification of VEC with activated phenolic nucleophilic reagents. As illustrated in Figure 2D, we hypothesized that the palladium-catalyzed decarboxylation of VEC could afford reactive amphiphilic π-allyl palladium intermediate, which subsequently forms the desired allylic ethers upon nucleophilic attack by phenols. Herein, we report the palladium-catalyzed conversion of VEC with phenols to construct functionalized allylic aryl ethers in high yields with excellent efficiencies and complete regioselectivities.

## Results and discussion

We commenced our studies by examining the decarboxylative etherification of an easily accessible 1-naphthol **1aa** and VEC **2** as standard reaction partners with using palladium catalysis. The results are summarized in Table 1. Initially, a mixture of **1aa**, **2**, PdCl_2_(dppf) as the catalyst, and Cs_2_CO_3_ as the additive was heated in acetonitrile solvent at 80°C. We were pleased to isolate the desired product, **3aa**, in 72% yield after 15 h (Table 1, entry 1). Subsequently, different palladium sources such as PdCl_2_(dppf)·CH_2_Cl_2_, Pd_2_ (dba)_3_, and PdCl_2_·(bipy) were screened for the reaction under similar conditions, respectively, but most of them obtained no target compound (Table 1, entries 2–4). In addition, no reaction was observed in the absence of palladium catalyst (Table 1, entry 5). With the efficient PdCl_2_(dppf) catalysis in hand, we then tested various additives to evaluate their effects on the reaction outcome. Fortunately, with the help of additives, the reaction efficiency could be promoted to different degrees. Cs_2_CO_3_ achieved the most excellent results in facilitating the reaction, while other additives, including CsOAc, DBU, K_2_CO_3_, and KOH, led to lower isolated yields (Table 1, entries 6–9). Note that the inferior result would be obtained without the help of additives (Table 1, entry 10). Furthermore, solvents such as DMF, DMSO, PhMe, 1,4-dioxane, DCM, DCE, and THF were screened based on the established catalytic system, and MeCN was determined to be the optimal solvent (Table 1, entries 11–17). To our delight, reducing the reaction temperature to 70°C increased the yield of **3aa** from 72 to 81% (Table 1, entry 18). However, turning the temperature down continuously resulted in the remaining unreacted substrates, which was detrimental to the reaction yield (Table 1, entries 19–20). Therefore, the established optimized reaction conditions were **1aa** (0.20 mmol) and **2** (0.30 mmol) as the substrates, PdCl_2_(dppf) (5 mol%) as the catalyst, and Cs_2_CO_3_ as the additive in acetonitrile (2 ml) for 15 h at 70°C.

**TABLE 1:** Optimization of the reaction conditions^a^.

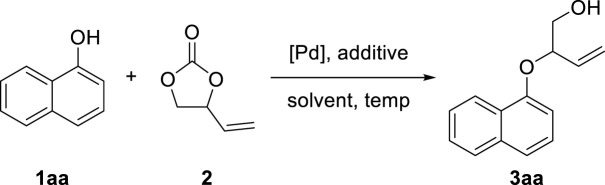					
Entry	Catalyst	Additive	Solvent	Temp (°C)	yield^b^ (%)
1	PdCl_2_(dppf)	Cs_2_CO_3_	MeCN	80	72
2	PdCl_2_(dppf)·CH_2_Cl_2_	Cs_2_CO_3_	MeCN	80	39
3	Pd_2_ (dba)_3_	Cs_2_CO_3_	MeCN	80	0
4	PdCl_2_(bipy)	Cs_2_CO_3_	MeCN	80	0
5	--	Cs_2_CO_3_	MeCN	80	0
6	PdCl_2_(dppf)	CsOAc	MeCN	80	17
7	PdCl_2_(dppf)	DBU	MeCN	80	54
8	PdCl_2_(dppf)	K_2_CO_3_	MeCN	80	14
9	PdCl_2_(dppf)	KOH	MeCN	80	27
10	PdCl_2_(dppf)	--	MeCN	80	0
11	PdCl_2_(dppf)	Cs_2_CO_3_	DMF	80	61
12	PdCl_2_(dppf)	Cs_2_CO_3_	DMSO	80	54
13	PdCl_2_(dppf)	Cs_2_CO_3_	PhMe	80	10
14	PdCl_2_(dppf)	Cs_2_CO_3_	1,4-dioxane	80	13
15	PdCl_2_(dppf)	Cs_2_CO_3_	THF	80	11
16	PdCl_2_(dppf)	Cs_2_CO_3_	DCE	80	9
17	PdCl_2_(dppf)	Cs_2_CO_3_	DCM	80	trace
18	PdCl_2_(dppf)	Cs_2_CO_3_	MeCN	70	81
19	PdCl_2_(dppf)	Cs_2_CO_3_	MeCN	60	72
20	PdCl_2_(dppf)	Cs_2_CO_3_	MeCN	50	37

a Reaction conditions: **1aa** (0.20 mmol), **2** (1.5 equiv, 0.30 mmol), catalyst (5 mol%), additive (0.3 equiv, 0.06 mmol), solvent (2.0 ml), 15 h, open to air.

b Isolated yield of **3aa**.

**FIGURE 3:**
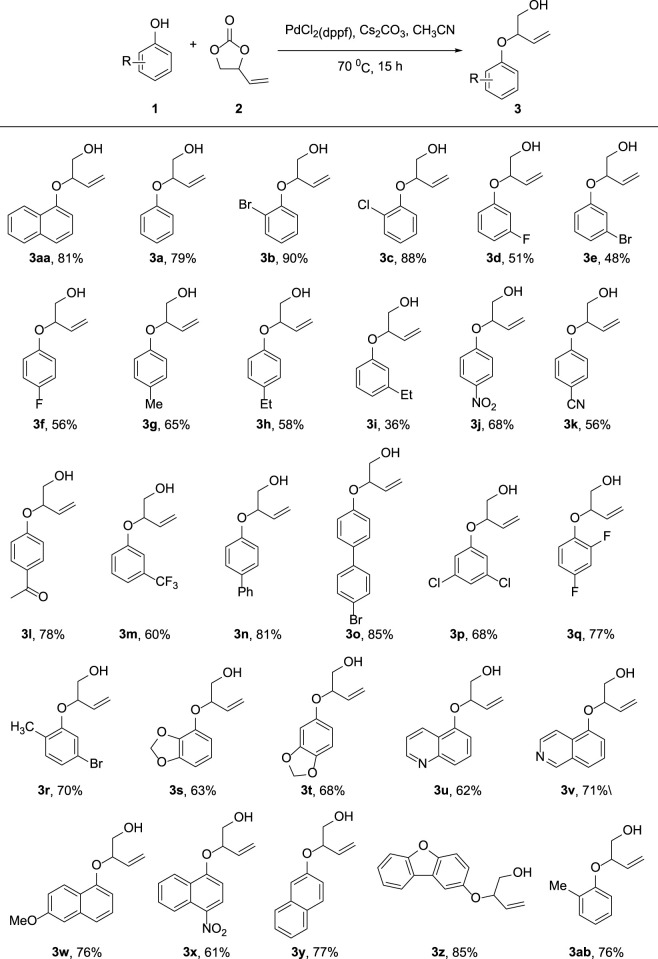
Substrate Scope of phenols. Reaction conditions: Phenol **1** (0.20 mmol), **2** (0.30 mmol), PdCl_2_(dppf) (5 mol%), Cs_2_CO_3_ (0.06 mmol), MeCN (2 ml), 70°C, 15 h. Isolated yields are reported.

As depicted in Figure 3, we investigated the substrate scope of the allylic etherifications under the optimal conditions. The reaction exhibited good functional group tolerance and was a generalized method to construct aryl allyl ether fragments facilely. First, we explored the effect of diverse monosubstituted phenol derivatives as substrates on the reaction. Halogens such as fluoro-, chloro-, and bromo-substituted phenols were well tolerated under identical conditions, affording the corresponding products in good to moderate yield (**3b**-**3f**). Further exploration found that the substitution position of halogen groups on the benzene ring greatly influences the reaction. For instance, halogen substitutions at the *ortho*-position of the phenyl ring (**3b**, **3c**) generated much higher yields than did substitutions at the *para* and *meta*-positions (**3d**-**3f**). Notably, 2-Br substitution on the benzene ring afforded the target compound with an excellent yield of 90% (**3b**). Besides halogen groups, monosubstituted functional groups bearing electron-donating groups (-Me, -Et, -Ph) and electron-withdrawing groups (-CN, -NO_2_, -CF_3_, -COMe) on the phenol ring underwent the reaction smoothly to transform into designed products in moderate yields (**3ab**, **3g**–**3o**). The reaction was also available to achieve phenols with multiple substituents, giving the etherified derivatives satisfyingly (**3p**–**3t**). Unfortunately, pyridyl ethers could not be prepared from hydroxy pyridine through the standard reaction conditions. Subsequently, we were prompted to check the feasibility of allylic etherification of quinolinol and isoquinolinol, which were demonstrated to be appropriate substrates, affording the desired product in 62 and 71% yields, respectively (**3u**, **3v**). In addition, differently substituted naphthol and dibenzofuranol substrates performed high reactivity, and the corresponding naphthol allyl ethers were satisfyingly prepared (**3w**–**3z**). When both *ortho*-positions of the phenol ring are occupied, the reaction will not take place owing to the presence of higher magnitude of steric hindrance.

**FIGURE 4:**
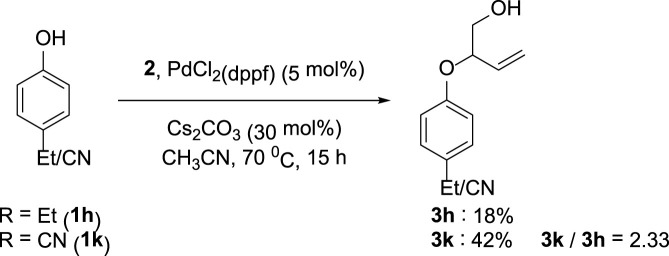
Competition experiments between **1h** and **1k**.

The synthetic utility of the transformation was explored by conducting a gram-scale reaction of 1-naphthol **1aa** and VEC **2** under the ideal condition, which provided the target compound **3aa** with an isolated yield of 52%. Furthermore, competition experiments between differently substituted phenols revealed the preferential conversion of electron-deficient arenes (Figure 4).

## Conclusion

In summary, we have disclosed a general strategy for the synthesis of allylic aryl ethers through palladium-catalyzed O-H activation followed by decarboxylative reactions of aryl phenols and vinyl ethylene carbonate. Under mild conditions, allylic aryl ethers tolerating a broad scope of substitution types and functional groups could be obtained efficiently in good to excellent yield with complete regioselectivities. Notably, a scaled-up reaction could be conducted smoothly *via* this protocol.

## Data Availability

The original contributions presented in the study are included in the article/Supplementary Material, further inquiries can be directed to the corresponding authors.
