# Supplementary material for: Palladium-catalyzed allylic etherification of phenols with vinyl ethylene carbonate
Source: Front Chem. 2022 Jul 22;10:962355. doi: 10.3389/fchem.2022.962355 (PMC9354801; doi:10.3389/fchem.2022.962355)

**Supporting Information**

**Palladium-Catalyzed Allylic Etherification of Arylphenols with Vinyl Ethylene Carbonate**

Shibo Lin, Xiaotian Zhao, Lihui He, Xuanhao Li, Qian Jiang, Lan Xiang, Yongqin Ye*, Xiaohong Gan*

Department of Pharmacy, Chengdu Second People's Hospital, Chengdu 610021, China

**Table of contents**

| S. No. | Section | Page No. |
| --- | --- | --- |
| 1. | General | S2 |
| 2. | General experimental procedure for the reaction | S2 |
| 3. | Spectral data for products | S3-S16 |
| 4. | Scaled-up experiment | S17 |
| 5. | Competition experiment | S17 |
| 6. | Copies of ^1^H, ^13^C, ^19^F NMR and HRMS spectra of products | S18-S76 |

**General**

^1^H and ^13^C spectra were obtained by a Varian 400 MHz or a Bruker 600 MHz NMR spectrometer at 303 K, using CDCl_3_ as the solvent with tetramethylsilane (TMS) as an internal standard at room temperature. High-resolution mass spectra were collected on an Agilent 6224 TOF LC/MS system. Melting points were measured on an SGW X-4 apparatus which was uncorrected. Commercially obtained reagents and solvents were used without further purification. Reactions were monitored by thin layer chromatography (TLC) by using silica gel (GF-254) coated glass slides and detected using UV light (254 nm). Flash chromatography was performed with standard silica gel (200-300 mesh).

**General procedure for the synthesis of aryl allyl ethers**

A mixture of phenol (0.20 mmol), vinyl vthylene carbonate (29 μL, 0.30 mmol), PdCl_2_(dppf) (7 mg, 5 mol %), Cs_2_CO_3_ (20 mg, 0.06 mmol) were added to a 25 mL round bottom flask at room temperature, and then MeCN (2 mL) was added in the reaction system. The mixture was stirred at 70 °C for 15 h. After cooling down to room temperature, the solvent was removed under reduced pressure, and then the residue was directly purified by chromatography on silica gel with petroleum ether/ethyl acetate as the eluent to afford the product.

**Spectral data for products**

**2-(naphthalen-1-yloxy)but-3-en-1-ol** (**3aa**): The crude product was purified via flash chromatography, eluting with petroleum ether/ethyl acetate (10:1) to give a colorless oil (35 mg, 81%).^1^H NMR (400 MHz, CDCl_3_) δ 8.35 – 8.26 (m, 1H), 7.86 – 7.77 (m, 1H), 7.55 – 7.48 (m, 2H), 7.46 (d, *J* = 8.3 Hz, 1H), 7.35 (t, *J* = 7.9 Hz, 1H), 6.88 (d, *J* = 7.6 Hz, 1H), 5.99 – 5.85 (m, 1H), 5.44 (d, *J* = 17.4 Hz, 1H), 5.34 (d, *J* = 10.7 Hz, 1H), 5.00 (d, *J* = 3.6 Hz, 1H), 4.00 – 3.91 (m, 2H). ^13^C NMR (100 MHz, CDCl_3_) δ 152.25, 133.57, 132.90, 126.61, 125.36, 124.93, 124.66, 124.32, 120.70, 119.84, 117.80, 106.27, 79.01, 64.46. ESI-HRMS (*m/z*): calcd for C_14_H_14_O_2_Na [M+Na]^+^:237.0886; found: 237.0895.

**2-phenoxybut-3-en-1-ol** (**3a**): The crude product was purified via flash chromatography, eluting with petroleum ether/ethyl acetate (10:1) to give a colourless oil (26 mg, 79%). ^1^H NMR (600 MHz, CDCl_3_) δ 7.25 (t, *J* = 7.6 Hz, 2H), 6.94 (m, 3H), 5.88 – 5.78 (m, 1H), 5.37 (d, *J* = 17.5 Hz, 1H), 5.30 (d, *J* = 10.6 Hz, 1H), 4.75 (d, *J* = 4.2 Hz, 1H), 3.84 – 3.73 (m, 2H). ^13^C NMR (150 MHz, CDCl_3_) δ 156.88, 133.15, 128.40, 120.30, 117.71, 115.06, 78.76, 64.32. ESI-HRMS (*m/z*): calcd for C_10_H_12_O_2_Na [M+Na]^+^: 187.0730; found: 187.0734.

**2-(2-bromophenoxy)but-3-en-1-ol** (**3b**): The crude product was purified via flash chromatography, eluting with petroleum ether/ethyl acetate (10:1) to give a colourless oil (43 mg, 90%). ^1^H NMR (600 MHz, CDCl_3_) δ 7.53 (dd, *J* = 7.9, 1.6 Hz, 1H), 7.24 – 7.18 (m, 1H), 6.95 (dd, *J* = 8.2, 1.1 Hz, 1H), 6.89 – 6.83 (m, 1H), 5.92 – 5.88 (m, 1H), 5.40 (dd, *J* = 9.9, 8.6 Hz,1H), 5.34 (dd, *J* = 9.8, 1.2 Hz, 1H), 4.77 – 4.71 (m, 1H), 5.40 (dd, *J* = 9.9, 8.6 Hz, 1H), 5.34 (dd, *J* = 9.8, 1.2 Hz, 1H). ^13^C NMR (150 MHz, CDCl_3_) δ 153.39, 132.68, 132.26, 127.37, 121.72, 118.11, 115.17, 112.22, 81.18, 64.32. ESI-HRMS (*m/z*): calcd for C_10_H_11_BrO_2_Na [M+Na]^+^: 264.9835; found: 264.9834.

**2-(2-chlorophenoxy)but-3-en-1-ol** (**3c**): The crude product was purified via flash chromatography, eluting with petroleum ether/ethyl acetate (10:1) to give a colourless oil (35 mg, 88%). ^1^H NMR (600 MHz, CDCl_3_) δ 7.36 (dd, *J* = 8.0, 1.6 Hz, 1H), 7.19 – 7.14 (m, 1H), 6.98 (dd, *J* = 8.3, 1.2 Hz, 1H), 6.92 (td, *J* = 7.6, 1.3 Hz, 1H), 5.91 – 5.86 (m, 1H), 5.43 – 5.37 (m, 1H), 5.37 – 5.31 (m, 1H), 4.78 – 4.70 (m, 1H), 3.86 – 3.76 (m, 2H). ^13^C NMR (150 MHz, CDCl_3_) δ 152.52, 132.68, 129.25, 126.60, 122.83, 121.28, 118.12, 115.49, 81.11, 64.28. ESI-HRMS (*m/z*): calcd for C_10_H_11_ClO_2_Na [M+Na]^+^: 221.0340; found: 221.0339.

**2-(3-fluorophenoxy)but-3-en-1-ol** (**3d**): The crude product was purified via flash chromatography, eluting with petroleum ether/ethyl acetate (5:1) to give a colourless oil (19 mg, 51%). ^1^H NMR (600 MHz, CDCl_3_) δ 7.20 (q, *J* = 7.4 Hz, 1H), 6.71 (d, *J* = 8.3 Hz, 1H), 6.66 (t, *J* = 9.6 Hz, 2H), 5.88 – 5.76 (m, 1H), 5.38 (d, *J* = 17.4 Hz, 1H), 5.34 (d, *J* = 10.7 Hz, 1H), 4.73 (d, *J* = 3.8 Hz, 1H), 3.77 (s, 2H). ^13^C NMR (150 MHz, CDCl_3_) δ 162.45 (d, *J* = 245.0 Hz), 158.19 (d, *J* = 10.7 Hz), 132.57, 129.14 (d, *J* = 10.0 Hz), 118.10, 110.67, 107.08 (d, *J* = 21.4 Hz), 102.71 (d, *J* = 24.8 Hz), 79.12, 64.22. ^19^F NMR (565 MHz, CDCl_3_) δ -111.46. ESI-HRMS (*m/z*): calcd for C_10_H_11_FO_2_Na [M+Na]^+^: 205.0641; found: 205.0634.

**2-(3-bromophenoxy)but-3-en-1-ol** (**3e**): The crude product was purified via flash chromatography, eluting with petroleum ether/ethyl acetate (10:1) to give a colourless oil (23 mg, 48%). ^1^H NMR (600 MHz, CDCl_3_) δ7.14 – 7.10 (m, 1H), 7.10 – 7.08 (m, 2H), 6.88 – 6.85 (m, 1H), 5.87 – 5.77 (m, 1H), 5.40 – 5.33 (m, 2H), 4.75 – 4.72 (m, 1H), 3.78 (d, *J* = 4.1 Hz, 2H). ^13^C NMR (150 MHz, CDCl_3_) δ 157.62, 132.42, 129.49, 123.42, 121.67, 118.42, 118.21, 113.78, 79.12, 64.23. ESI-HRMS (*m/z*): calcd for C_10_H_11_BrO_2_Na [M+Na]^+^: 264.9835; found: 264.9807.

**2-(4-fluorophenoxy)but-3-en-1-ol** (**3f**): The crude product was purified via flash chromatography, eluting with petroleum ether/ethyl acetate (5:1) to give a yellow oil (20 mg, 56%). ^1^H NMR (600 MHz, CDCl_3_) δ 6.94 (t, *J* = 8.6 Hz, 2H), 6.89 – 6.80 (m, 2H), 6.88 – 6.85 (m, 1H), 5.87 – 5.77 (m, 1H), 5.35 (d, *J* = 17.3 Hz, 1H), 5.31 (d, *J* = 10.7 Hz, 1H) 4.66 (d, *J* = 4.7 Hz, 1H), 3.78 (d, *J* = 4.1 Hz, 2H). ^13^C NMR (150 MHz, CDCl_3_) δ 156.56 (d, *J* = 238.9 Hz), 152.95, 132.99, 118.00, 116.37 (d, *J* = 7.8 Hz), 114.77 (d, *J* = 23.1 Hz), 79.85, 64.28. ^19^F NMR (376 MHz, CDCl_3_) δ -123.02 (s, 1F). ESI-HRMS (*m/z*): calcd for C_10_H_11_FO_2_Na [M+Na]^+^: 205.0635; found: 205.0633.

**2-(*p*-tolyloxy)but-3-en-1-ol** (**3g**): The crude product was purified via flash chromatography, eluting with petroleum ether/ethyl acetate (5:1) to give a yellow oil (23 mg, 65%). ^1^H NMR (600 MHz, CDCl_3_) δ 7.06 (d, *J* = 8.5 Hz, 2H), 6.83 (d, *J* = 8.6 Hz, 2H), 5.87 – 5.77 (m, 1H), 5.39 – 5.29 (m, 2H), 4.77 – 4.66 (m, 1H), 3.76 (d, *J* = 6.3 Hz, 2H), 2.28 (s, 3H). ^13^C NMR (150 MHz, CDCl_3_) δ 154.69, 133.25, 129.64, 128.85, 117.71, 114.99, 78.95, 64.35, 19.46. ESI-HRMS (*m/z*): calcd for C_11_H_14_O_2_Na [M+Na]^+^: 201.0886; found: 201.0885.

**2-(4-ethylphenoxy)but-3-en-1-ol** (**3h**): The crude product was purified via flash chromatography, eluting with petroleum ether/ethyl acetate (10:1) to give a colourless oil (22 mg, 58%). ^1^H NMR (600 MHz, CDCl_3_) δ 7.10 (d, *J* = 8.4 Hz, 2H), 6.87 (d, *J* = 8.6 Hz, 2H), 5.90 – 5.80 (m, 1H), 5.42 – 5.29 (m, 2H), 4.73 (dd, *J* = 11.0, 5.7 Hz, 1H), 3.80 – 3.74 (m, 2H), 2.60 (q, *J* = 7.6 Hz, 2H), 1.22 (t, *J* = 7.6 Hz, 3H). ^13^C NMR (150 MHz, CDCl_3_) δ 154.91, 136.11, 133.36, 127.65, 117.59, 114.99, 78.95, 64.34, 26.94, 14.73. ESI-HRMS (*m/z*): calcd for C_12_H_16_O_2_Na [M+Na]^+^: 215.1043; found: 215.1036.

**2-(3-ethylphenoxy)but-3-en-1-ol** (**3i**): The crude product was purified via flash chromatography, eluting with petroleum ether/ethyl acetate (7:1) to give a colourless oil (14 mg, 36%). ^1^H NMR (600 MHz, CDCl_3_) δ 7.17 (t, *J* = 7.6 Hz, 1H), 6.80 (d, *J* = 11.7 Hz, 2H), 6.75 (d, *J* = 8.1 Hz, 1H), 5.92 – 5.77 (m, 1H), 5.39 (d, *J* = 17.4 Hz, 1H), 5.31 (d, *J* = 10.6 Hz, 1H), 4.76 (d, *J* = 4.3 Hz, 1H), 3.77 (s, 2H), 2.61 (q, *J* = 7.3 Hz, 2H), 1.22 (t, *J* = 7.4 Hz, 3H). ^13^C NMR (150 MHz, CDCl_3_) δ 157.96, 145.95, 134.28, 129.20, 120.97, 118.69, 115.78, 112.97, 79.64, 65.39, 28.85, 15.41. ESI-HRMS (*m/z*): calcd for C_12_H_16_O_2_Na [M+Na]^+^: 215.1043; found: 215.1039.

**2-(4-nitrophenoxy)but-3-en-1-ol** (**3j**): The crude product was purified via flash chromatography, eluting with petroleum ether/ethyl acetate (7:1) to give a colourless oil (28 mg, 68%). ^1^H NMR (400 MHz, CDCl_3_) δ 8.18 (d, *J* = 8.8 Hz, 2H), 6.99 (d, *J* = 8.8 Hz, 2H), 5.89 – 5.76 (m, 1H), 5.42 (d, *J* = 6.8 Hz, 1H), 5.38 (s, 1H), 4.88 (d, *J* = 4.9 Hz, 1H), 3.91 – 3.77 (m, 2H). ^13^C NMR (100 MHz, CDCl_3_) δ 161.90, 140.76, 131.68, 124.80, 118.79, 114.72, 79.49, 64.12. ESI-HRMS (*m/z*): calcd for C_10_H_11_NO_4_Na [M+Na]^+^: 232.0580; found: 232.0587.

**4-((1-hydroxybut-3-en-2-yl)oxy)benzonitrile** (**3k**): The crude product was purified via flash chromatography, eluting with petroleum ether/ethyl acetate (3:1) to give a yellow oil (21 mg, 56%). ^1^H NMR (600 MHz, CDCl_3_) δ7.57 – 7.52 (m, 2H), 7.00 – 6.94 (m, 2H), 5.87 – 5.75 (m, 1H), 5.41 – 5.32 (m, 2H), 4.86 – 4.77 (m, 1H), 3.87 – 3.75 (m, 2H). ^13^C NMR (150 MHz, CDCl_3_) δ 160.22, 132.91, 131.86, 118.58, 118.07, 115.46, 103.35, 79.08, 64.09. ESI-HRMS (*m/z*): calcd for C_11_H_12_NO_2_ [M+H]^+^:190.0868; found: 190.0859.

**1-(4-((1-hydroxybut-3-en-2-yl)oxy)phenyl)ethan-1-one** (**3l**): The crude product was purified via flash chromatography, eluting with petroleum ether/ethyl acetate (4:1) to give a colourless oil (32 mg, 78%). ^1^H NMR (600 MHz, CDCl_3_) δ 7.87 (d, *J* = 8.2 Hz, 2H), 6.93 (d, *J* = 8.2 Hz, 2H), 5.86 – 5.75 (m, 1H), 5.34 (dd, *J* = 23.4, 14.2 Hz, 2H), 4.84 (d, *J* = 2.9 Hz, 1H), 3.85 – 3.74 (m, 2H), 2.51 (s, 3H). ^13^C NMR (150 MHz, CDCl_3_) δ 195.99, 160.94, 132.39, 129.58, 129.50, 118.13, 114.49, 78.89, 64.14, 25.29. ESI-HRMS (*m/z*): calcd for C_12_H_15_O_3_ [M+H]^+^: 207.1016; found: 207.1022.

**2-(3-(trifluoromethyl)phenoxy)but-3-en-1-ol** (**3m**): The crude product was purified via flash chromatography, eluting with petroleum ether/ethyl acetate (6:1) to give a colourless oil (27 mg, 60%). ^1^H NMR (600 MHz, CDCl_3_) δ 7.40 – 7.33 (m, 1H), 7.21 (d, *J* = 7.9 Hz, 1H), 7.17 (s, 1H), 7.09 (dd, *J* = 8.2, 2.4 Hz, 1H), 5.87 – 5.78 (m, 1H), 5.42 – 5.33 (m, 1H), 4.79 (td, *J* = 6.1, 1.0 Hz, 1H), 3.83 – 3.79 (m, 2H). ^13^C NMR (150 MHz, CDCl_3_) δ 158.02, 133.37, 129.98, 119.40, 119.26, 117.99, 117.97, 113.07, 113.05, 80.24, 65.26. ESI-HRMS (*m/z*): calcd for C_11_H_11_F_3_O_2_Na [M+Na]^+^: 255.0603; found: 255.0597.

**2-([1,1'-biphenyl]-4-yloxy)but-3-en-1-ol** (**3n**): The crude product was purified via flash chromatography, eluting with petroleum ether/ethyl acetate (4:1) to give a white solid (39 mg, 81%). mp 78.6-80.1 ºC. ^1^H NMR (600 MHz, CDCl_3_) δ 7.55 (d, *J* = 7.3 Hz, 2H), 7.51 (d, *J* = 8.3 Hz, 2H), 7.42 (t, *J* = 7.4 Hz, 2H), 7.32 (t, *J* = 7.1 Hz, 1H), 7.02 (d, *J* = 8.3 Hz, 2H), 5.94 – 5.78 (m, 1H), 5.43 (d, *J* = 17.3 Hz, 1H), 5.36 (d, *J* = 10.5 Hz, 1H), 4.82 (d, *J* = 4.3 Hz, 1H), 3.91 – 3.75 (m, 2H). ^13^C NMR (150 MHz, CDCl_3_) δ 156.43, 139.67, 133.41, 133.06, 127.69, 127.09, 125.73, 117.87, 115.28, 78.90, 64.35. ESI-HRMS (*m/z*): calcd for C_16_H_16_O_2_Na [M+Na]^+^: 263.1043; found: 263.1047.

**2-((4'-bromo-[1,1'-biphenyl]-4-yl)oxy)but-3-en-1-ol** (**3o**): The crude product was purified via flash chromatography, eluting with petroleum ether/ethyl acetate (5:1) to give a white solid (54 mg, 85%). mp 114.8-116.6 ºC. ^1^H NMR (600 MHz, CDCl_3_) δ 7.54 – 7.51 (m, 2H), 7.47 – 7.44 (m, 2H), 7.41 – 7.38 (m, 2H), 7.02 – 6.99 (m, 2H), 5.93 – 5.81 (m, 1H), 5.44 – 5.39 (m, 1H), 5.38 – 5.33 (m, 1H), 4.81 (dd, *J* = 11.0, 6.0 Hz, 1H), 3.85 – 3.77 (m, 2H). ^13^C NMR (150 MHz, CDCl_3_) δ 156.70, 138.60, 132.95, 132.13, 130.79, 127.30, 126.94, 119.90, 117.97, 115.40, 78.92, 64.34. ESI-HRMS (*m/z*): calcd for C_16_H_16_BrO_2_ [M+H]^+^: 319.0394; found: 319.0400.

**2-(3,5-dichlorophenoxy)but-3-en-1-ol** (**3p**): The crude product was purified via flash chromatography, eluting with petroleum ether/ethyl acetate (8:1) to give a colourless oil (31 mg, 68%). ^1^H NMR (600 MHz, CDCl_3_) δ 6.96 (t, *J* = 1.7 Hz, 1H), 6.82 (d, *J* = 2.0 Hz, 2H), 5.85 – 5.73 (m, 1H), 5.41 – 5.32 (m, 2H), 4.72 (dt, *J* = 5.7, 4.9 Hz, 1H), 3.80 – 3.75 (m, 2H). ^13^C NMR (150 MHz, CDCl_3_) δ 157.84, 134.28, 131.83, 120.63, 118.61, 114.00, 79.44, 64.09. ESI-HRMS (*m/z*): calcd for C_10_H_9_Cl_2_O_2_ [M-H]^-^:230.9985; found: 230.9992.

**2-(2,4-difluorophenoxy)but-3-en-1-ol** (**3q**): The crude product was purified via flash chromatography, eluting with petroleum ether/ethyl acetate (5:1) to give a colourless oil (30 mg, 77%). ^1^H NMR (400 MHz, CDCl_3_) δ 7.03 – 6.90 (m, 1H), 6.88 – 6.81 (m, 1H), 6.77 – 6.73 (m, 1H), 5.88 – 5.80 (m, 1H), , 5.33 (t, *J* = 13.6 Hz, 2H), 4.62 (d, *J* = 3.3 Hz, 1H), 3.87 – 3.72 (m, 2H). ^13^C NMR (100 MHz, CDCl_3_) δ 156.20 (dd, *J* = 243.1, 10.6 Hz), 152.44 (dd, *J* = 248.7, 12.1 Hz), 141.09 (dd, *J* = 10.8, 3.6 Hz), 132.54, 118.71, 118.47 (dd, *J* = 9.5, 2.4 Hz), 109.53 (dd, *J* = 22.5, 3.9 Hz), 103.86 (dd, *J* = 26.7, 22.6 Hz), 82.46, 64.18. ^19^F NMR (565 MHz, CDCl_3_) δ -118.17, -128.04. ESI-HRMS (*m/z*): calcd for C_10_H_10_F_2_O_2_Na [M+Na]^+^: 223.0541; found: 223.0550.

**2-(5-bromo-2-methylphenoxy)but-3-en-1-ol** (**3r**): The crude product was purified via flash chromatography, eluting with petroleum ether/ethyl acetate (10:1) to give a colourless oil (36 mg, 70%). ^1^H NMR (600 MHz, CDCl_3_) δ 6.99 (s, 2H), 6.97 (s, 1H), 5.88 – 5.74 (m, 1H), 5.34 (dd, *J* = 15.3, 14.3 Hz, 2H), 4.74 – 4.74 (m, 1H), 3.82 – 3.76 (m, 2H), 2.19 (s, 3H). ^13^C NMR (150 MHz, CDCl_3_) δ 155.47, 132.64, 130.78, 125.32, 122.91, 118.27, 118.01, 115.88, 79.22, 64.21, 15.02. ESI-HRMS (*m/z*): calcd for C_11_H_13_BrO_2_Na [M+Na]^+^: 280.9972; found: 280.9971.

**2-(benzo[d][1,3]dioxol-4-yloxy)but-3-en-1-ol** (**3s**): The crude product was purified via flash chromatography, eluting with petroleum ether/ethyl acetate (5:1) to give a colourless oil (26 mg, 63%). ^1^H NMR (600 MHz, CDCl_3_) δ 6.72 (t, *J* = 8.2 Hz, 1H), 6.55 – 6.53 (m, 2H), 5.93 (s, 1H), 5.91 – 5.80 (m, 1H), 5.39 (d, *J* = 17.3 Hz, 1H), 5.29 (d, *J* = 10.8 Hz, 1H), 4.82 (d, *J* = 3.2 Hz, 1H), 3.83 – 3.71 (m, 2H). ^13^C NMR (150 MHz, CDCl_3_) δ 147.93, 140.95, 135.25, 132.97, 120.93, 117.97, 110.82, 102.14, 100.05, 80.93, 64.17. ESI-HRMS (*m/z*): calcd for C_11_H_12_O_4_Na [M+Na]^+^: 231.0628; found: 231.0632.

**2-(benzo[d][1,3]dioxol-5-yloxy)but-3-en-1-ol** (**3t**): The crude product was purified via flash chromatography, eluting with petroleum ether/ethyl acetate (5:1) to give a colourless oil (28 mg, 68%). ^1^H NMR (600 MHz, CDCl_3_) δ 6.68 (d, *J* = 8.3 Hz, 1H), 6.53 (s, 1H), 6.38 (d, *J* = 8.3 Hz, 1H), 5.91 (s, 2H), 5.86 – 5.75 (m, 1H), 5.35 (d, *J* = 17.3 Hz, 1H), 5.31 (d, *J* = 10.7 Hz, 1H), 4.60 (d, *J* = 5.2 Hz, 1H), 3.78 – 3.69 (m, 2H). ^13^C NMR (150 MHz, CDCl_3_) δ 152.20, 147.13, 141.14, 133.23, 117.89, 107.22, 106.93, 100.19, 98.63, 80.31, 64.29. ESI-HRMS (*m/z*): calcd for C_11_H_12_O_4_Na [M+Na]^+^: 231.0628; found: 231.0630.

**2-(quinolin-5-yloxy)but-3-en-1-ol** (**3u**): The crude product was purified via flash chromatography, eluting with petroleum ether/ethyl acetate (2:1) to give a gray solid (26 mg, 62%). mp 125.1-127.8 ºC. ^1^H NMR (600 MHz, CDCl_3_) δ 8.88 – 8.85 (m, 1H), 8.57 (d, *J* = 8.3 Hz, 1H), 7.68 (d, *J* = 8.6 Hz, 1H), 7.54 (t, *J* = 8.2 Hz, 1H), 7.33 (ddd, *J* = 8.3, 4.1, 1.6 Hz, 1H), 6.89 (d, *J* = 7.7 Hz, 1H), 5.93 – 5.87 (m, 1H), 5.42 (d, *J* = 17.7 Hz, 1H), 5.35 (d, *J* = 11.0 Hz, 1H), 5.00 (dd, *J* = 10.0, 6.5 Hz, 1H), 3.98 (dd, *J* = 11.9, 7.2 Hz, 1H), 3.91 (dd, *J* = 11.9, 3.7 Hz, 1H). ^13^C NMR (150 MHz, CDCl_3_) δ 152.02, 149.52, 147.94, 132.60, 129.71, 128.24, 120.92, 120.20, 119.22, 118.07, 106.63, 79.44, 64.28. ESI-HRMS (*m/z*): calcd for C_13_H_14_NO_2_ [M+H]^+^: 216.1019; found: 216.1024.

**2-(isoquinolin-5-yloxy)but-3-en-1-ol** (**3v**): The crude product was purified via flash chromatography, eluting with petroleum ether/ethyl acetate (2:1) to give a gray oil(26 mg, 62%). ^1^H NMR (400 MHz, CDCl_3_) δ 9.17 (s, 1H), 8.46 (s, 1H), 8.00 (s, 1H), 7.53 (d, *J* = 7.5 Hz, 1H), 7.46 (t, *J* = 7.8 Hz, 1H), 7.06 (d, *J* = 7.6 Hz, 1H), 6.01 – 5.80 (m, 1H), 5.42 (d, *J* = 17.4 Hz, 1H), 5.35 (d, *J* = 10.7 Hz, 1H), 5.01 (s, 1H), 3.99 (dd, *J* = 11.7, 7.3 Hz, 1H), 3.91 (dd, *J* = 11.7, 2.8 Hz, 1H). ^13^C NMR (100 MHz, CDCl_3_) δ 151.43, 150.70, 141.21, 132.46, 128.48, 127.83, 126.32, 118.95, 118.12, 114.01, 110.18, 79.47, 64.30. ESI-HRMS (*m/z*): calcd for C_13_H_14_NO_2_ [M+H]^+^: 216.1019; found: 216.1021.

**2-((6-methoxynaphthalen-1-yl)oxy)but-3-en-1-ol** (**3w**): The crude product was purified via flash chromatography, eluting with petroleum ether/ethyl acetate (10:1) to give a red oil (37 mg, 76%). ^1^H NMR (600 MHz, CDCl_3_) δ 8.20 (d, *J* = 9.2 Hz, 1H), 7.36 – 7.28 (m, 2H), 7.14 (dd, *J* = 9.1, 2.6 Hz, 1H), 7.11 (d, *J* = 2.5 Hz, 1H), 6.74 (d, *J* = 7.4 Hz, 1H), 5.96 – 5.86 (m, 1H), 5.43 (dt, *J* = 17.3, 1.2 Hz, 1H), 5.33 (dd, *J* = 11.5, 1.1 Hz, 1H), 5.00 – 4.95 (m, 1H), 3.97 – 3.93 (m, 1H), 3.92 (s, 3H), 3.90 – 3.85 (m, 1H). ^13^C NMR (150 MHz, CDCl_3_) δ 157.10, 152.53, 135.03, 133.02, 125.46, 122.47, 120.19, 118.84, 117.73, 116.79, 104.79, 104.47, 78.97, 64.46, 54.26, 28.68. ESI-HRMS (*m/z*): calcd for C_15_H_16_O_3_Na [M+Na]^+^: 267.0992; found: 267.0994.

**2-((4-nitronaphthalen-1-yl)oxy)but-3-en-1-ol** (**3x**): The crude product was purified via flash chromatography, eluting with petroleum ether/ethyl acetate (5:1) to give a yellow solid (31 mg, 61%). mp 74.6-76.8 ºC.  ^1^H NMR (400 MHz, CDCl_3_) δ 8.77 (d, *J* = 8.7 Hz, 1H), 8.43 (d, *J* = 8.4 Hz, 1H), 8.34 (d, *J* = 8.6 Hz, 1H), 7.75 (t, *J* = 7.8 Hz, 1H), 7.62 (t, *J* = 7.5 Hz, 1H), 6.86 (d, *J* = 8.7 Hz, 1H), 6.01 – 5.82 (m, 1H), 5.54 – 5.34 (m, 2H), 5.11 (s, 1H), 4.10 – 3.99 (m, 1H), 3.96 – 3.93 (m, 1H). ^13^C NMR (100 MHz, CDCl_3_) δ 157.50, 138.46, 131.60, 129.06, 125.96, 125.75, 125.68, 124.90, 122.60, 121.50, 118.75, 103.86, 79.85, 64.20. ESI-HRMS (*m/z*): calcd for C_14_H_13_NO_4_Na [M+Na]^+^: 282.0737; found: 282.0739.

**2-(naphthalen-2-yloxy)but-3-en-1-ol** (**3y**): The crude product was purified via flash chromatography, eluting with petroleum ether/ethyl acetate (10:1) to give a yellow oil (33 mg, 77%). ^1^H NMR (600 MHz, CDCl_3_) δ 7.75 (t, *J* = 8.8 Hz, 2H), 7.69 (d, *J* = 8.2 Hz, 1H), 7.42 (ddd, *J* = 8.2, 7.0, 1.1 Hz, 1H), 7.33 (ddd, *J* = 8.1, 6.9, 1.2 Hz, 1H), 7.21 – 7.17 (m, 2H), 5.97 – 5.81 (m, 1H), 5.43 (dt, *J* = 17.3, 1.2 Hz, 1H), 5.37 – 5.28 (m, 1H), 4.91 (dd, *J* = 11.0, 6.1 Hz, 1H), 3.87 – 3.80 (m, 2H). ^13^C NMR (150 MHz, CDCl_3_) δ 154.63, 133.32, 132.96, 128.45, 128.17, 126.59, 125.82, 125.37, 122.88, 118.13, 117.86, 108.44, 78.80, 64.35. ESI-HRMS (*m/z*): calcd for C_14_H_14_O_2_Na [M+Na]^+^: 237.0886; found: 237.0888.

**2-(dibenzo[*b*,*d*]furan-2-yloxy)but-3-en-1-ol** (**3z**): The crude product was purified via flash chromatography, eluting with petroleum ether/ethyl acetate (10:1) to give a yellow solid (43 mg, 85%). mp 50.4-52.7 ºC. ^1^H NMR (400 MHz, CDCl_3_) δ 7.89 (d, *J* = 7.7 Hz, 1H), 7.54 (d, *J* = 8.2 Hz, 1H), 7.45 (dd, *J* = 14.3, 6.1 Hz, 3H), 7.32 (t, *J* = 7.4 Hz, 1H), 7.09 (d, *J* = 8.9 Hz, 1H), 5.98 – 5.83 (m, 1H), 5.43 (d, *J* = 17.4 Hz, 1H), 5.35 (d, *J* = 10.7 Hz, 1H), 4.82 (q, *J* = 5.3 Hz, 1H), 3.84 (d, *J* = 5.8 Hz, 2H). ^13^C NMR (100 MHz, CDCl_3_) δ 155.90, 152.93, 150.28, 133.16, 126.20, 123.72, 123.28, 121.44, 119.58, 118.03, 116.02, 111.00, 110.71, 106.44, 80.39, 64.39. ESI-HRMS (*m/z*): calcd for C_16_H_14_O_3_Na [M+Na]^+^: 277.0835; found: 277.0838.

**2-(o-tolyloxy)but-3-en-1-ol** (**3ab**): The crude product was purified via flash chromatography, eluting with petroleum ether/ethyl acetate (7:1) to give a yellow solid (27 mg, 76%). ^1^H NMR (600 MHz, CDCl_3_) δ 7.08 (s, 1H), 7.06 (s, 1H), 6.85 (s, 1H), 6.83 (s, 1H), 5.83 (ddd, *J* = 17.4, 10.7, 6.0 Hz, 1H), 5.37 (dt, *J* = 17.4, 1.3 Hz, 1H), 5.31 (dt, *J* = 10.7, 1.2 Hz, 1H), 4.75 – 4.69 (m, 1H), 3.77 (d, *J* = 2.9 Hz, 1H), 3.76 (d, *J* = 1.0 Hz, 1H), 2.29 (s, 3H). ^13^C NMR (150 MHz, CDCl_3_) δ 154.70, 133.27, 129.63, 128.85, 117.68, 115.01, 78.96, 64.33, 19.45. ESI-HRMS (*m/z*): calcd for C_11_H_14_O_2_Na [M+Na]^+^: 201.0881; found: 201.0876.

**Scaled-up experiment**

A suspension of 1-naphthol (**1aa**) (1.0 g, 6.94 mmol), vinyl ethylene carbonate (**2**) (1.18 g, 10.41 mmol), PdCl_2_·(dppf) (253 mg, 5 mol %), and Cs_2_CO_3_ (676 mg, 2.0 mmol) in MeCN (50 mL) was heated at 70 °C for 15 h. The solvent was removed under reduced pressure and the residue was directly purified by flash column chromatography (petroleum ether/ethyl acetate=10:1) to give the title compound **3aa** (772 mg, 52 %).

**Competition experiment**

A suspension of 4-ethylphenol (**1h**) (24 mg, 0.2 mmol), 4-hydroxybenzonitrile (**1k**) (23 mg, 0.2 mmol), vinyl ethylene carbonate (**2**) (22 mg, 0.2 mmol), PdCl_2_(dppf) (7 mg, 5 mol %), and Cs_2_CO_3_ (20 mg, 0.06 mmol) in MeCN (2 mL) was heated at 70 °C for 15 h. The solvent was removed under reduced pressure and the residue was directly purified by flash column chromatography (petroleum ether/ethyl acetate=10:1 to petroleum ether/ethyl acetate=3:1) to yield compound **3h** (7 mg, 18 %) and **3k** (16 mg, 42 %), respectively.

^1^H, ^13^C NMR and HRMS Spectra of Compound **3aa**


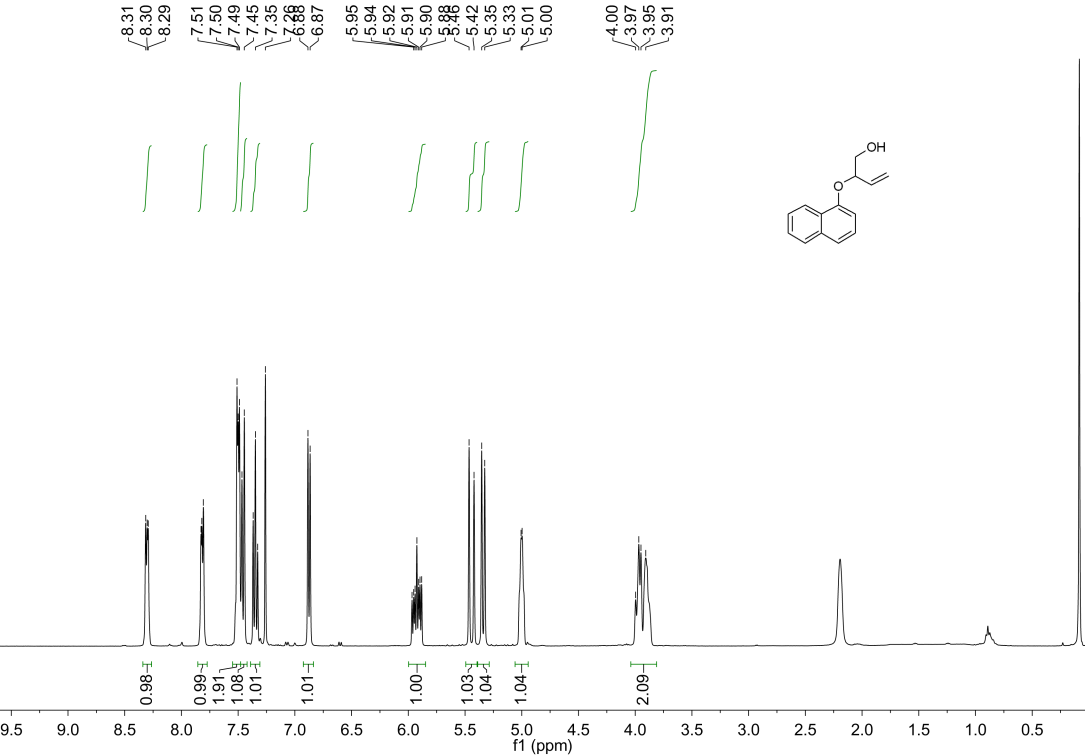


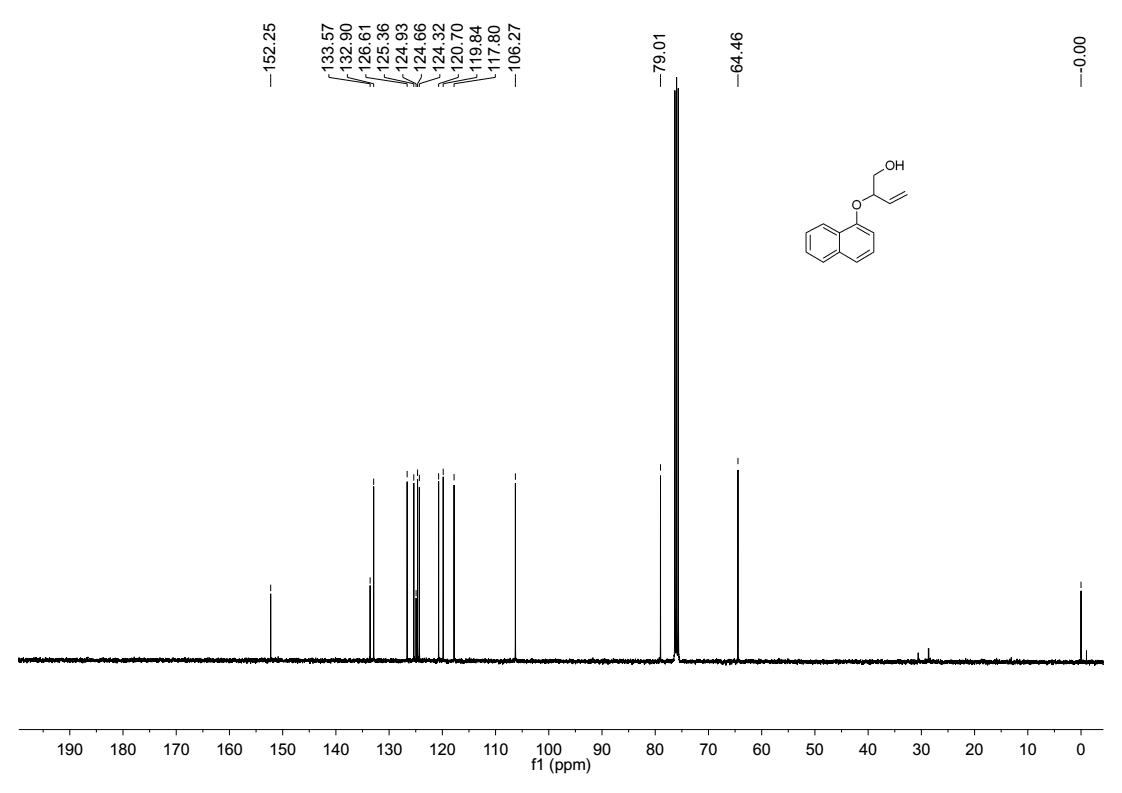


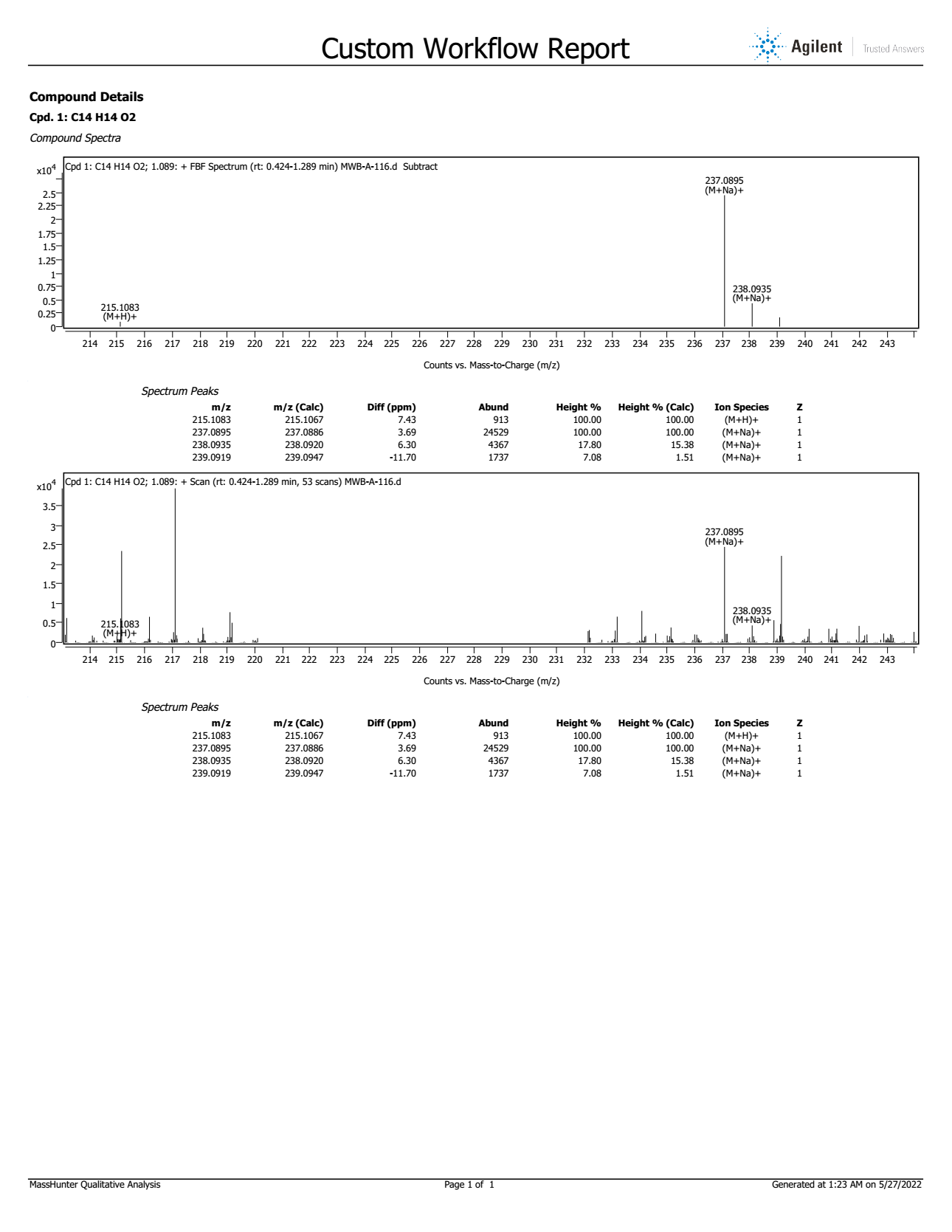


^1^H, ^13^C NMR and HRMS Spectra of Compound **3a**

**
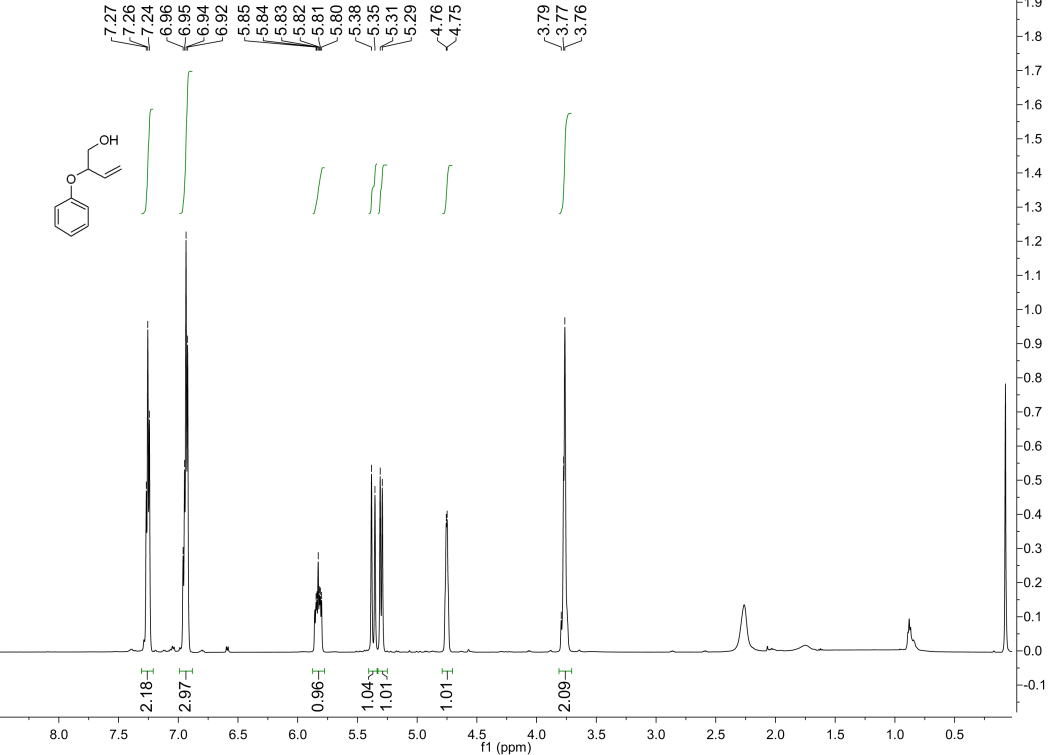
**

**
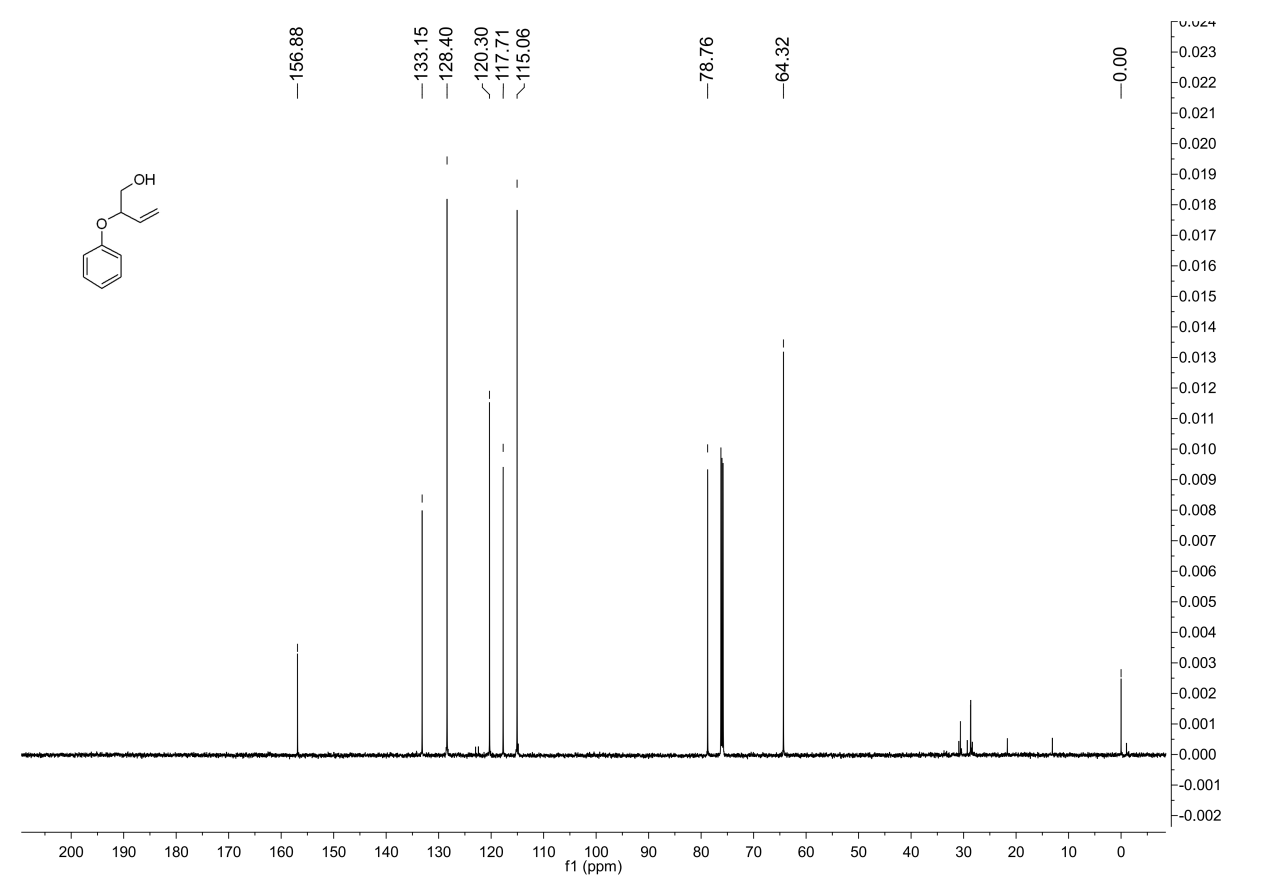
**

**
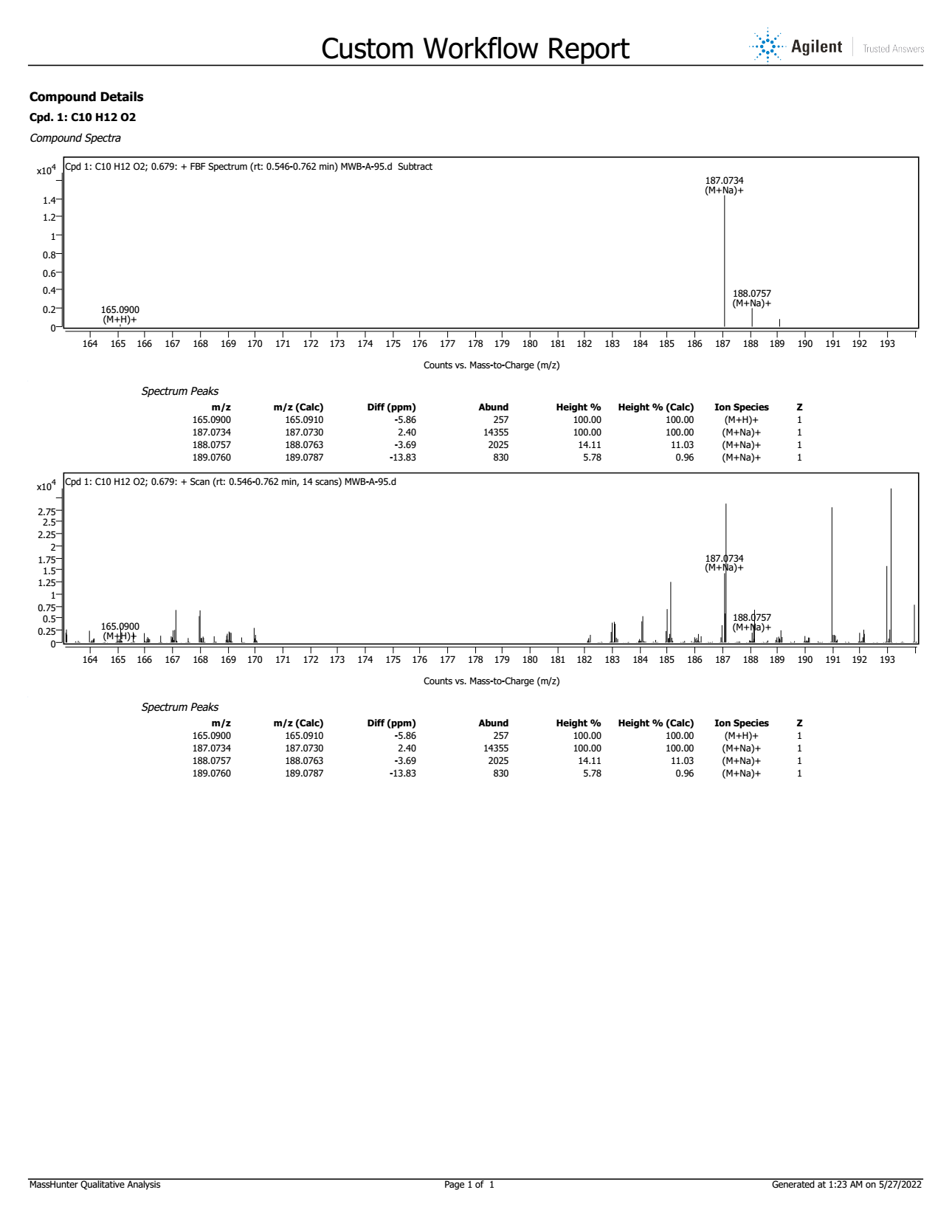
**

^1^H, ^13^C NMR and HRMS Spectra of Compound **3b**

**
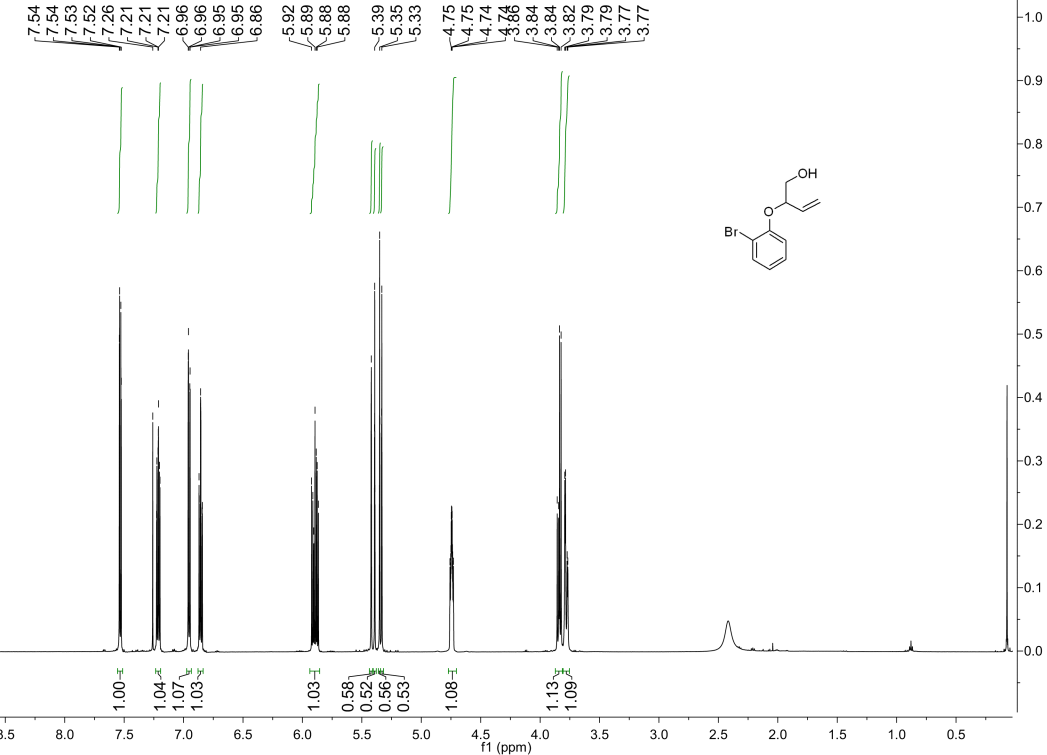
**

**
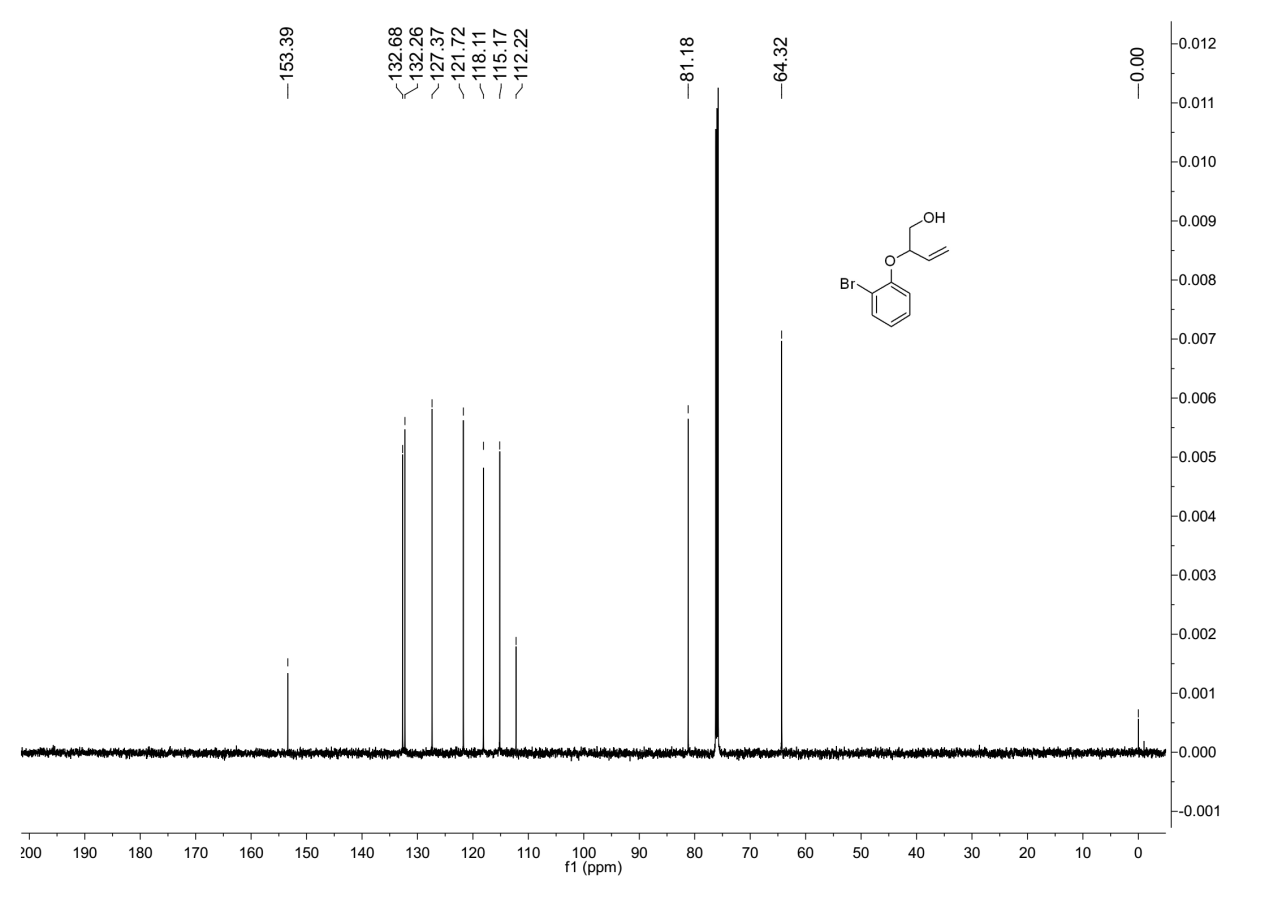
**

**
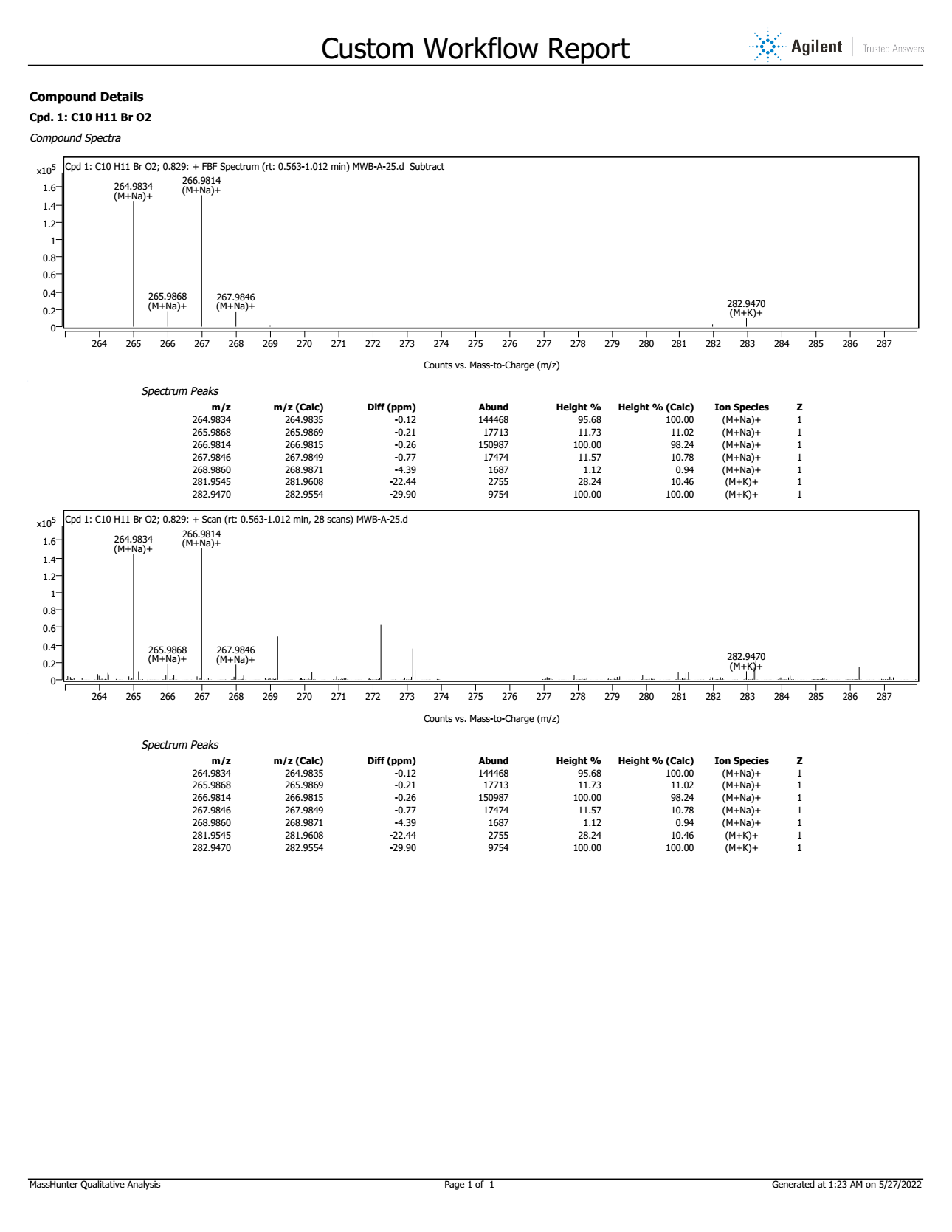
**

^1^H, ^13^C NMR and HRMS Spectra of Compound **3c**

**
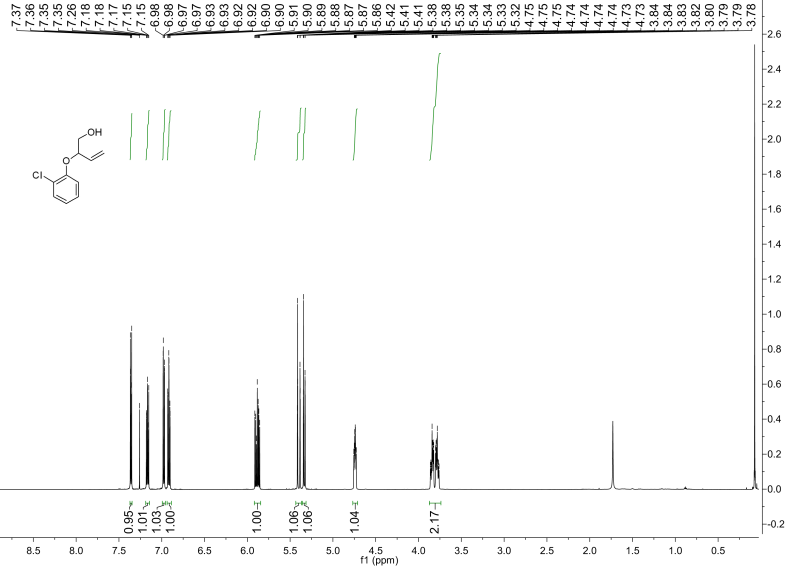
**

**
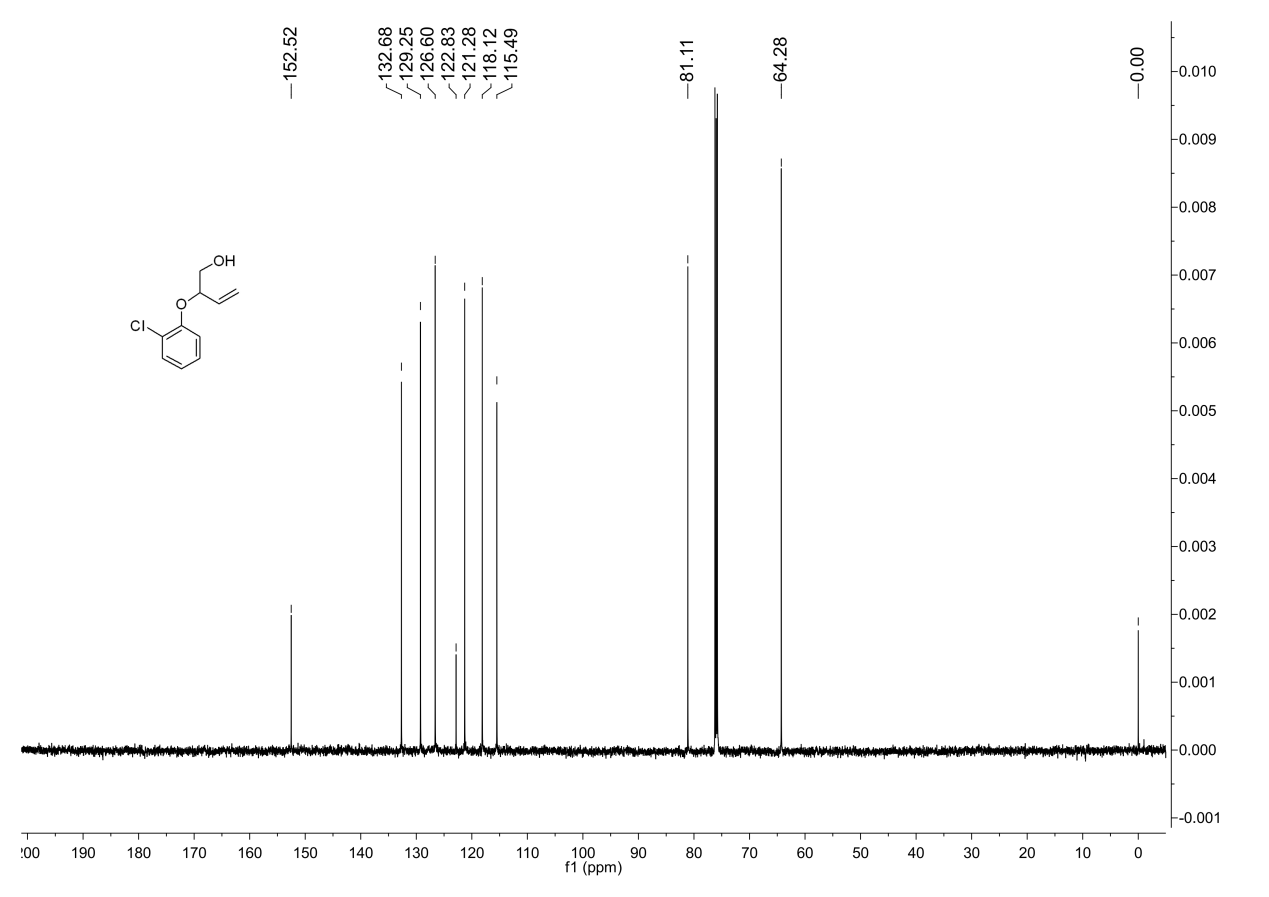
**

**
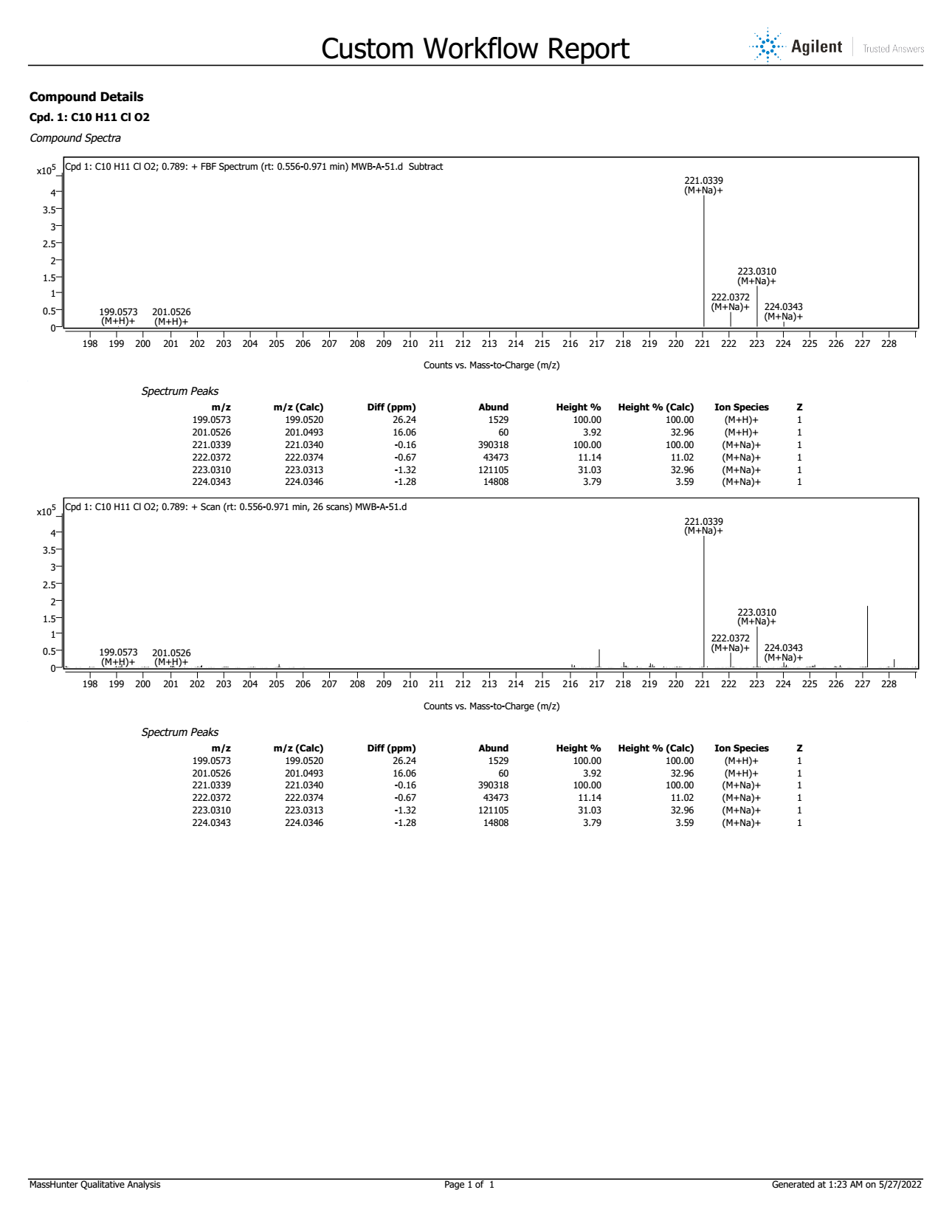
**

^1^H, ^13^C, ^19^F NMR and HRMS Spectra of Compound **3d**

**
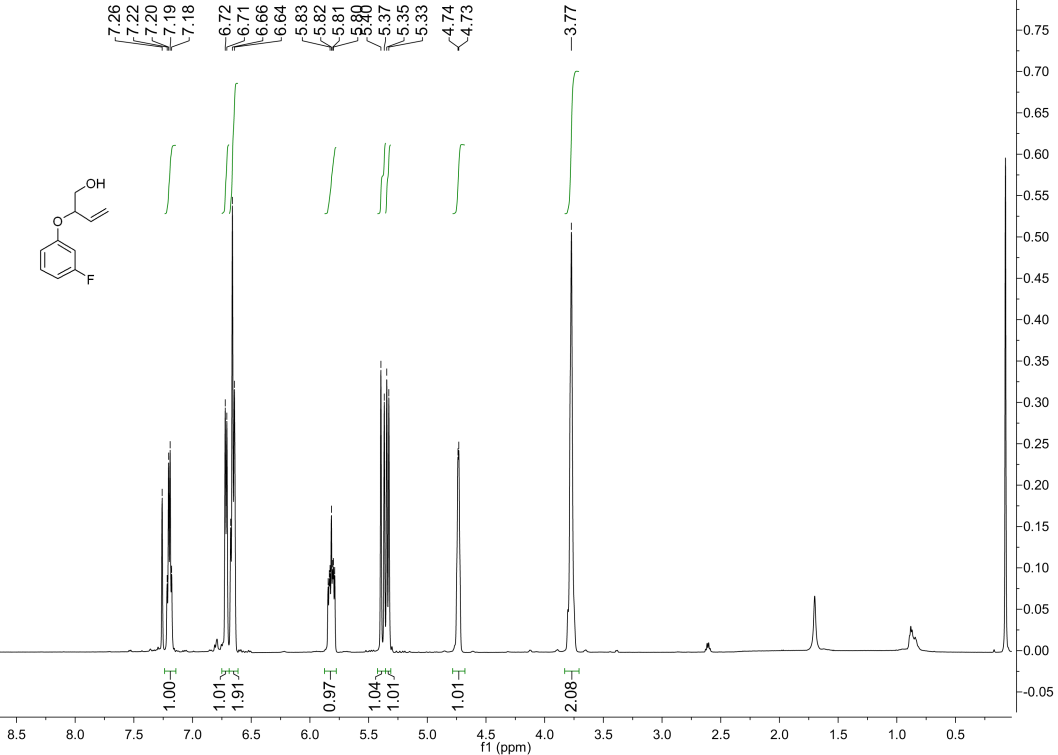
**

**
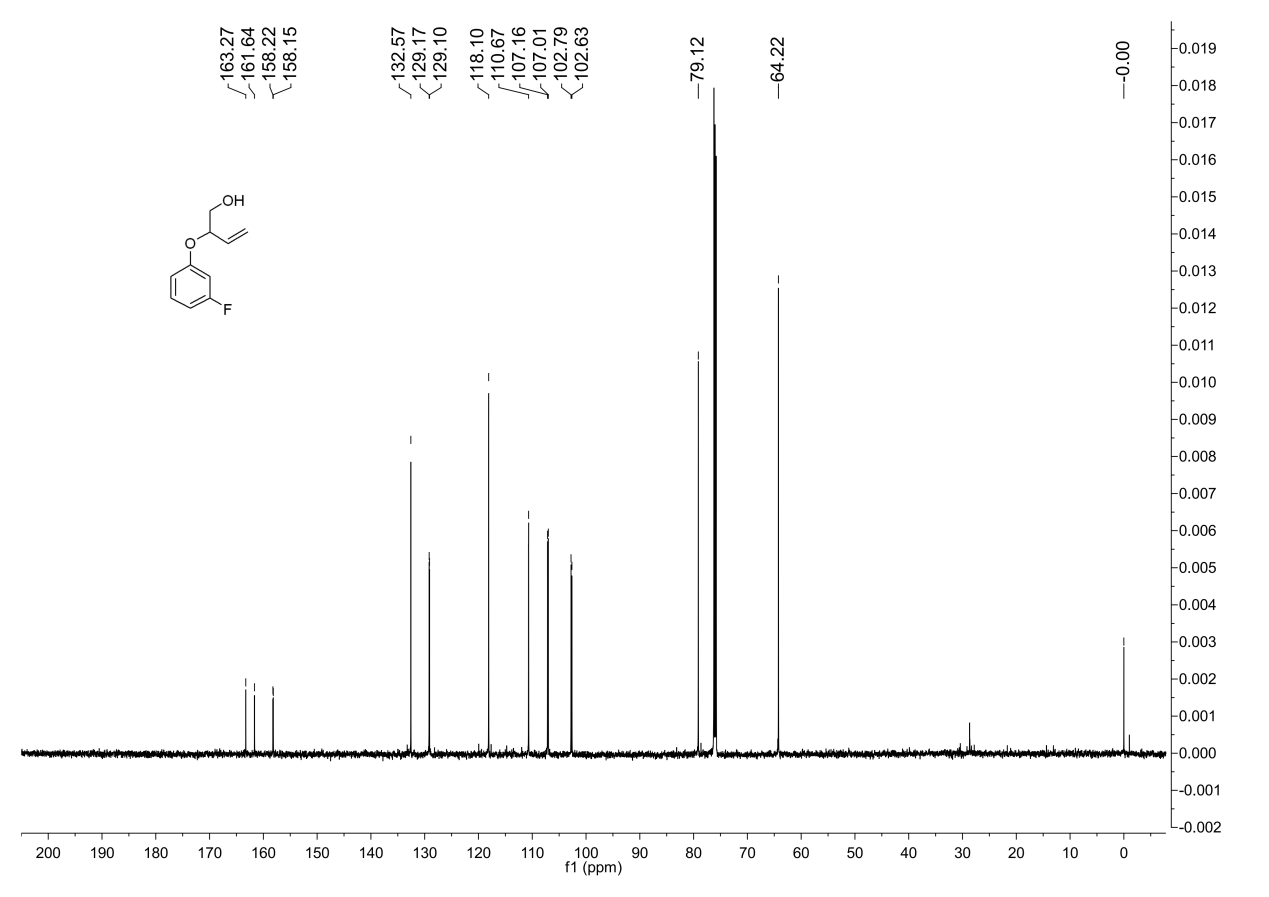
**

**
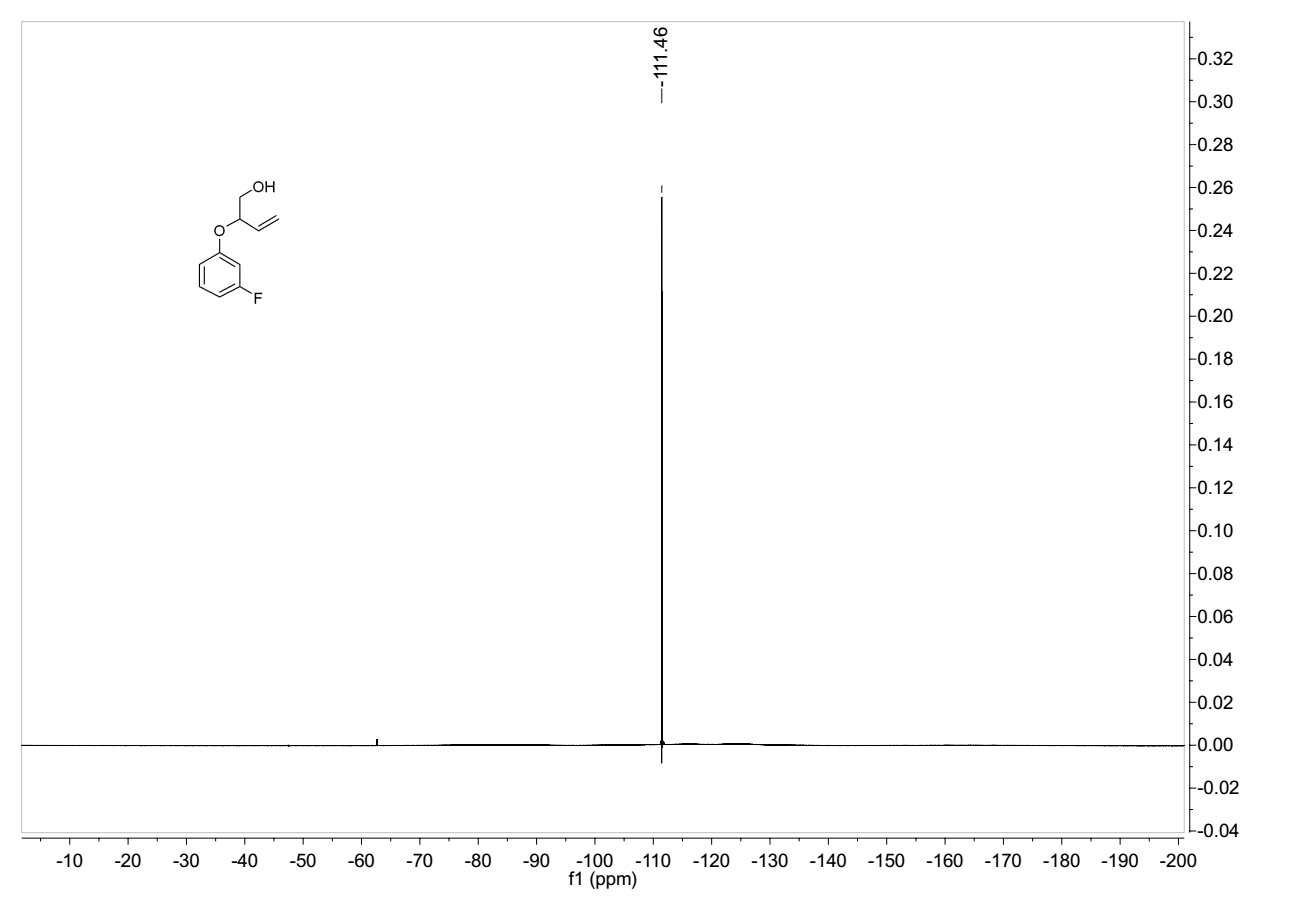
**

**
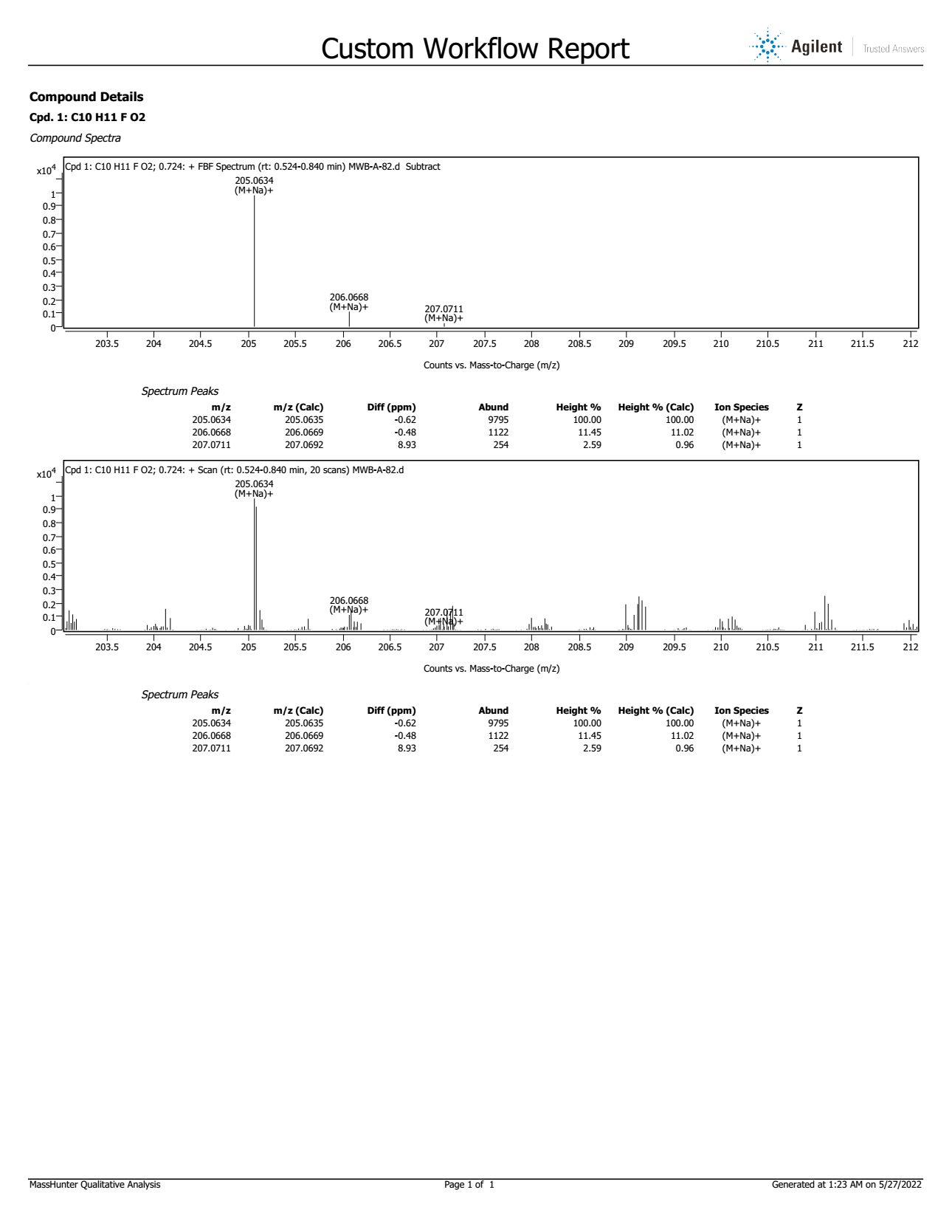
**

^1^H, ^13^C NMR and HRMS Spectra of Compound **3e**

**
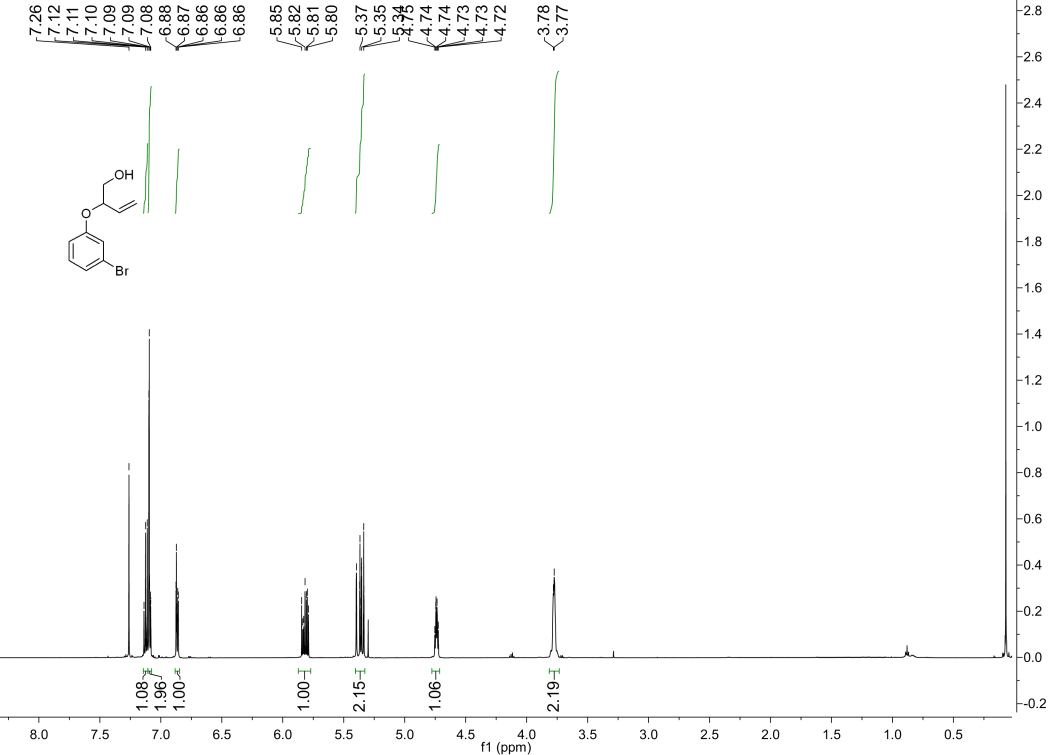
**

**
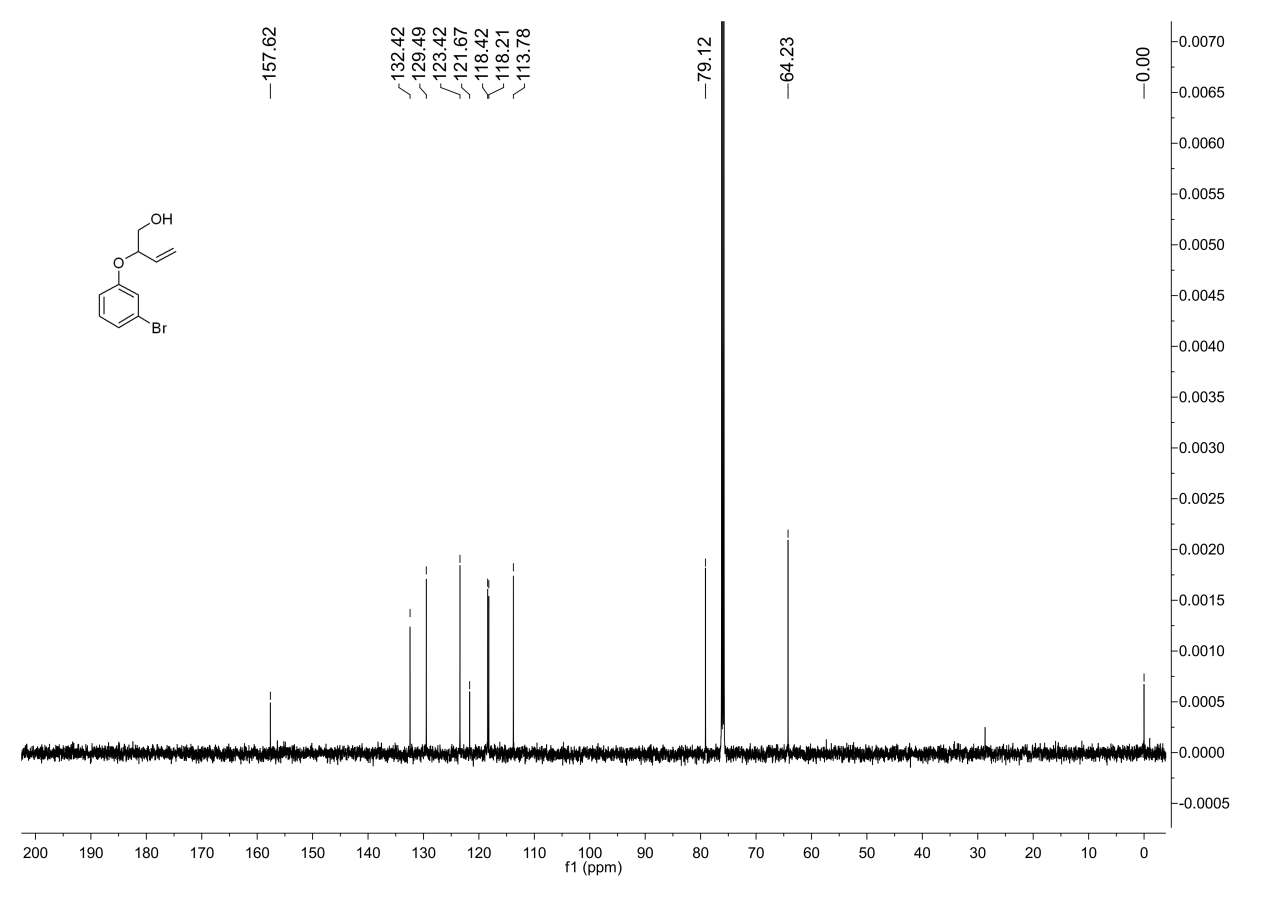
**

**
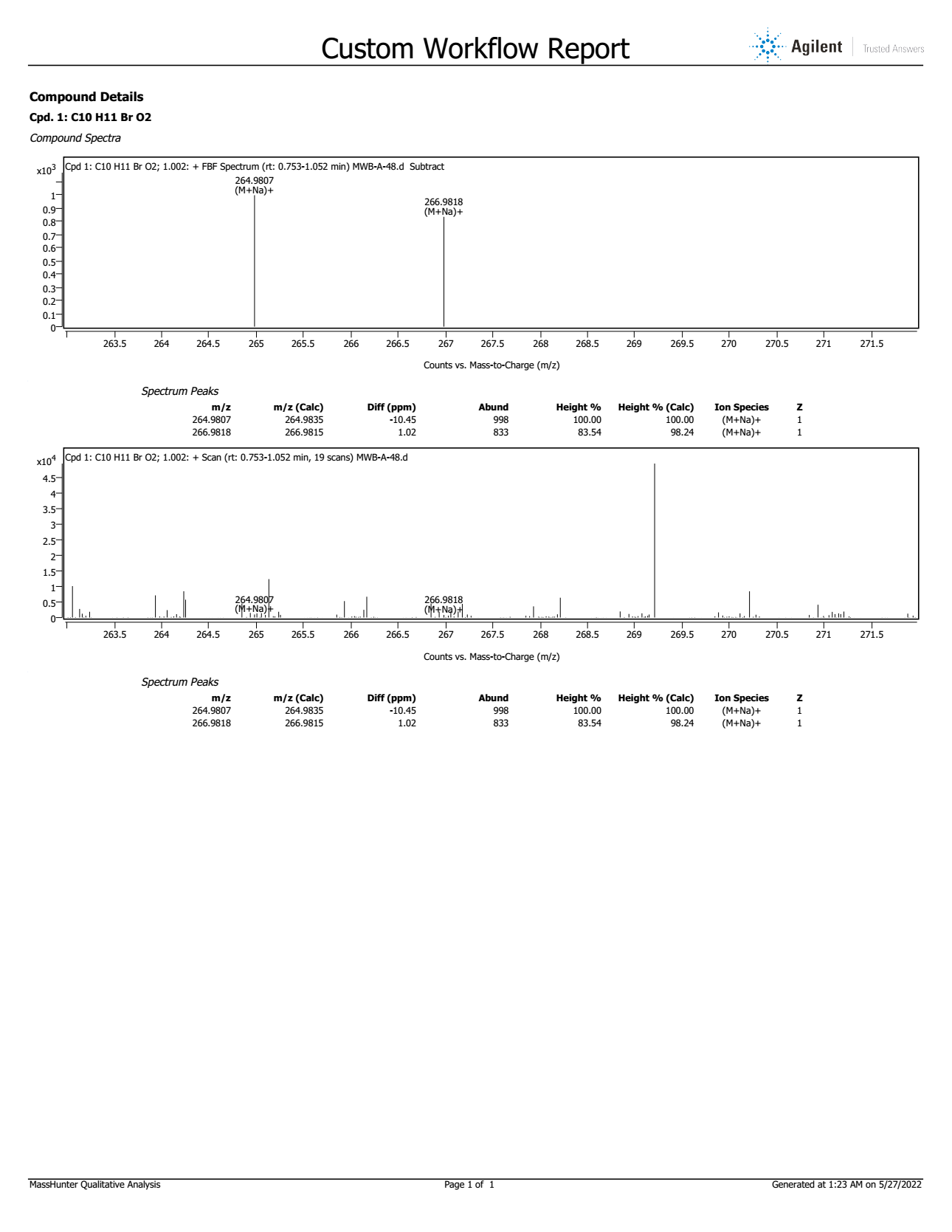
**

^1^H, ^13^C, ^19^F NMR and HRMS Spectra of Compound **3f**

**
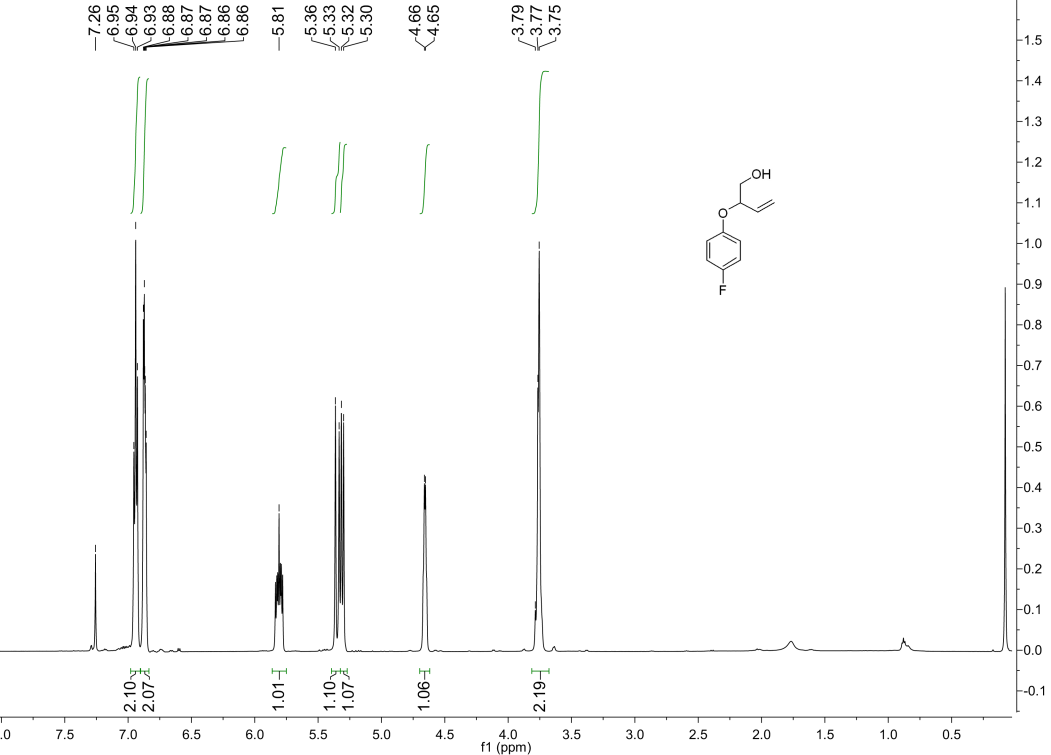
**

**
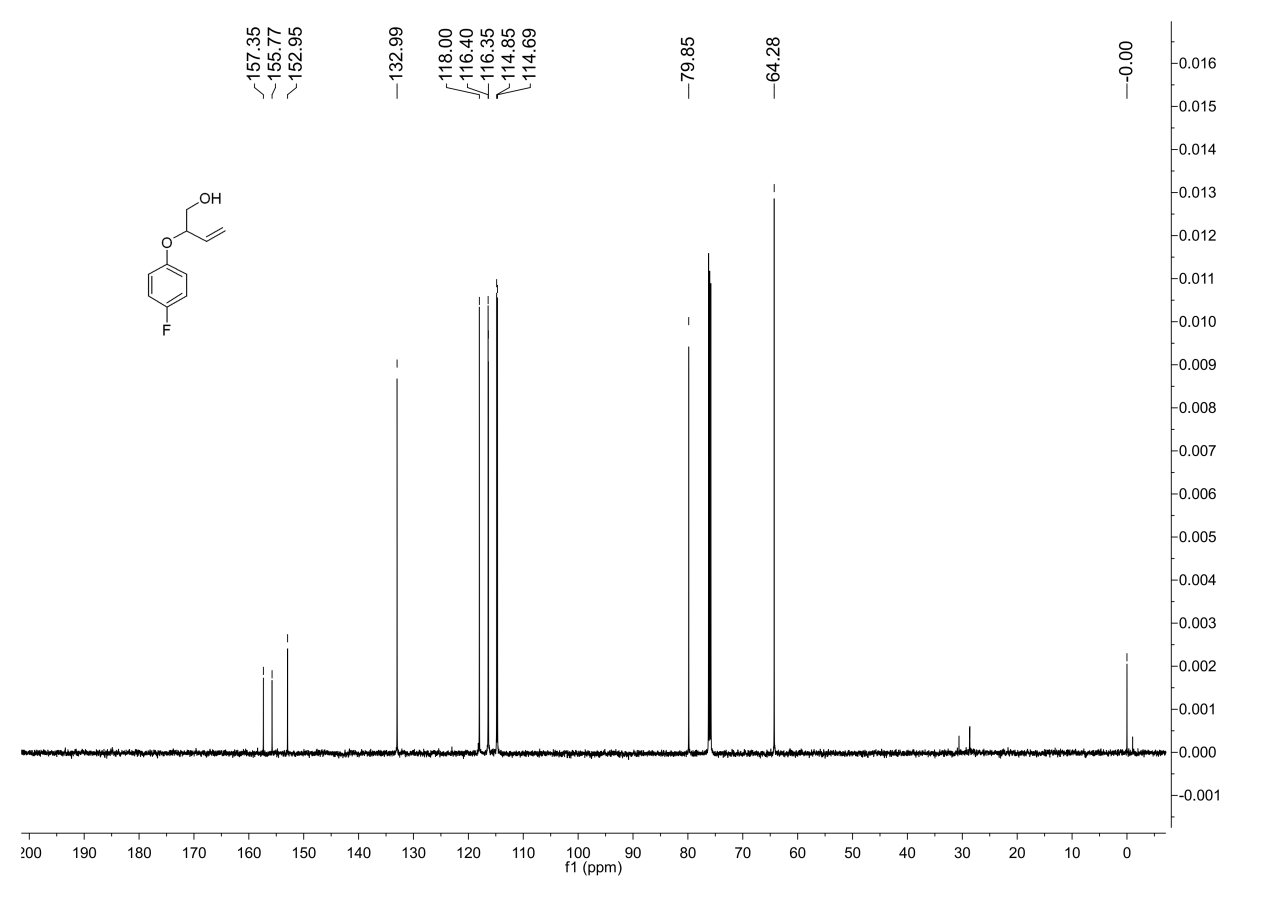
**

**
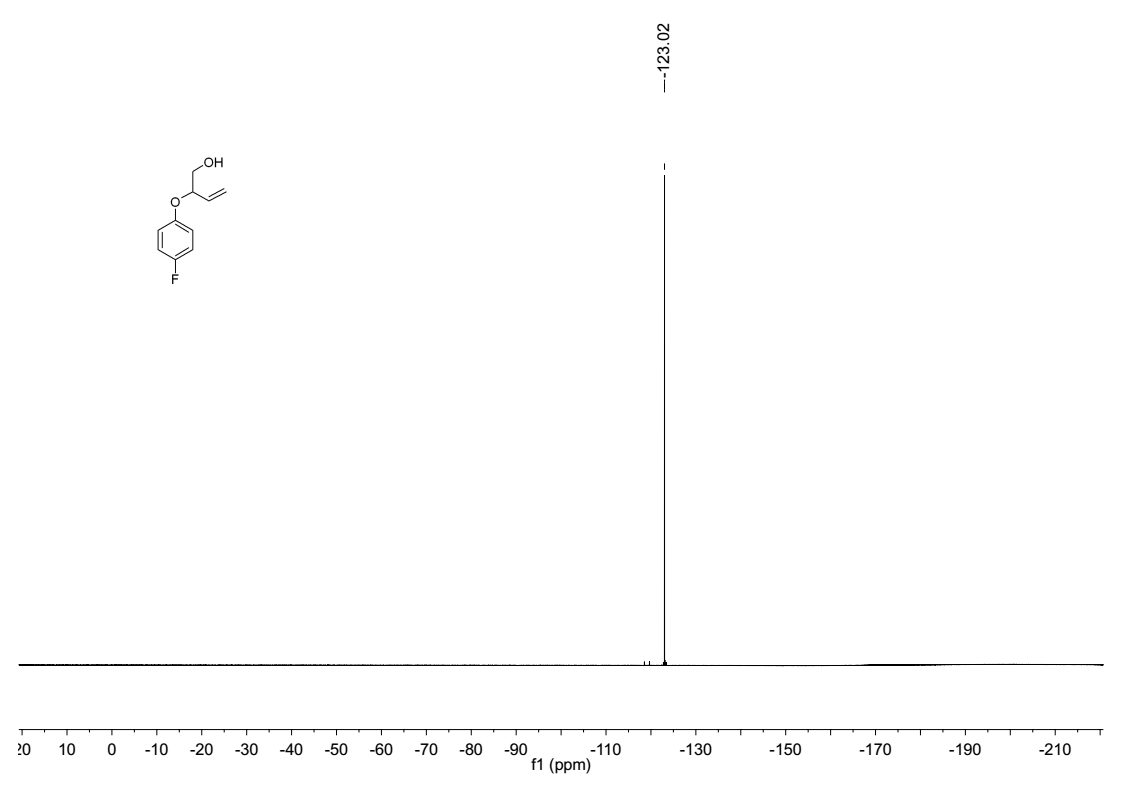
**

**
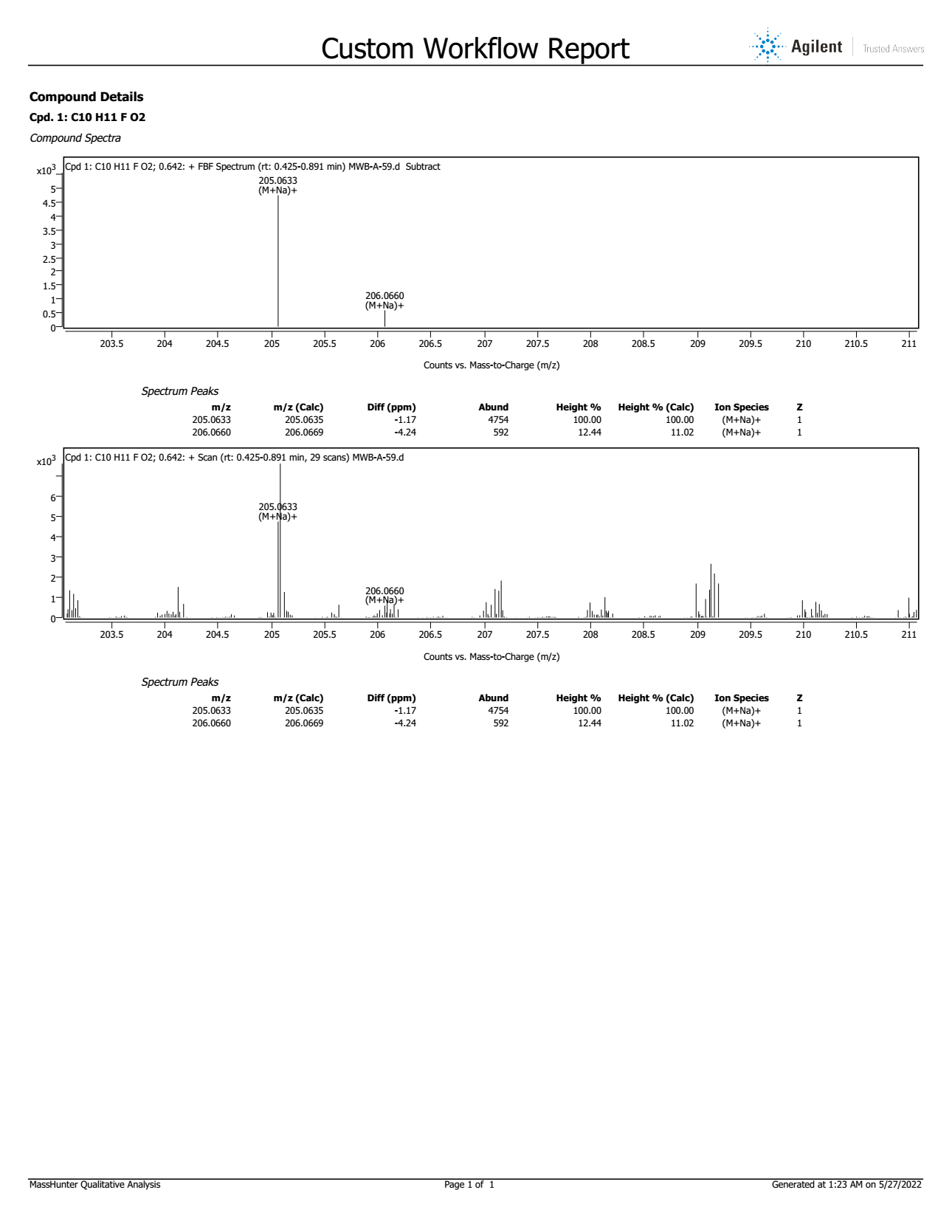
**

^1^H, ^13^C NMR and HRMS Spectra of Compound **3g**

**
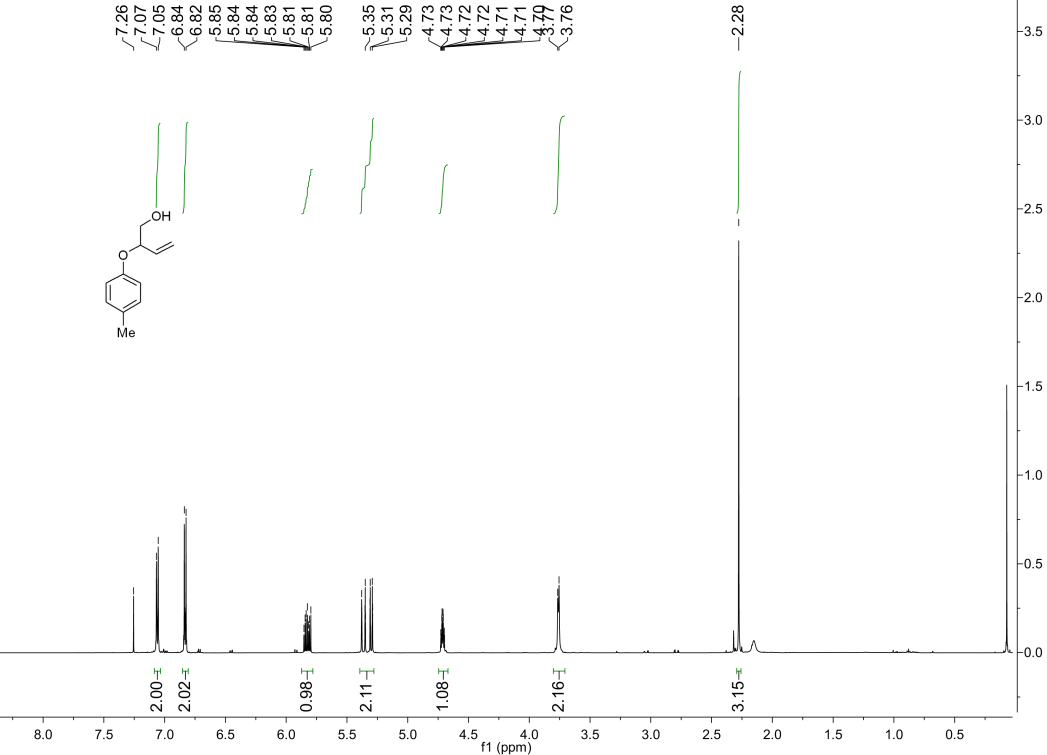
**

**
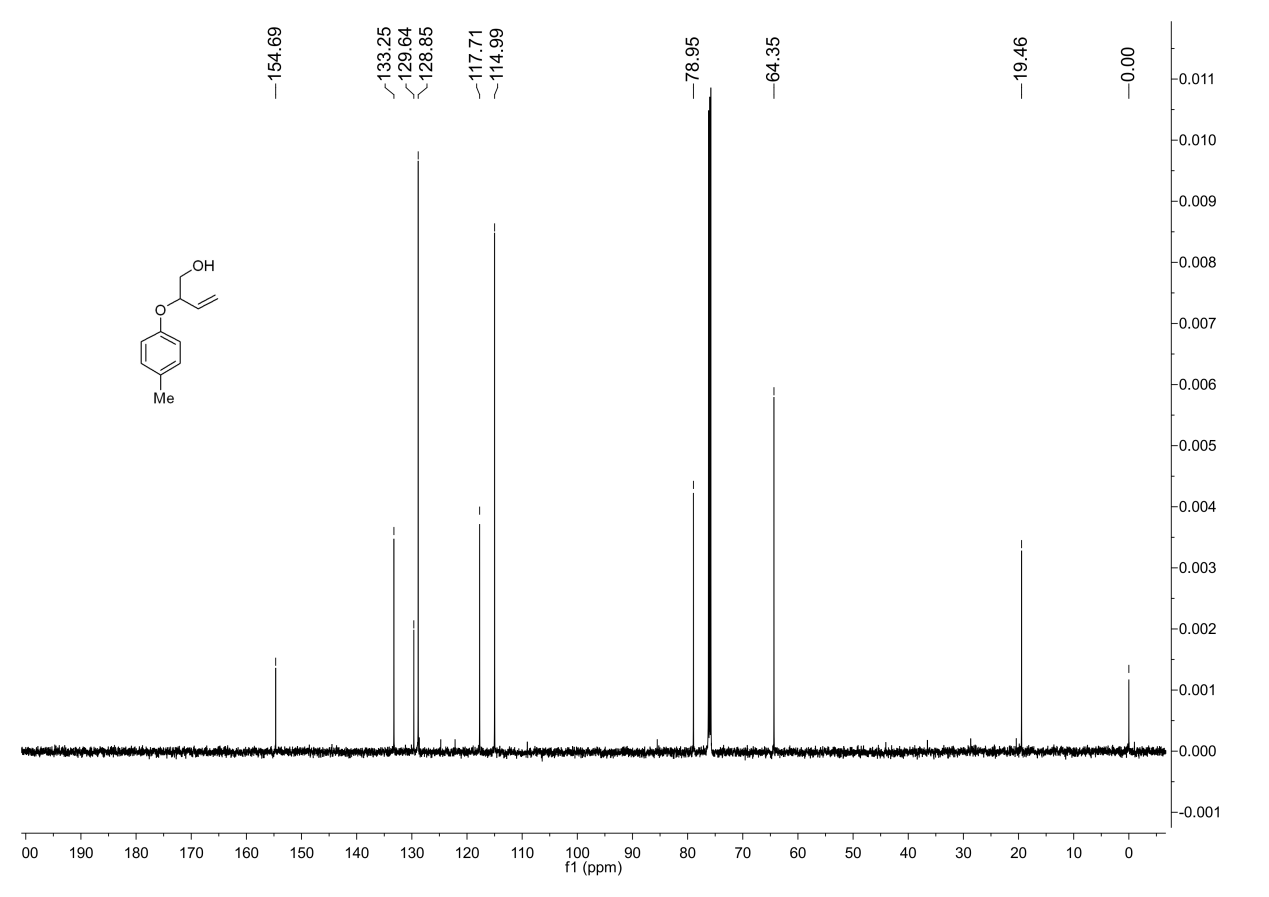
**

**
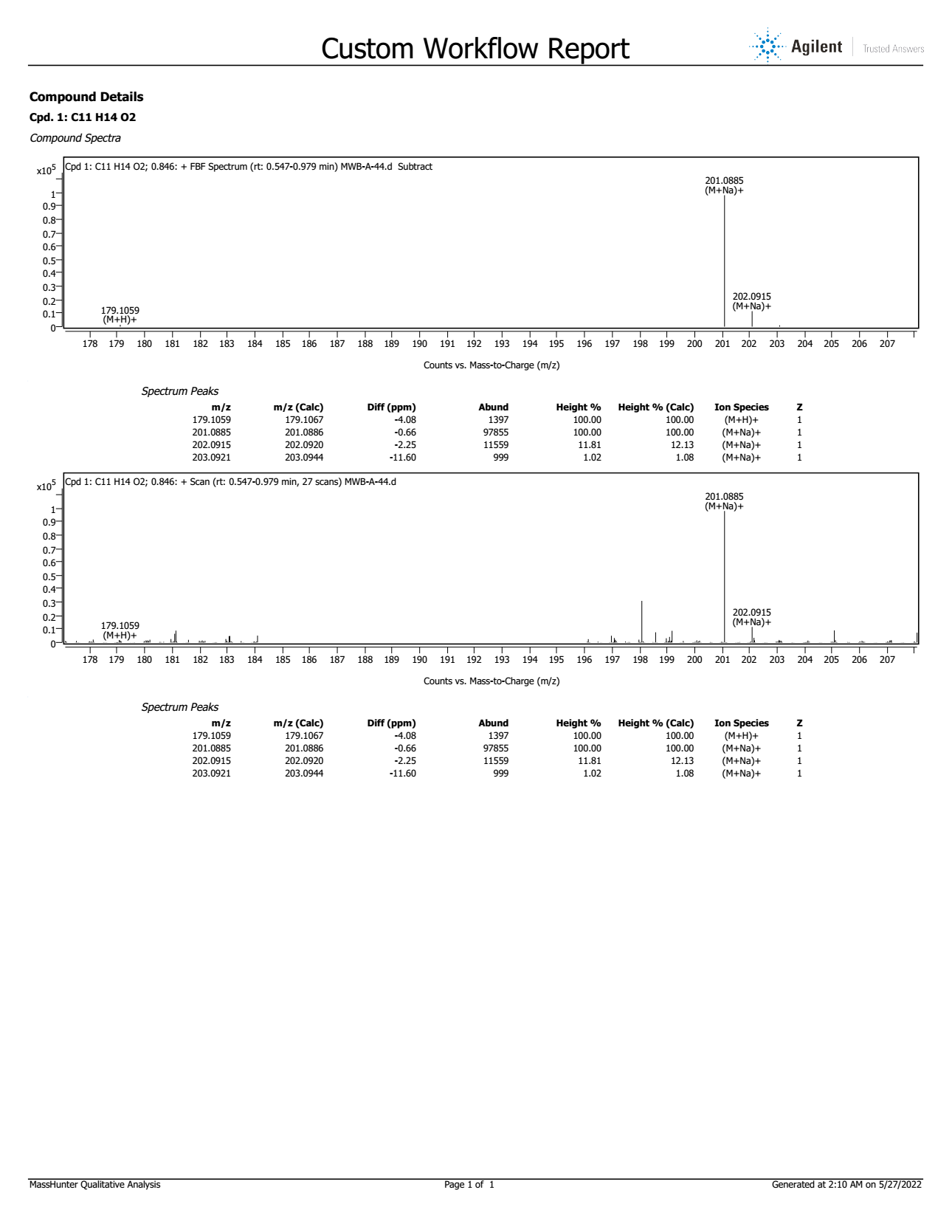
**

^1^H, ^13^C NMR and HRMS Spectra of Compound **3h**

**
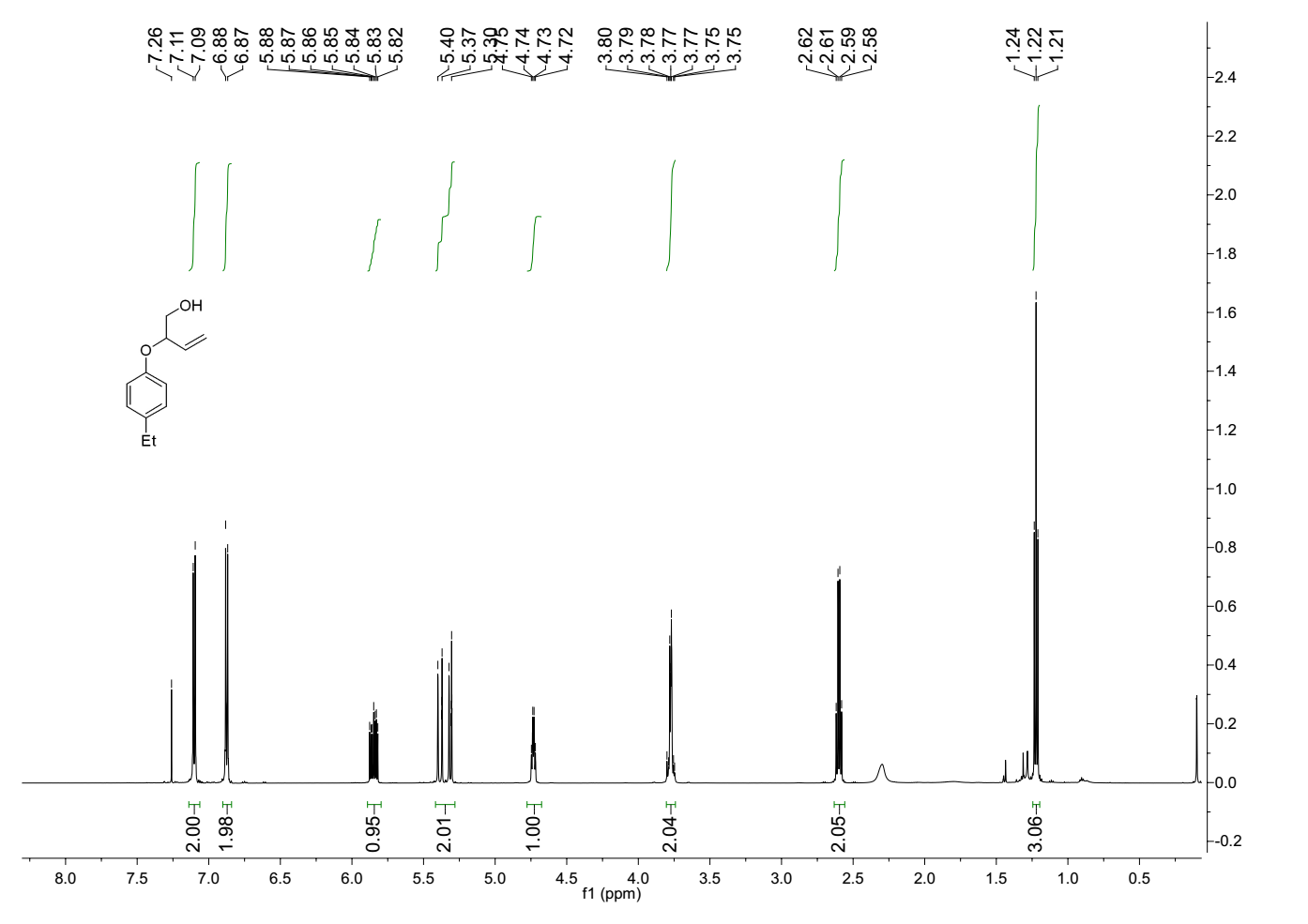
**

**
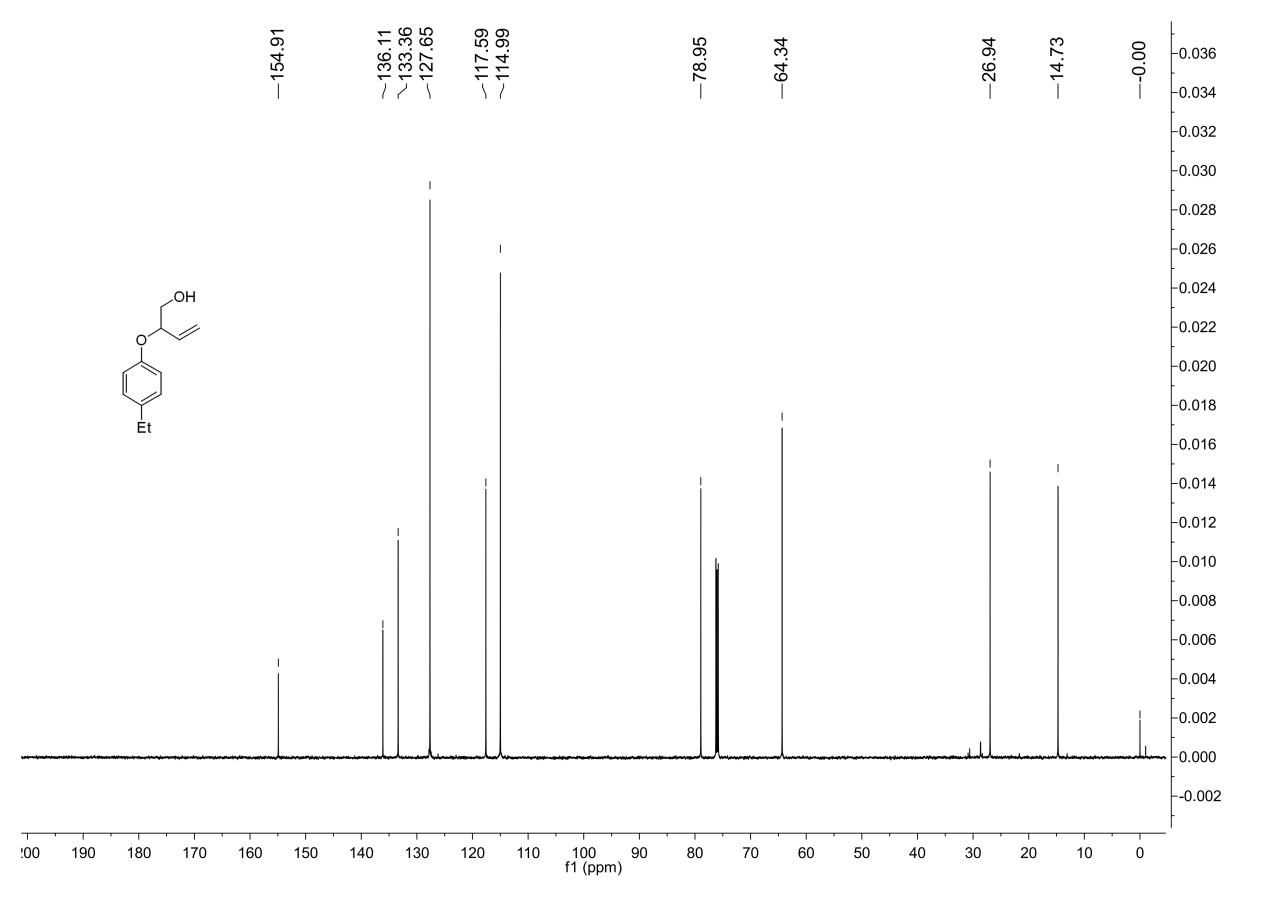
**

**
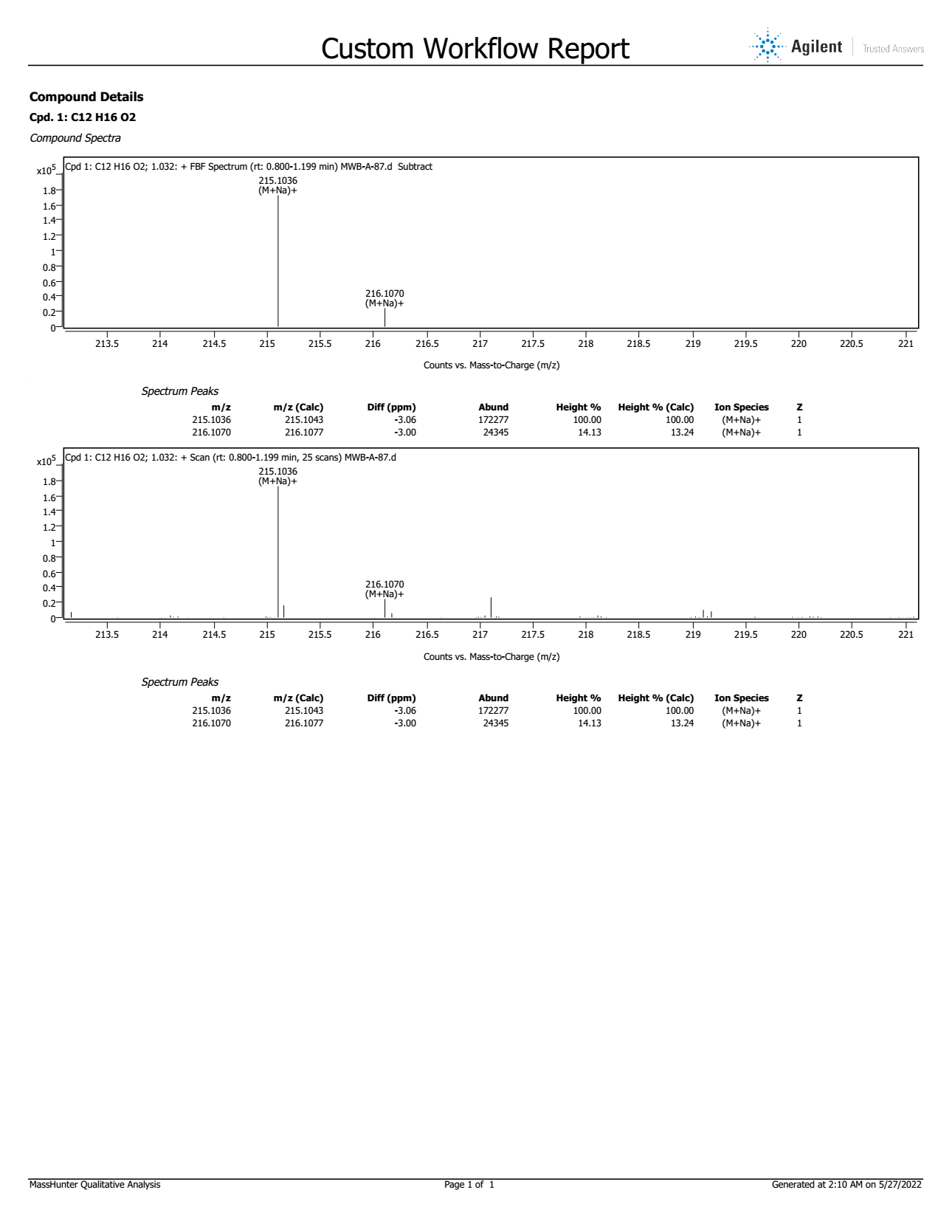
**

^1^H, ^13^C NMR and HRMS Spectra of Compound **3i**

**
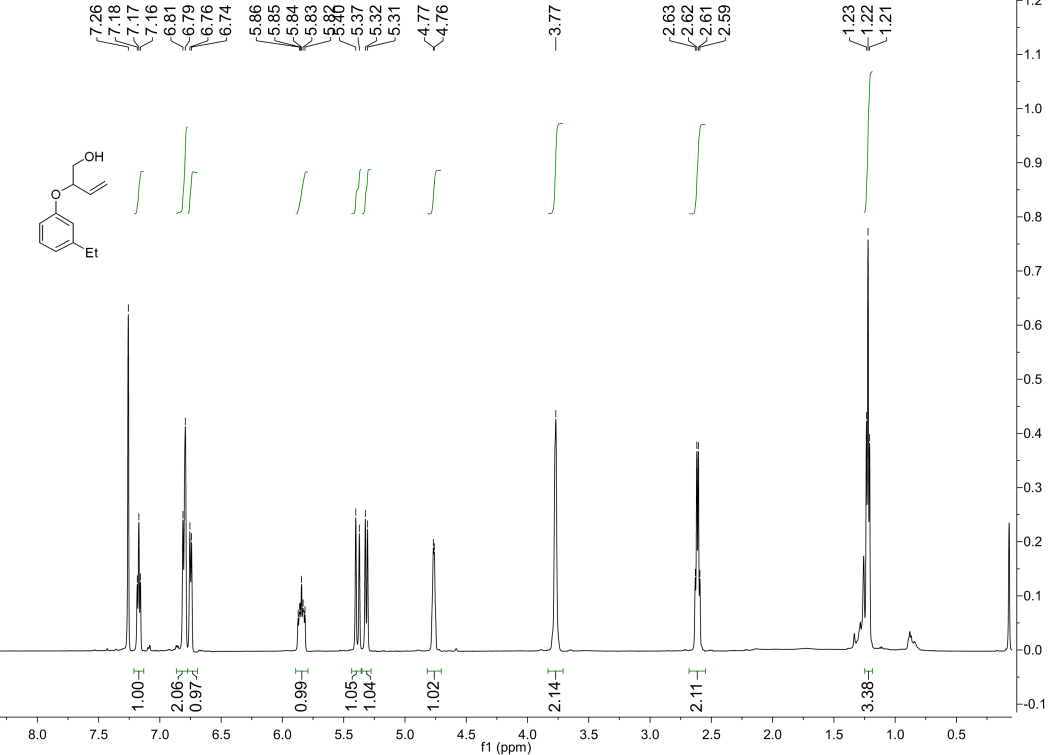
**

**
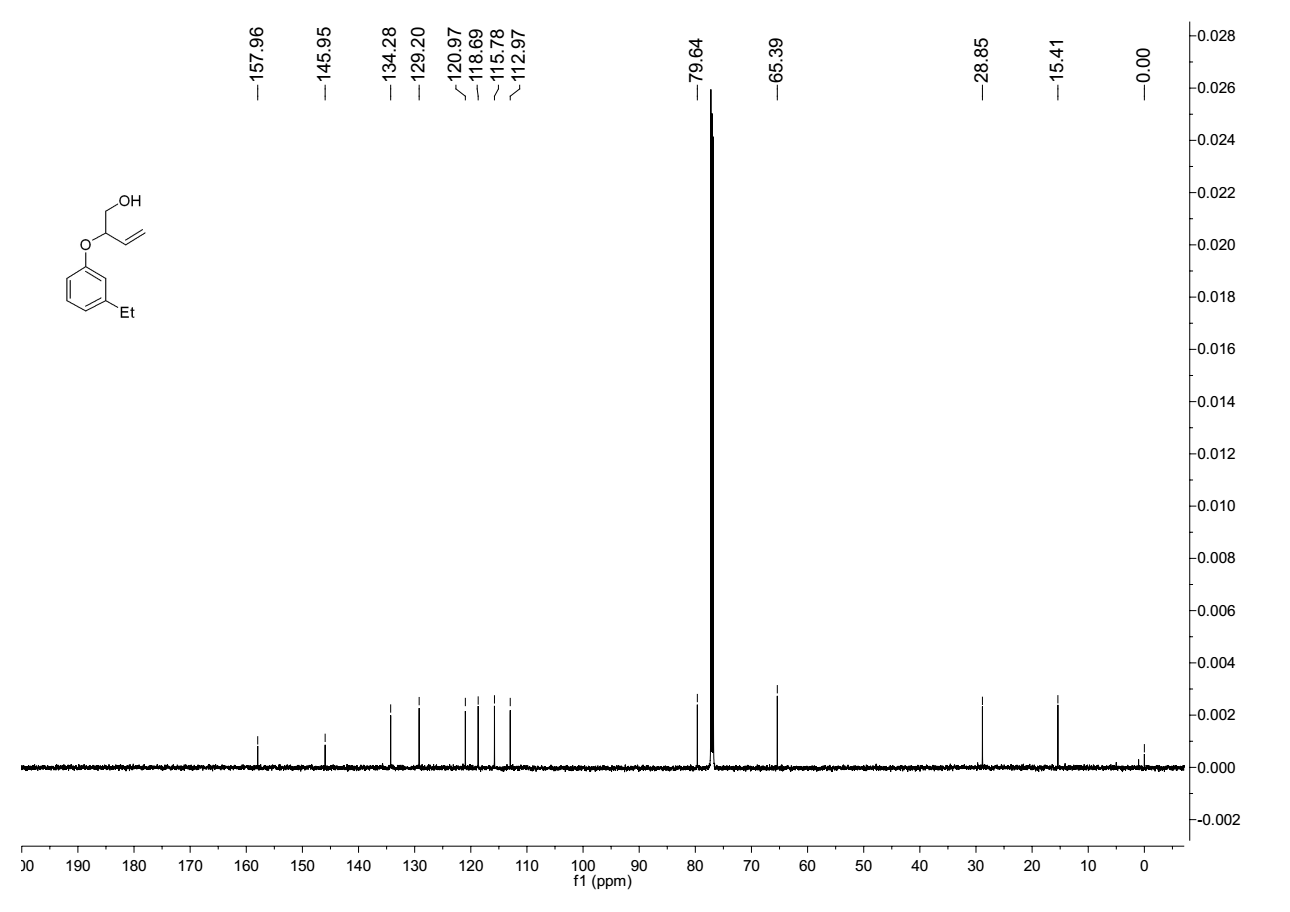
**

**
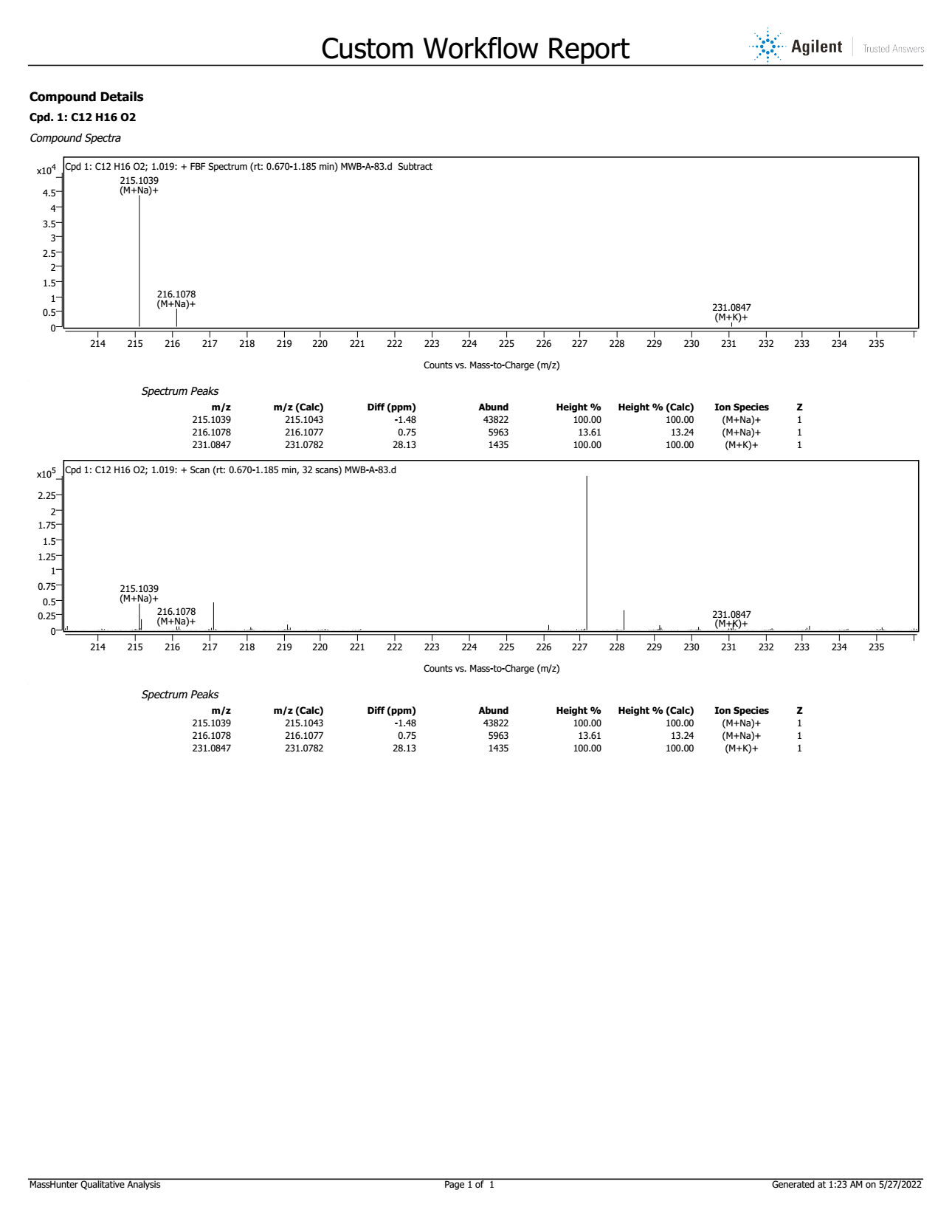
**

^1^H, ^13^C NMR and HRMS Spectra of Compound **3j**

**
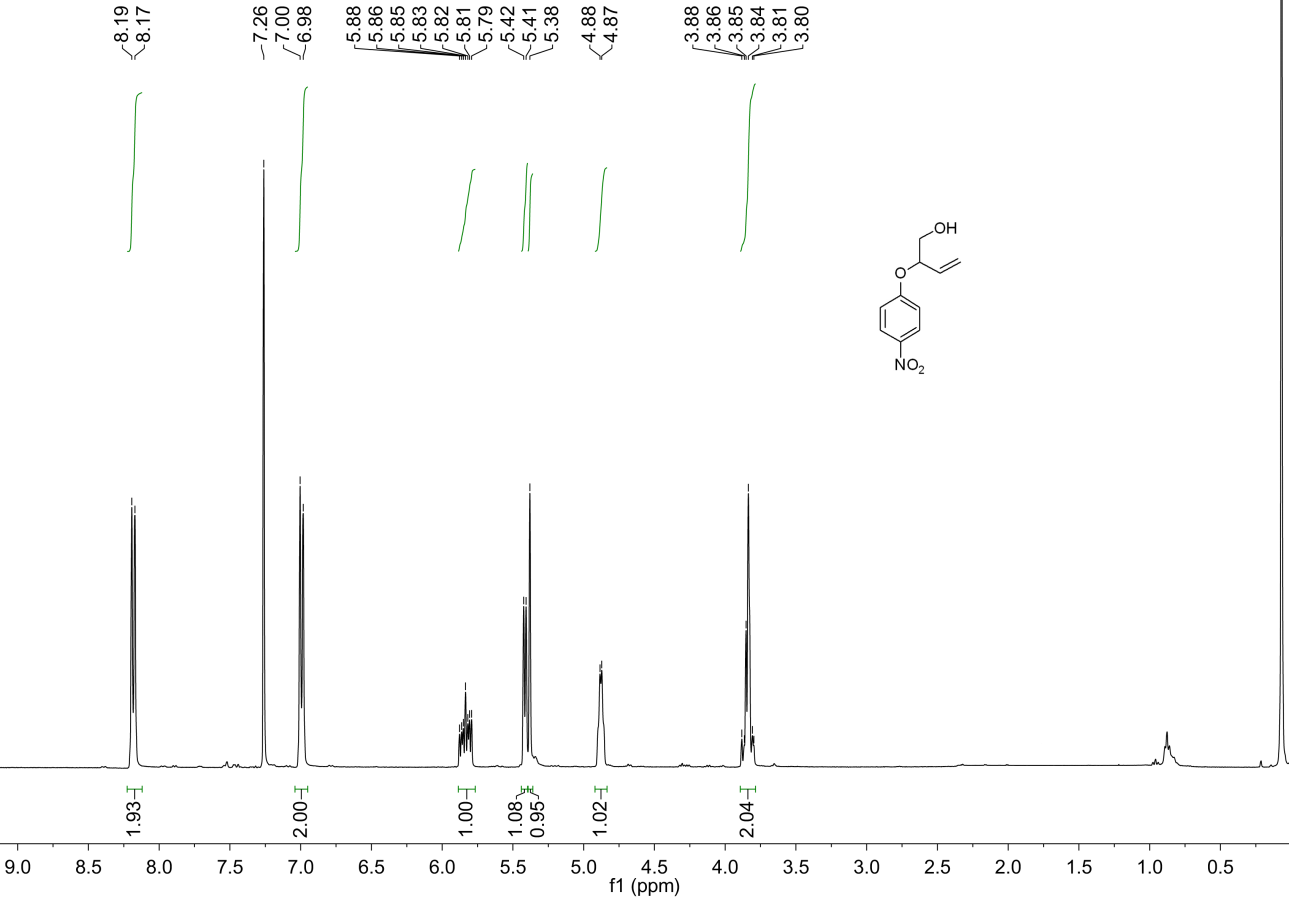
**

**
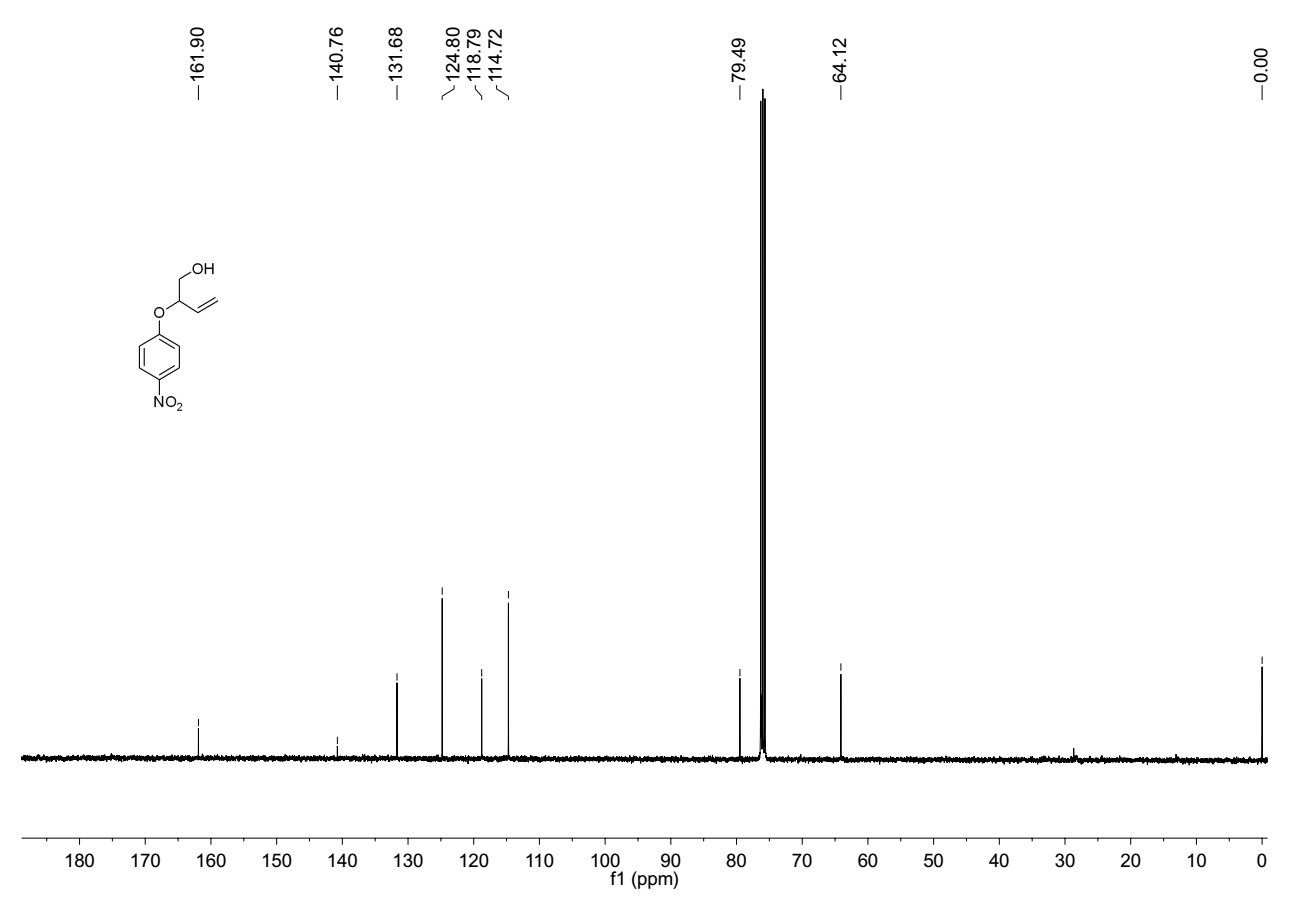
**

**
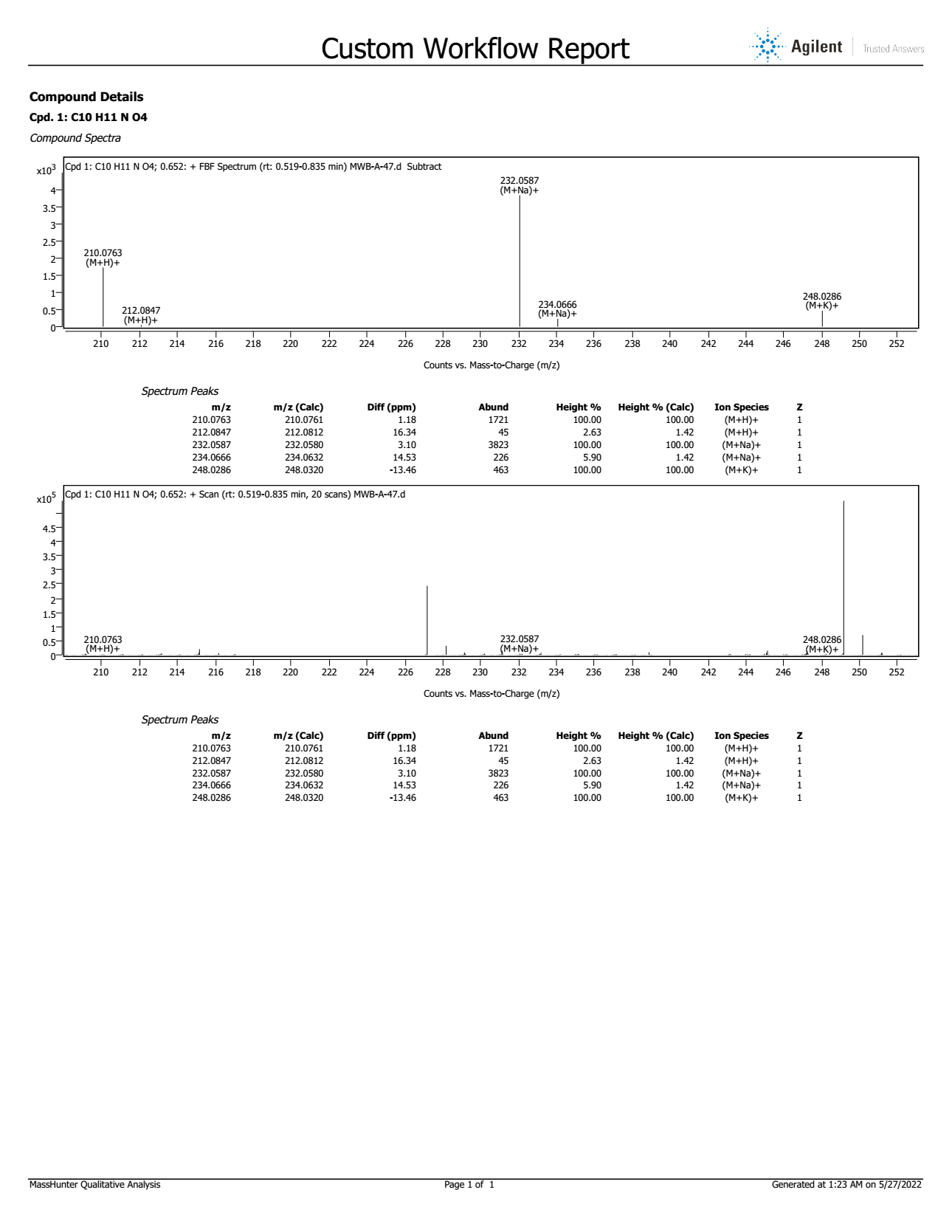
**

^1^H, ^13^C NMR and HRMS Spectra of Compound **3k**

**
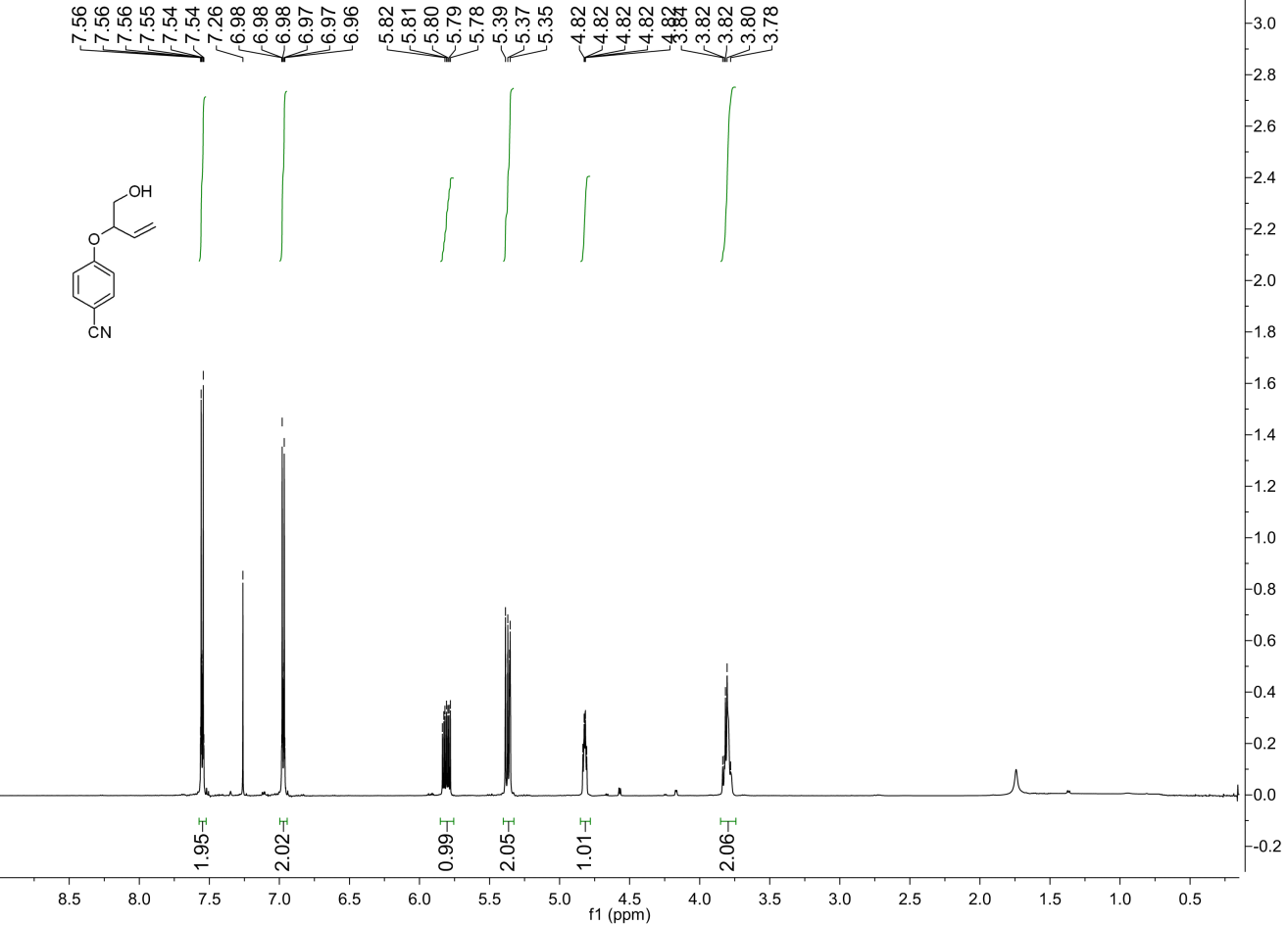
**

**
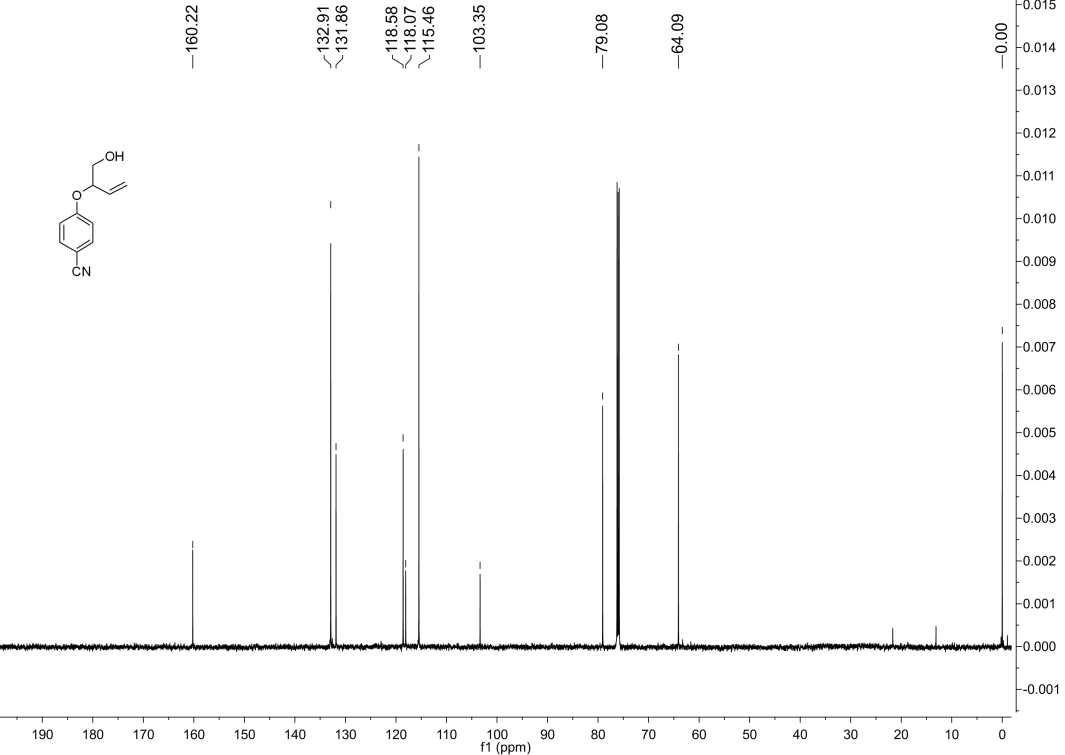
**

**
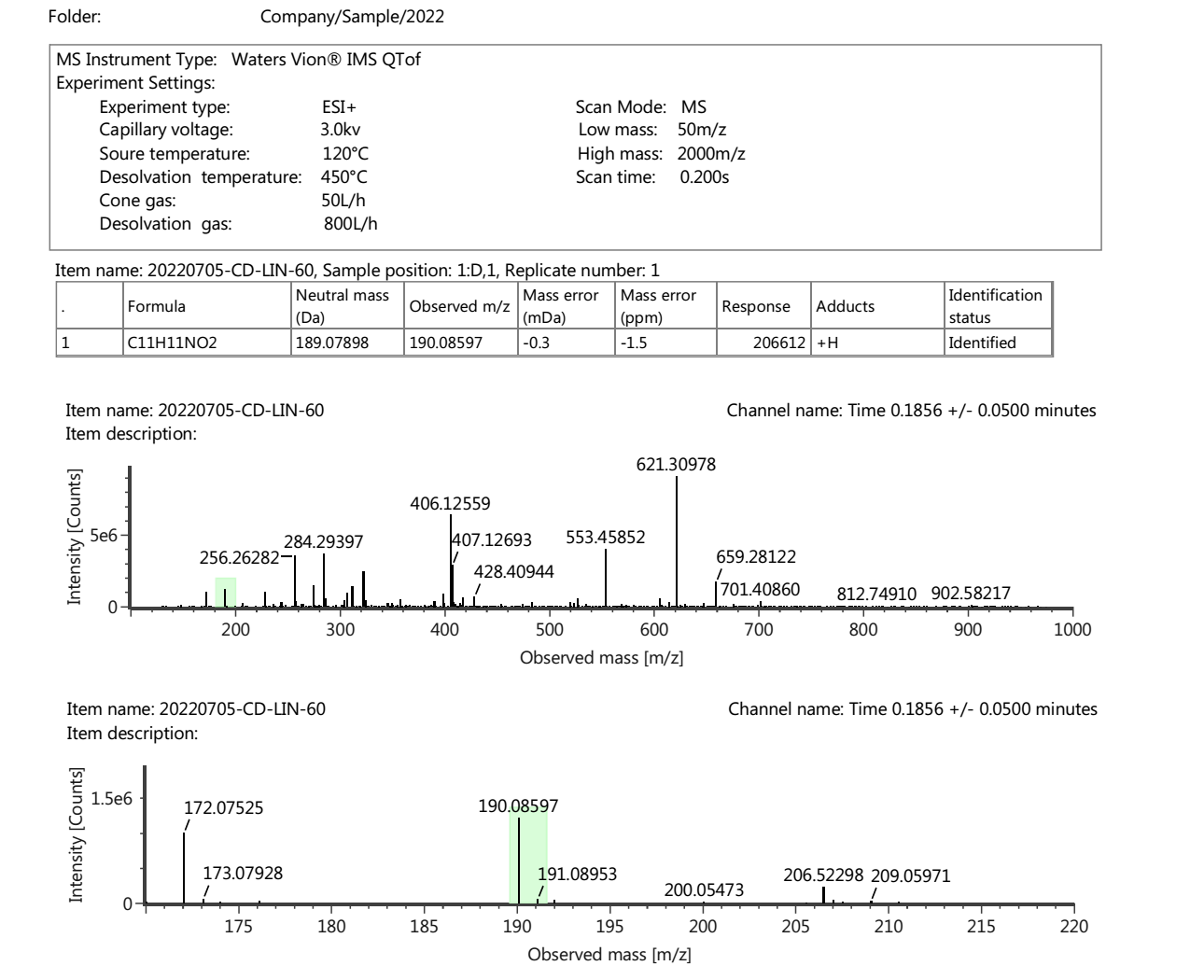
**

^1^H, ^13^C NMR and HRMS Spectra of Compound **3l**

**
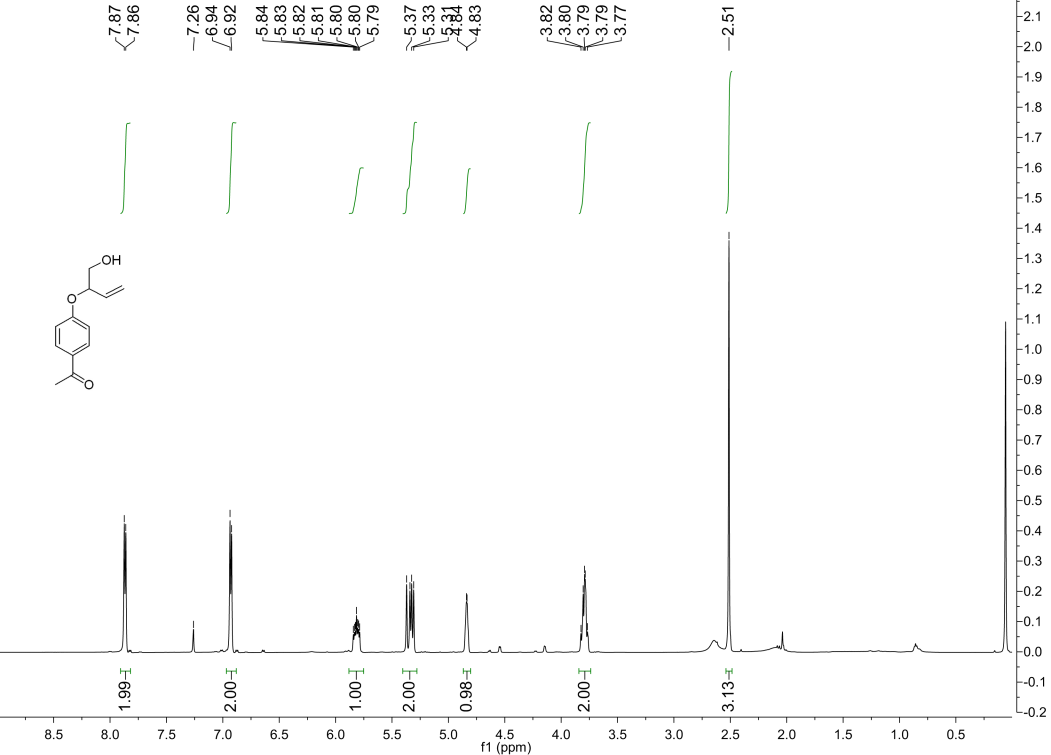
**

**
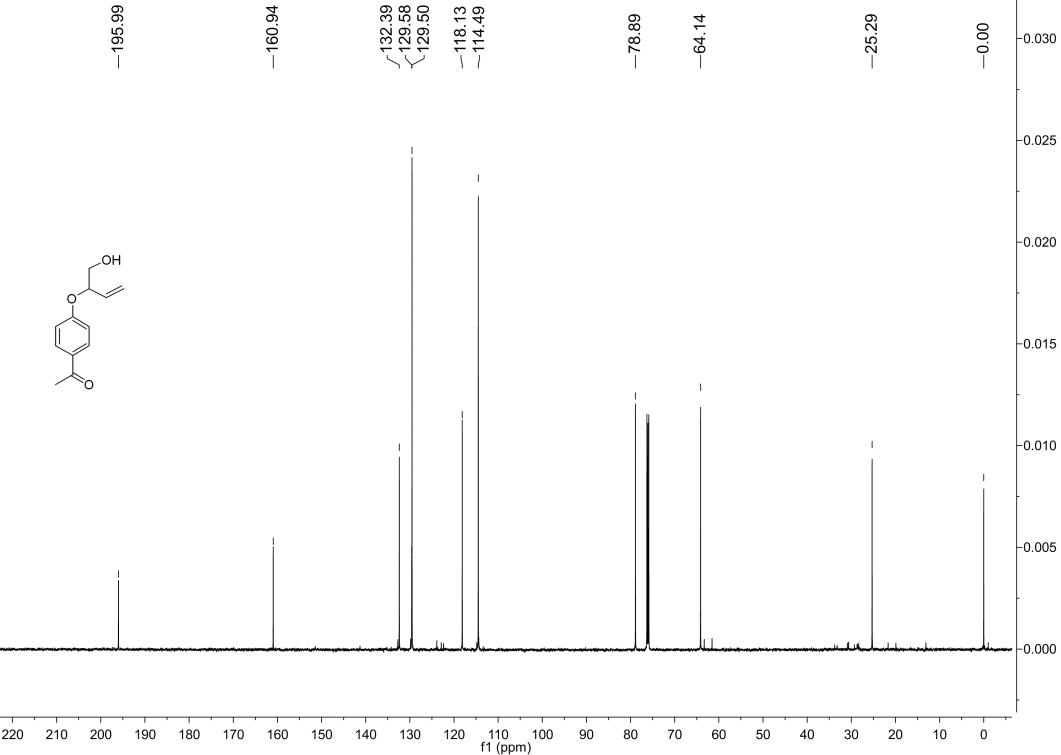
**

**
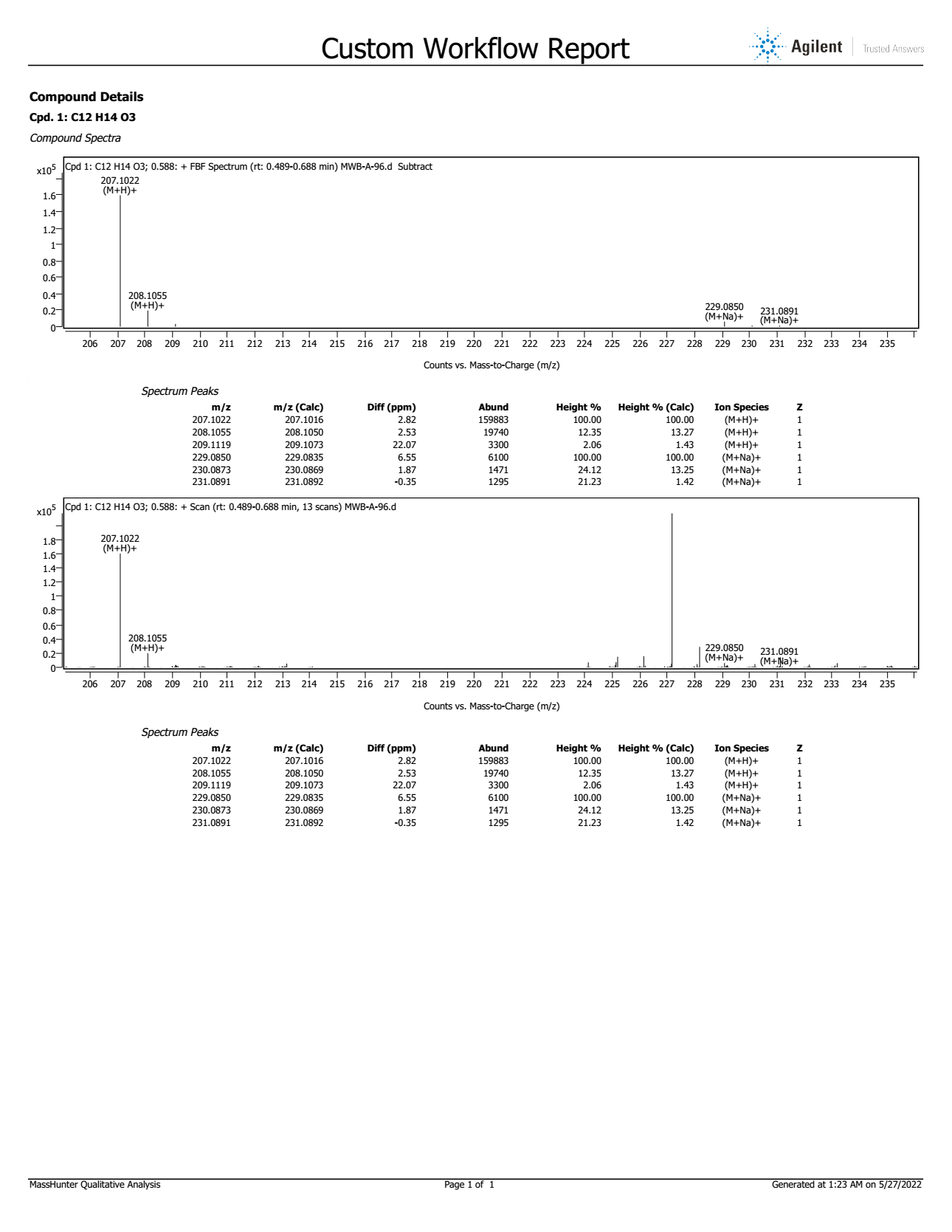
**

^1^H, ^13^C NMR and HRMS Spectra of Compound **3m**

**
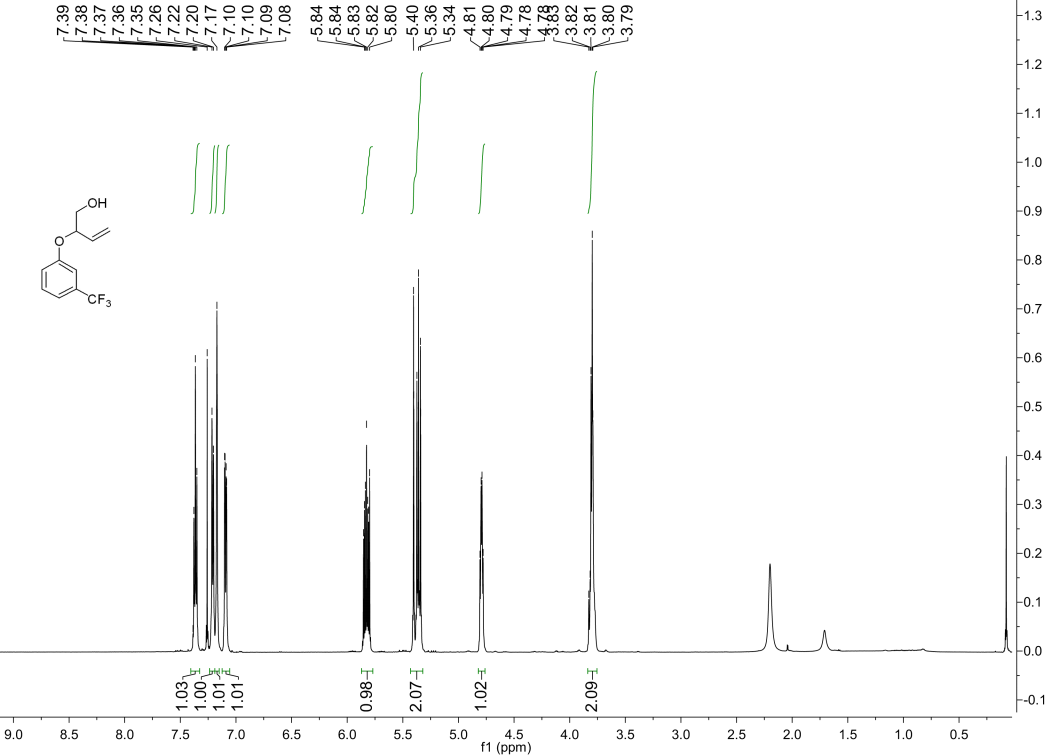
**

**
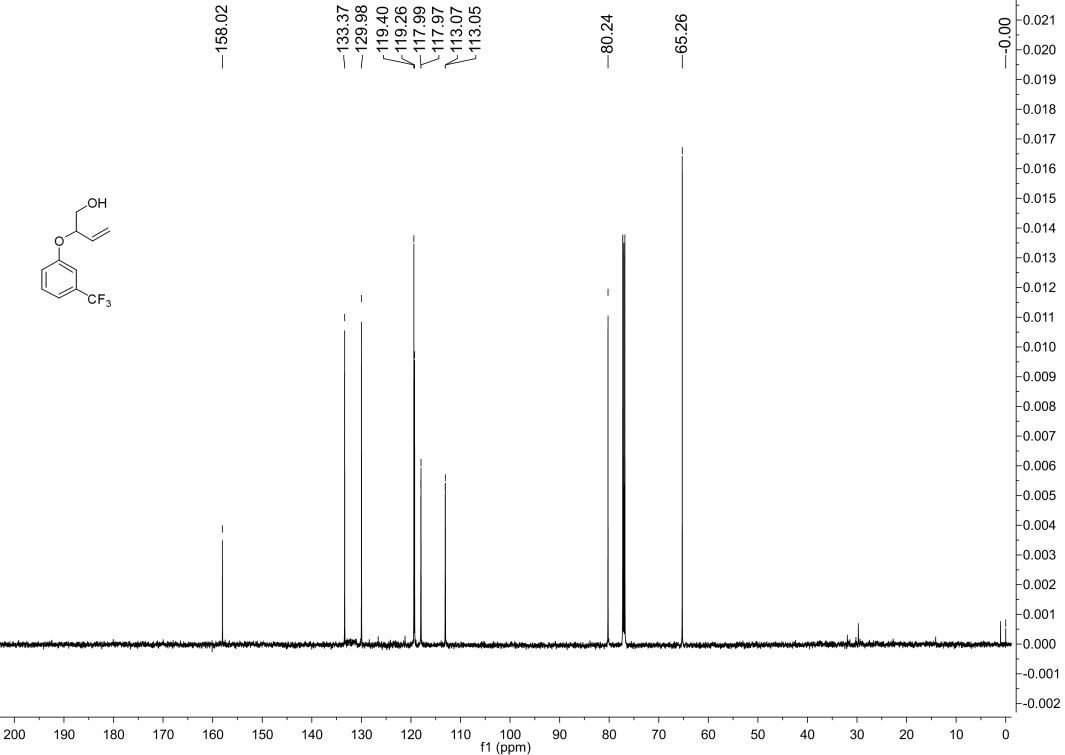
**

**
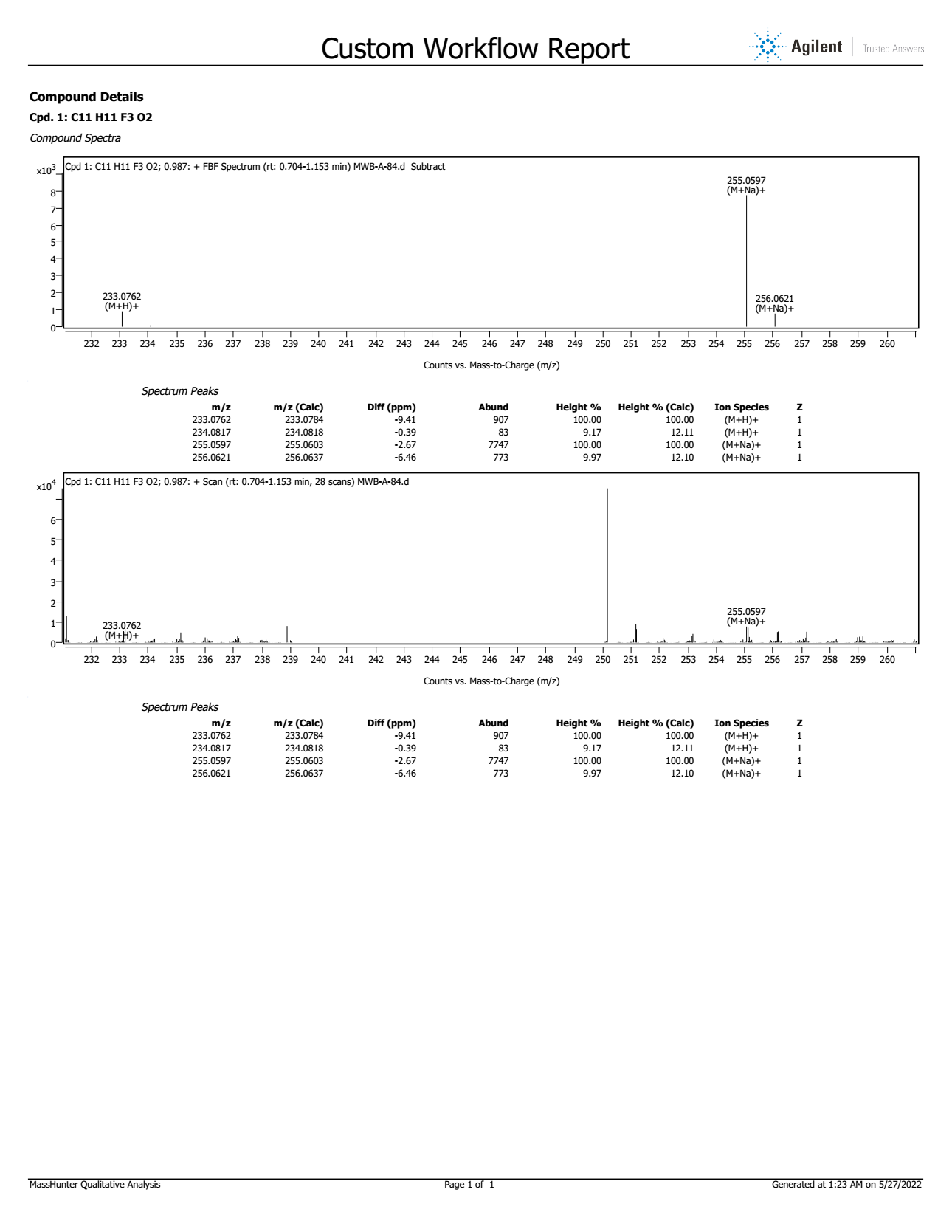
**

^1^H, ^13^C NMR and HRMS Spectra of Compound **3n**

**
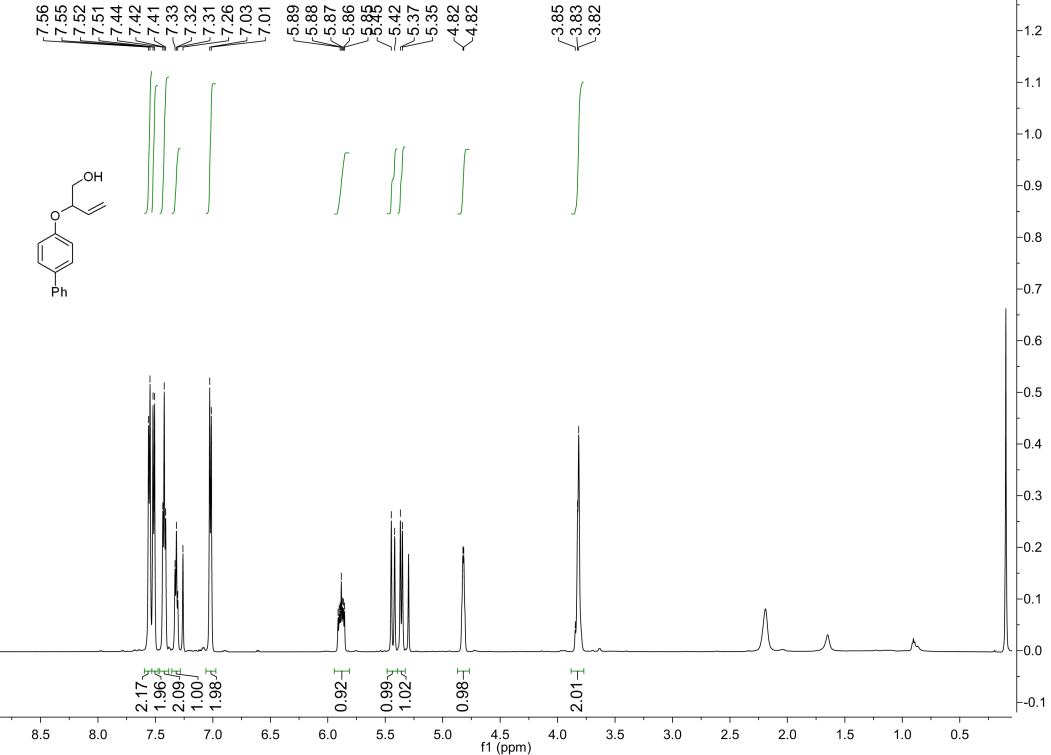
**

**
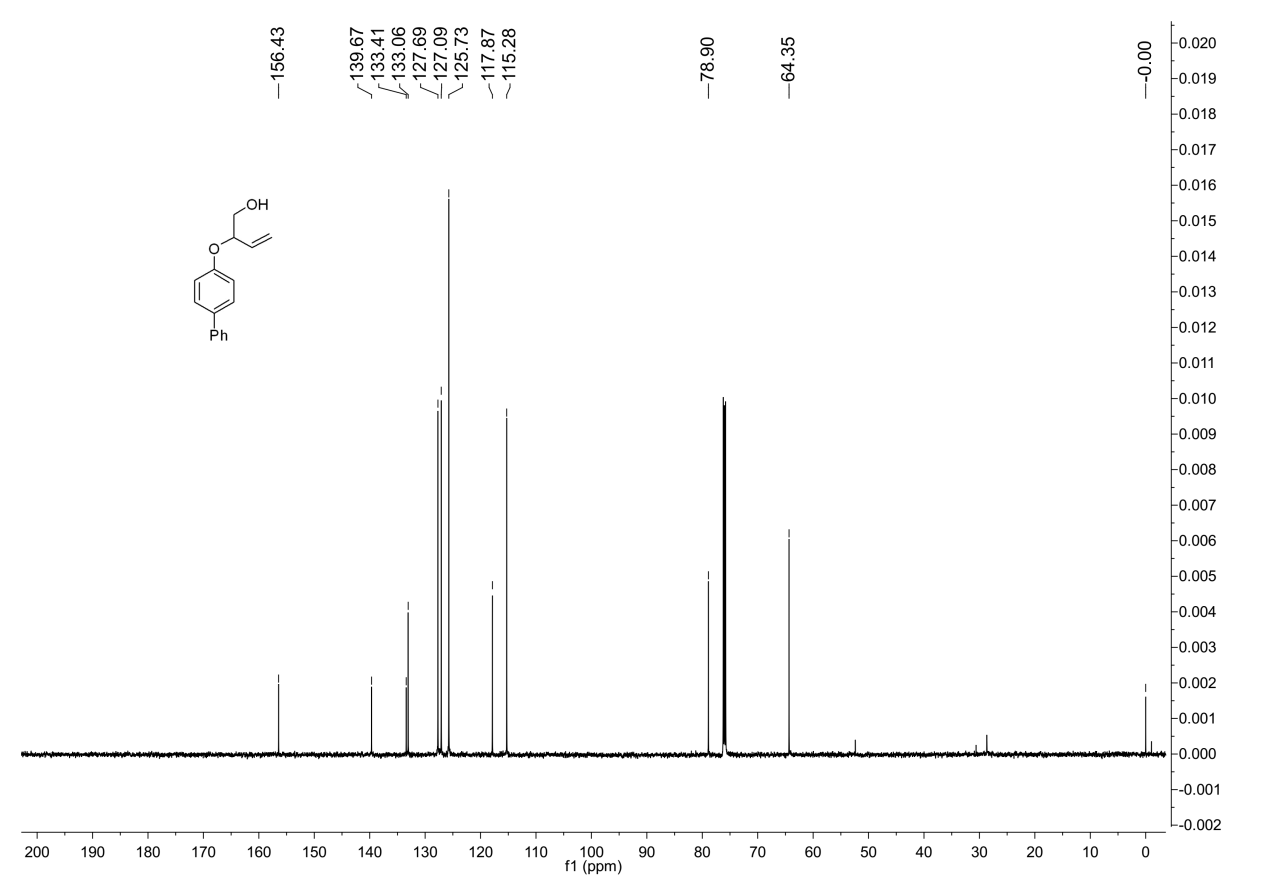
**

**
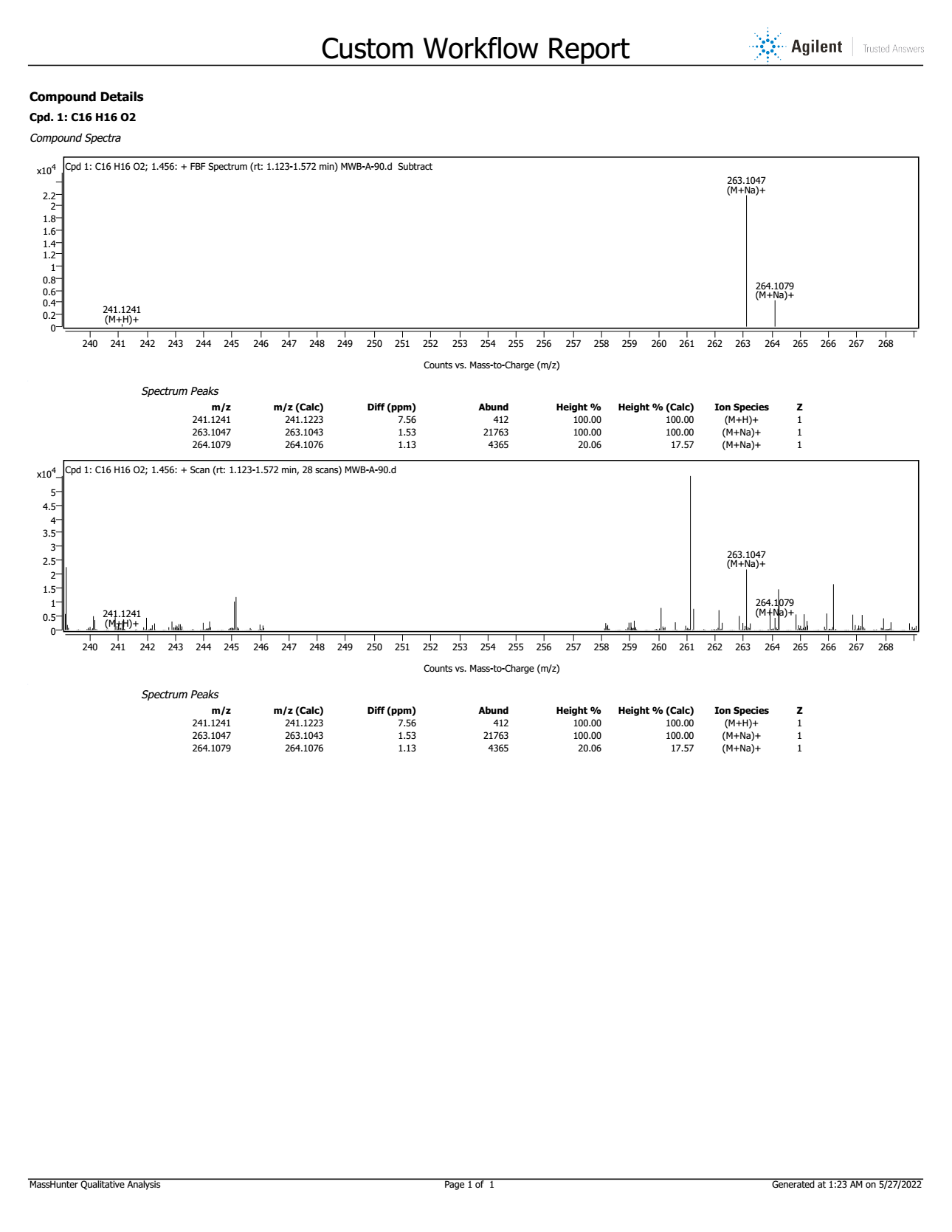
**

^1^H, ^13^C NMR and HRMS Spectra of Compound **3o**

**
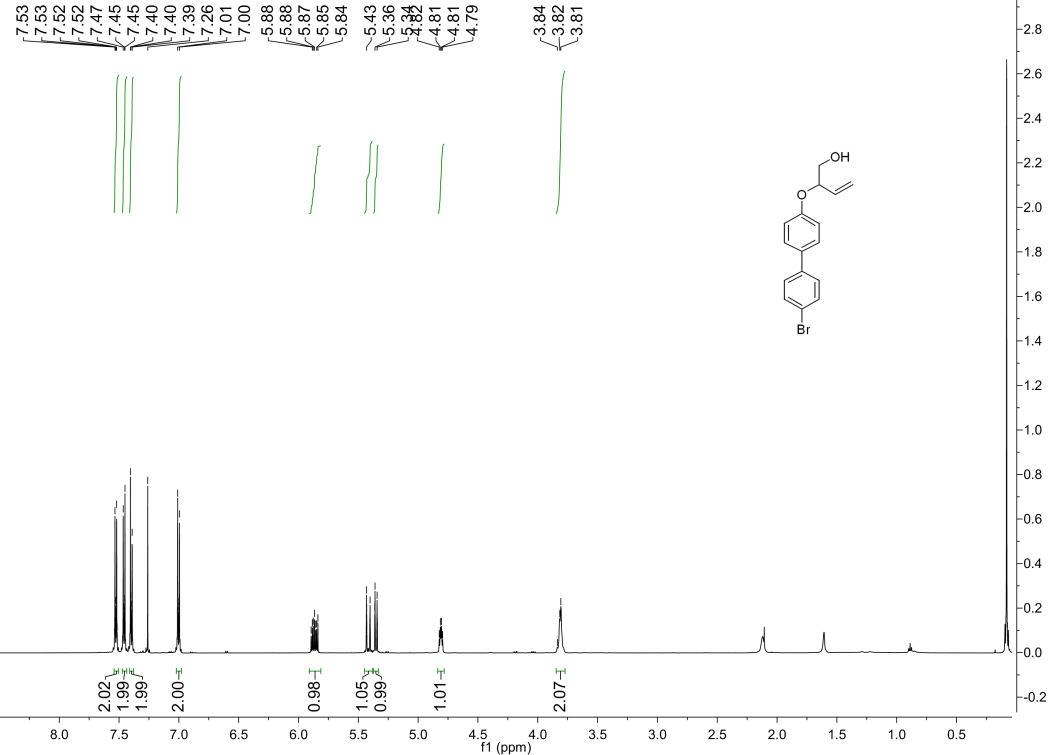
**

**
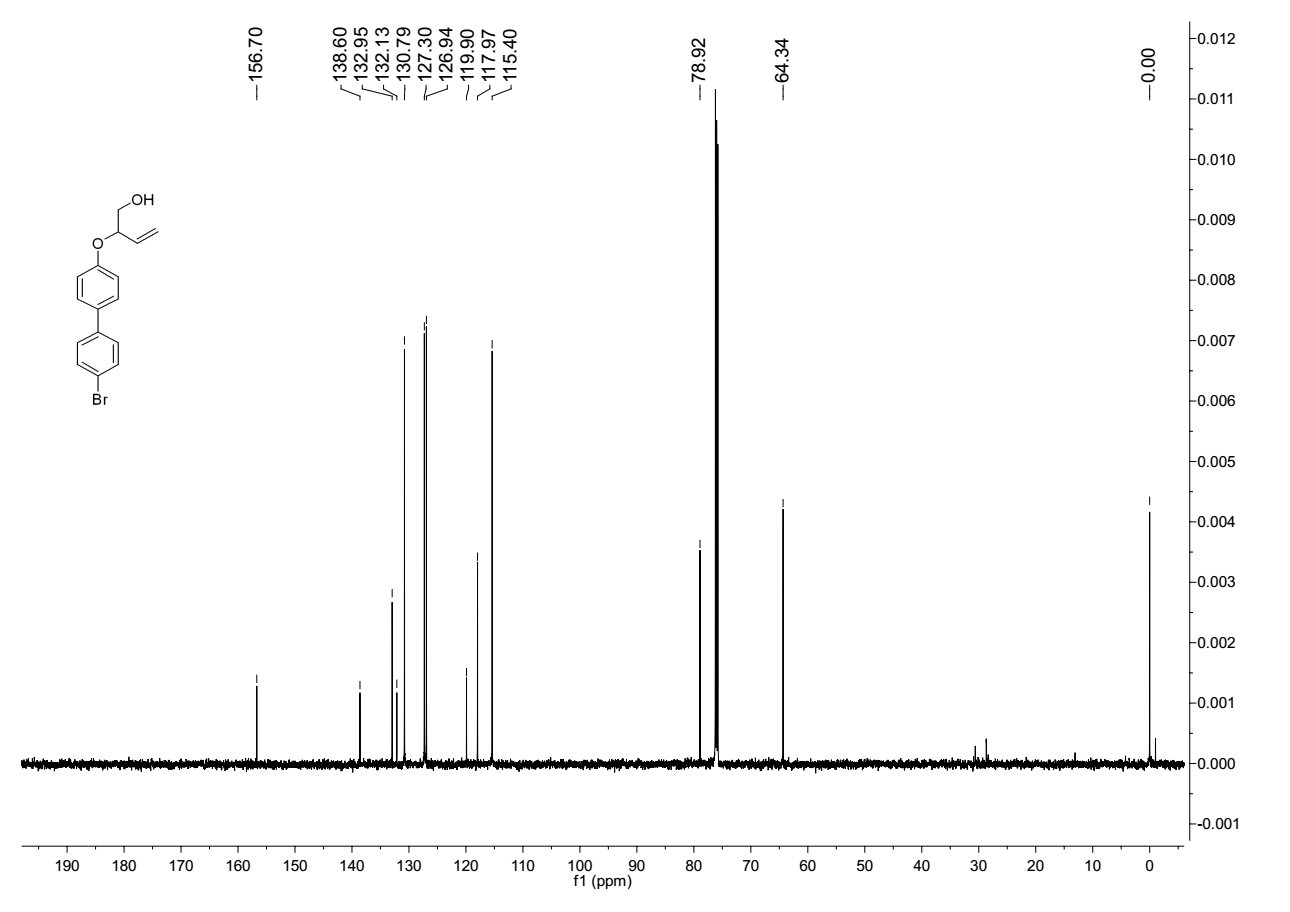
**

**
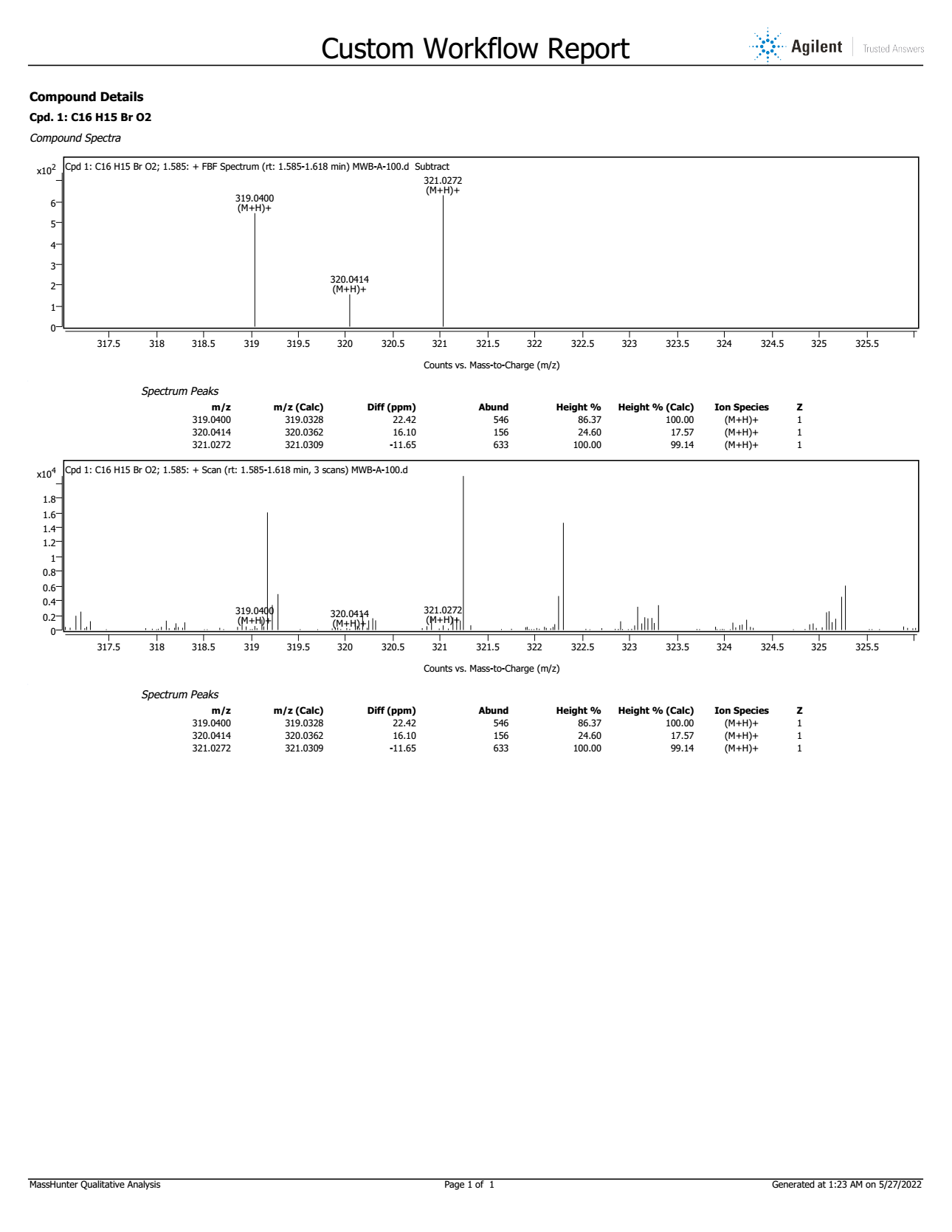
**

^1^H, ^13^C NMR and HRMS Spectra of Compound **3p**

**
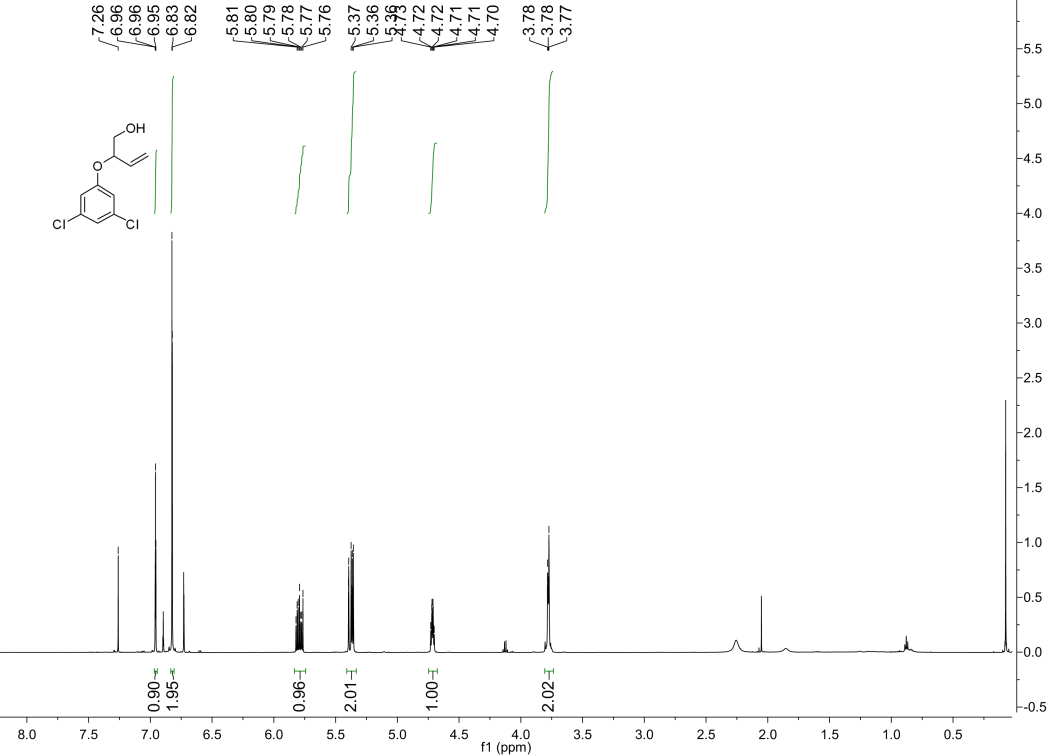
**

**
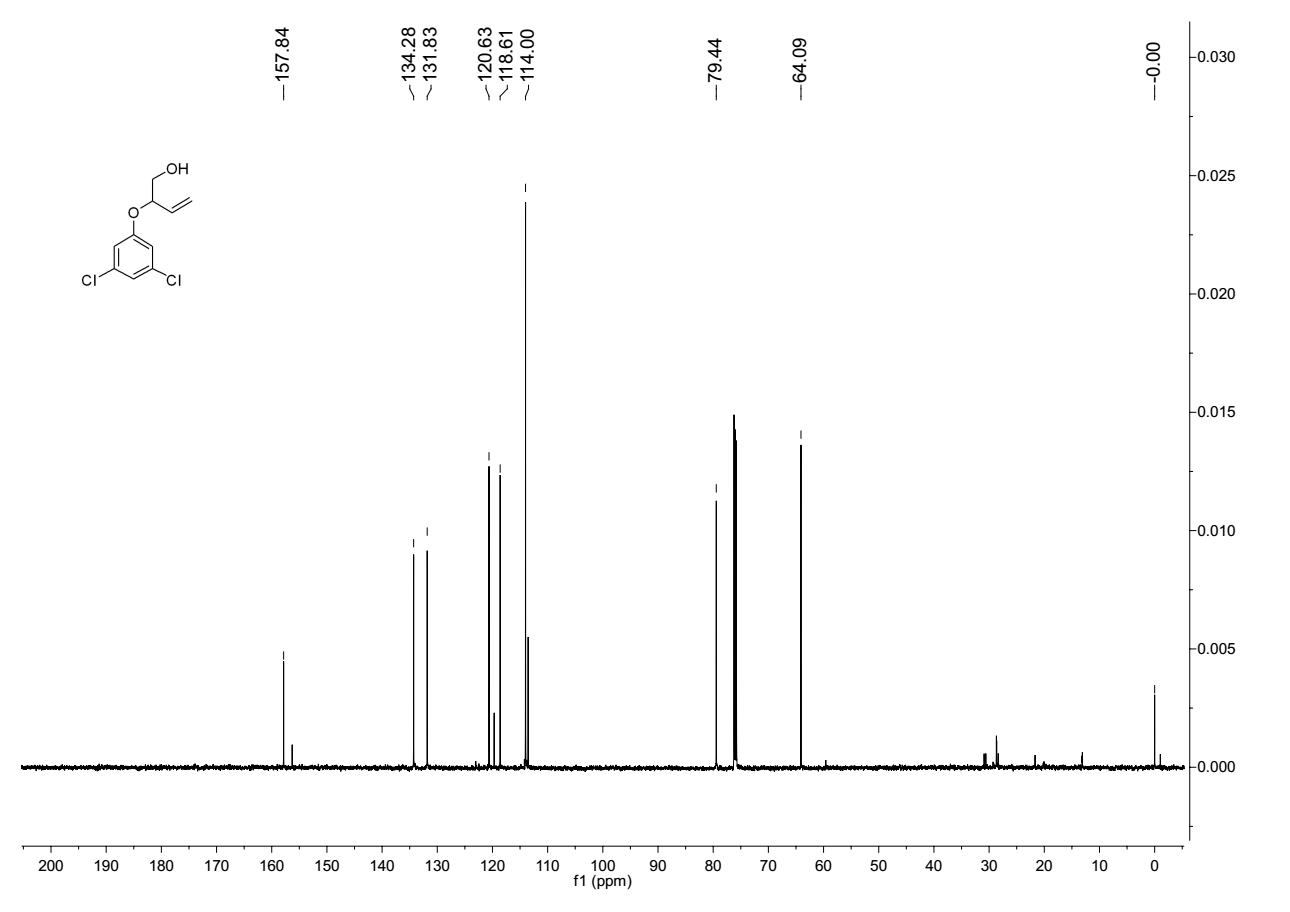
**

**
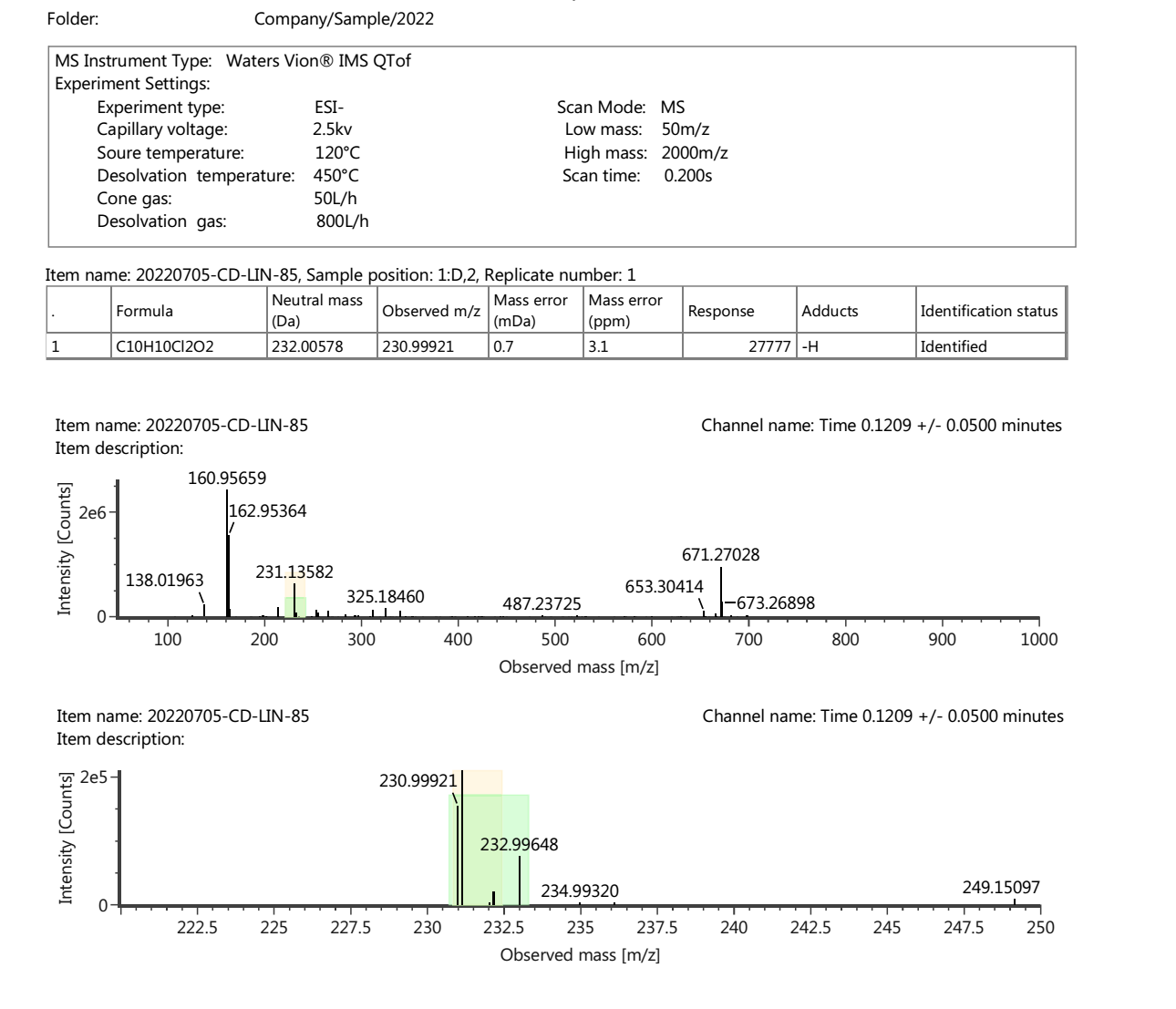
**

^1^H, ^13^C, ^19^F NMR and HRMS Spectra of Compound **3q**

**
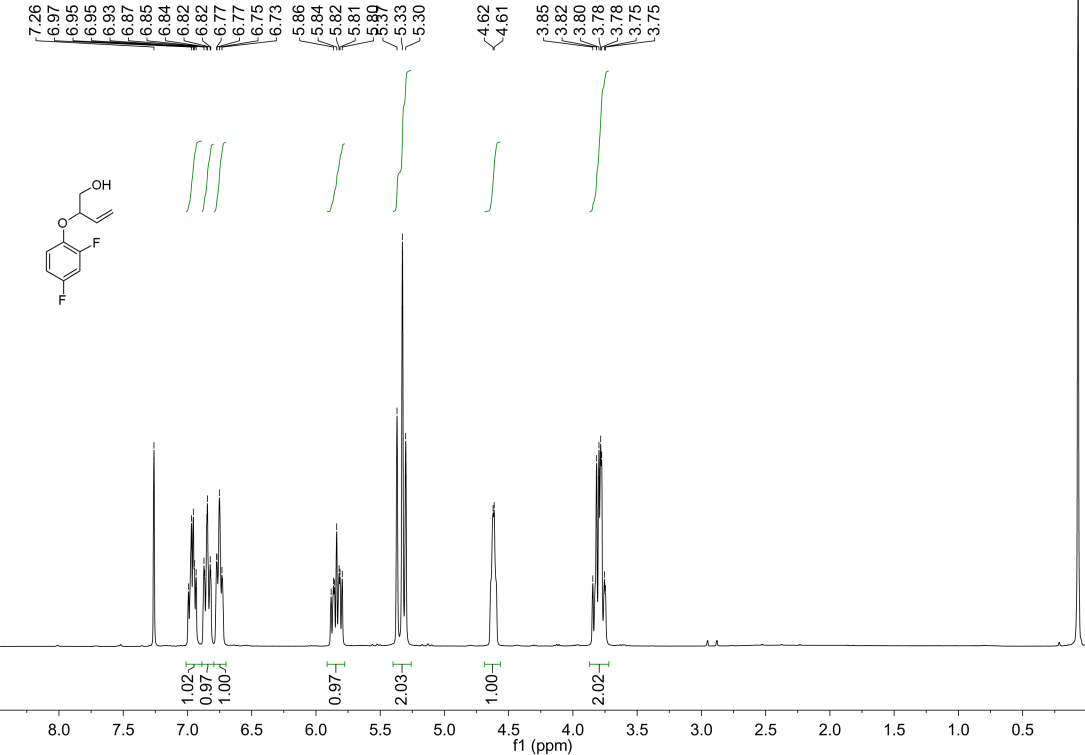
**

**
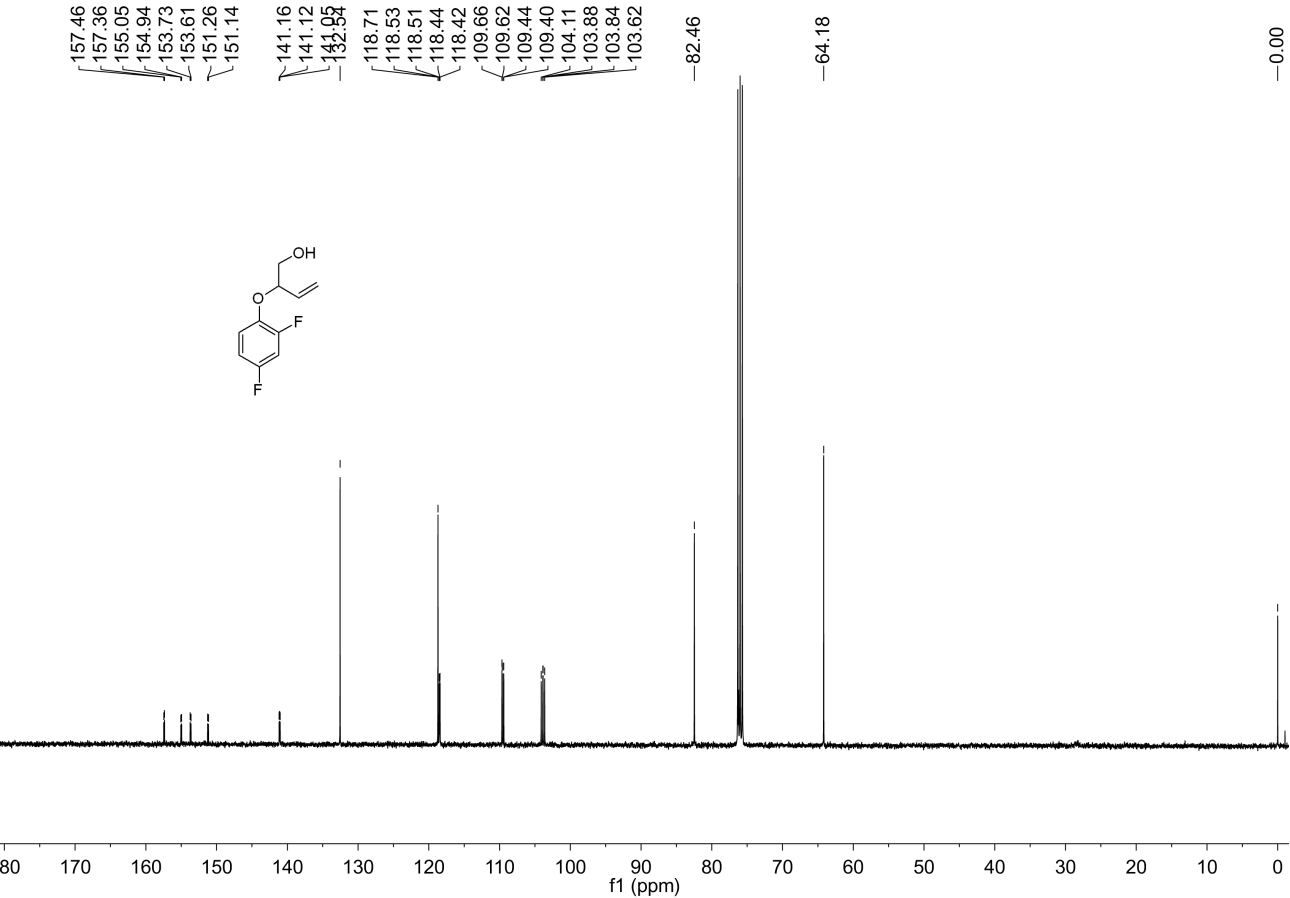
**

**
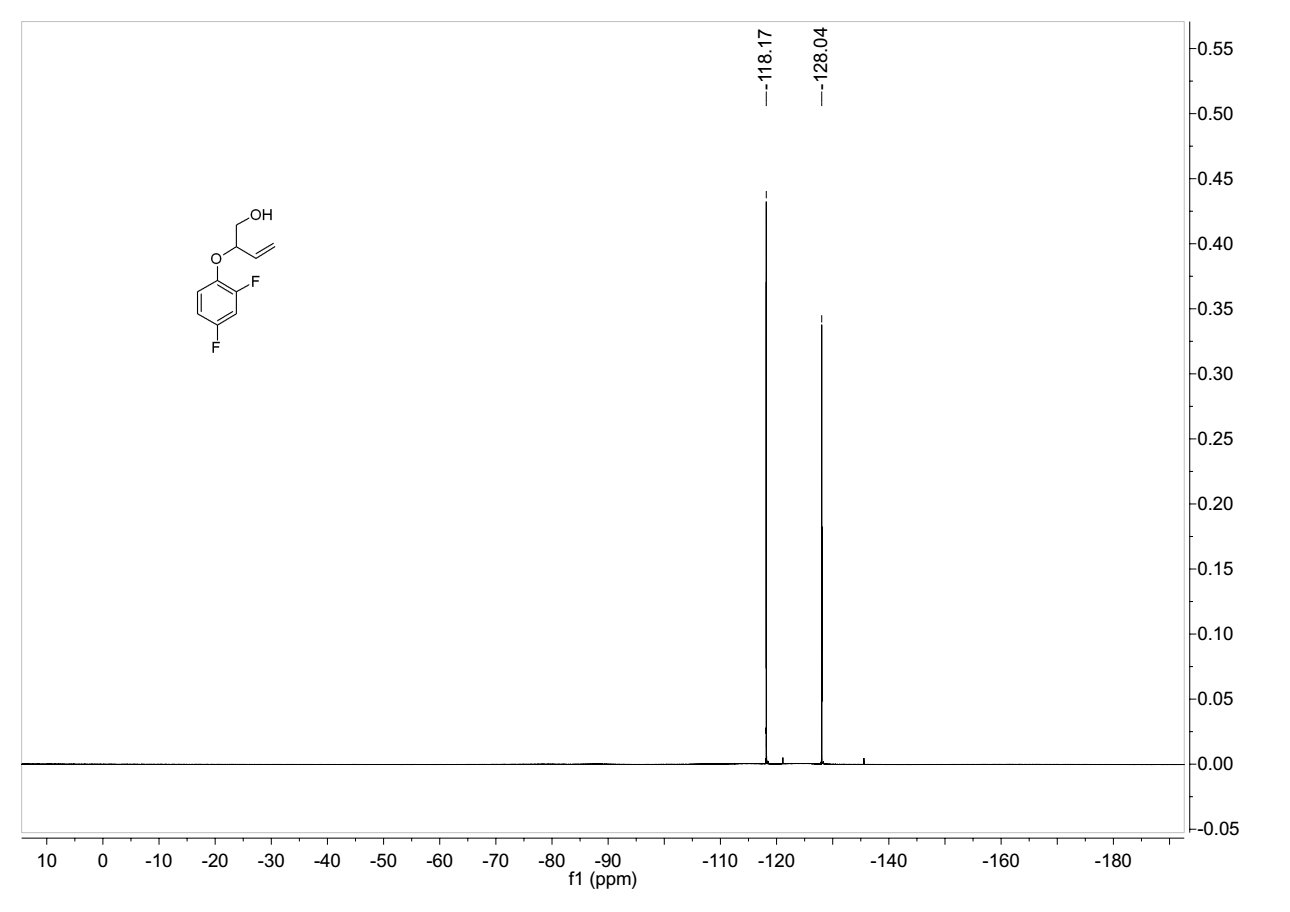
**

**
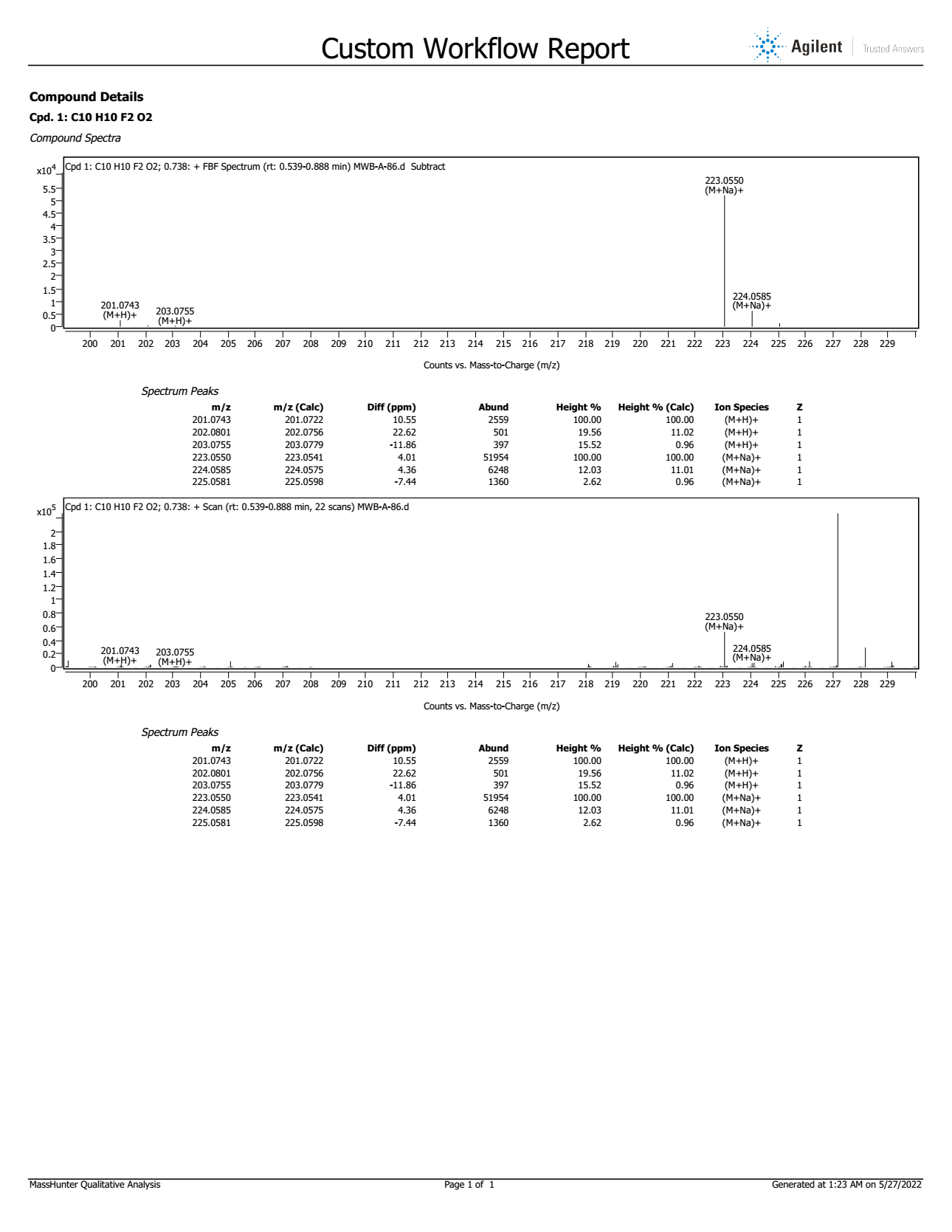
**

^1^H, ^13^C NMR and HRMS Spectra of Compound **3r**

**
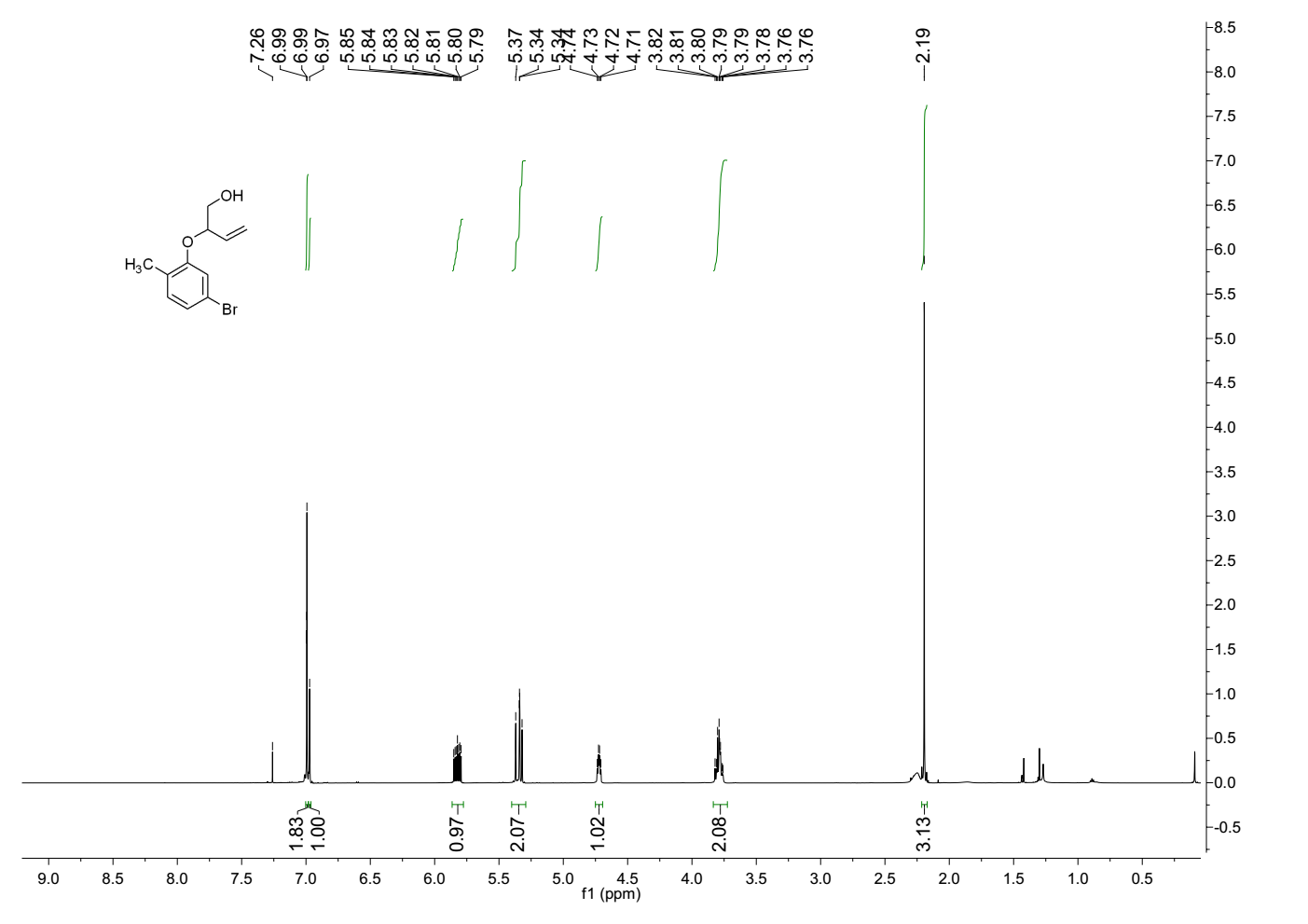
**

**
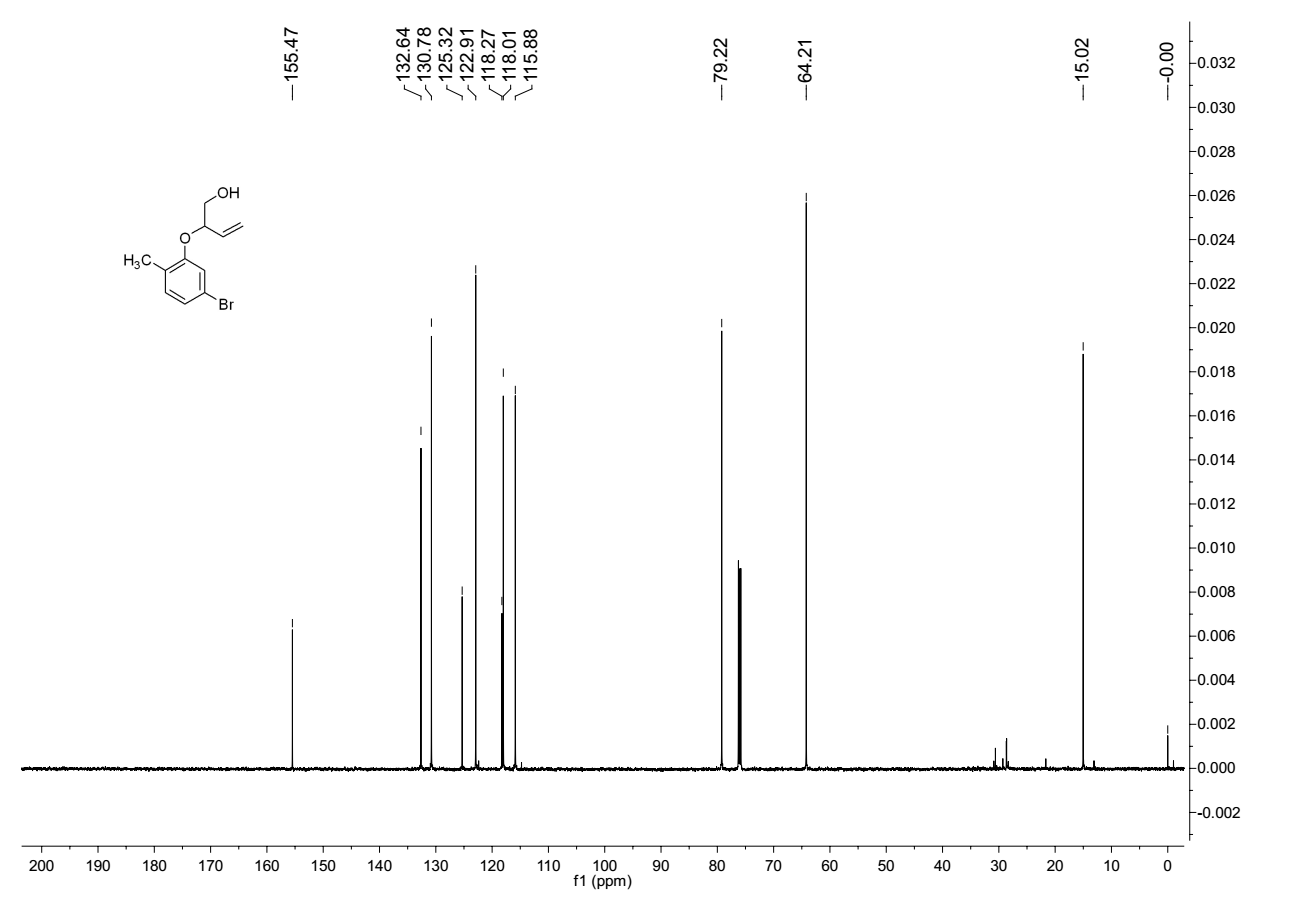
**

**
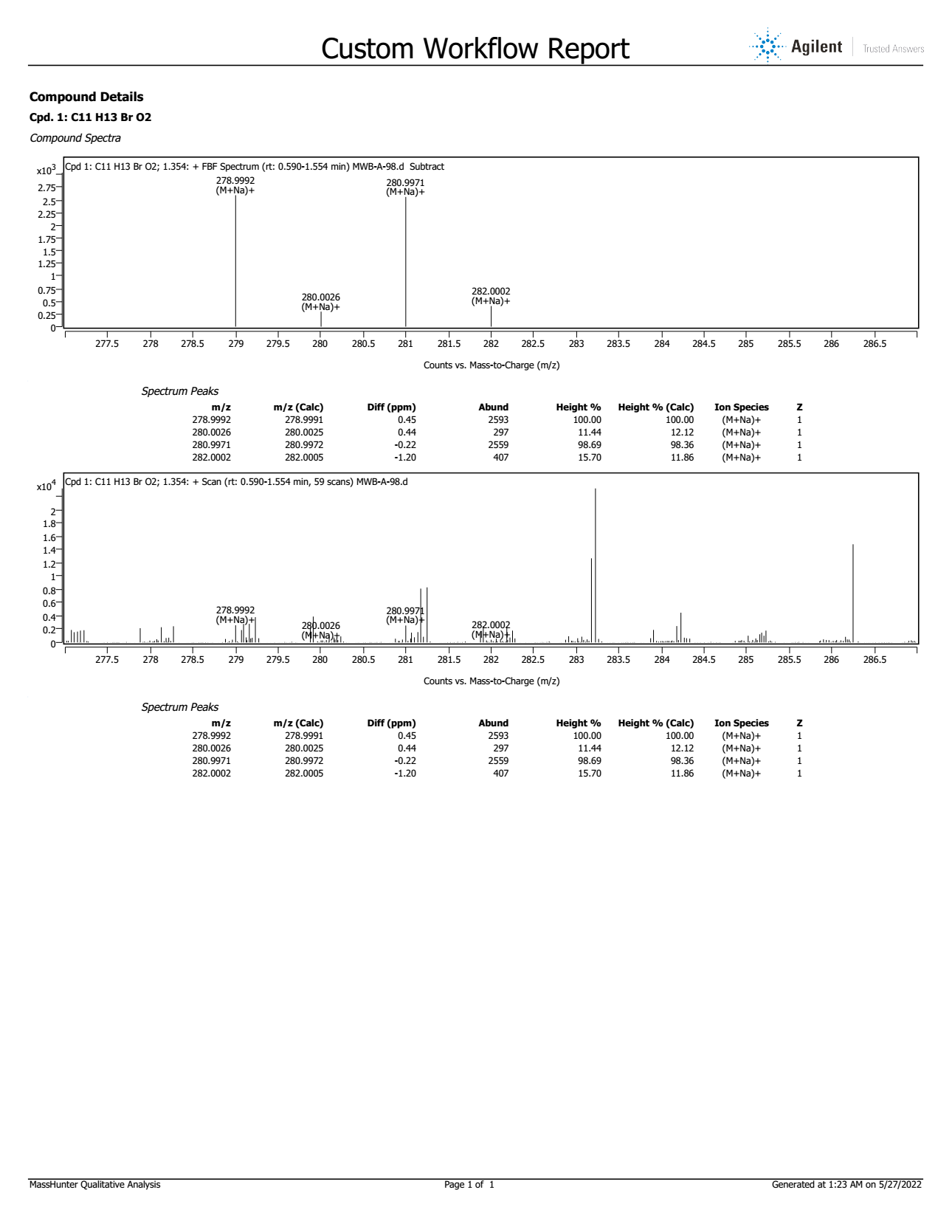
**

^1^H, ^13^C NMR and HRMS Spectra of Compound **3s**

**
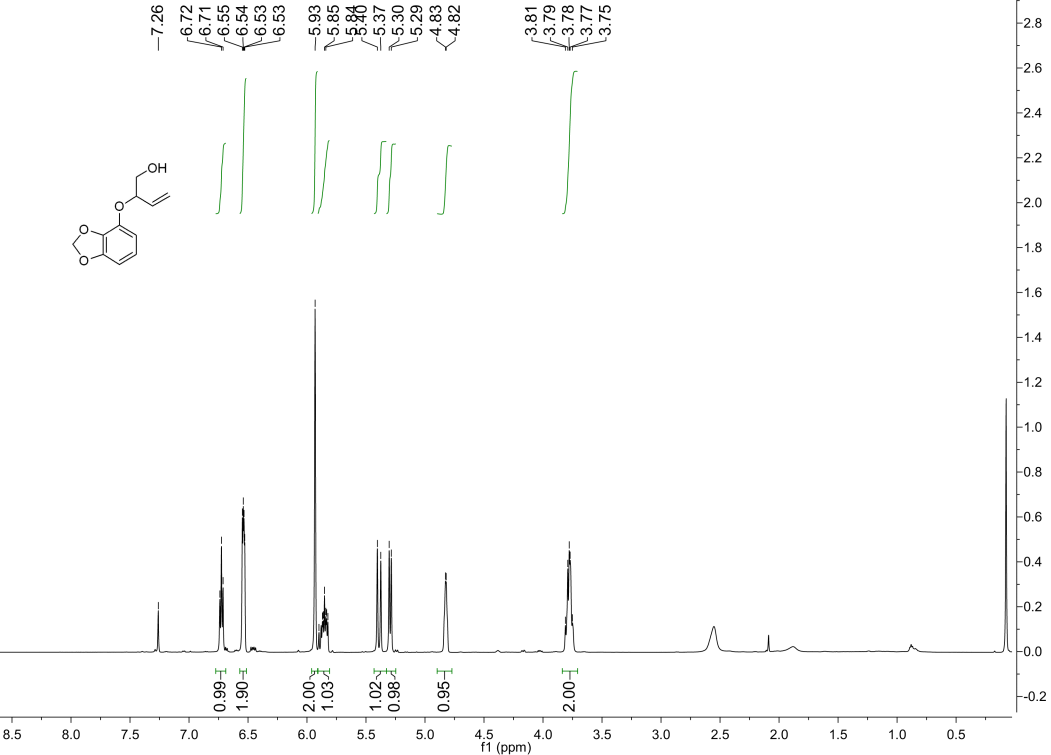
**

**
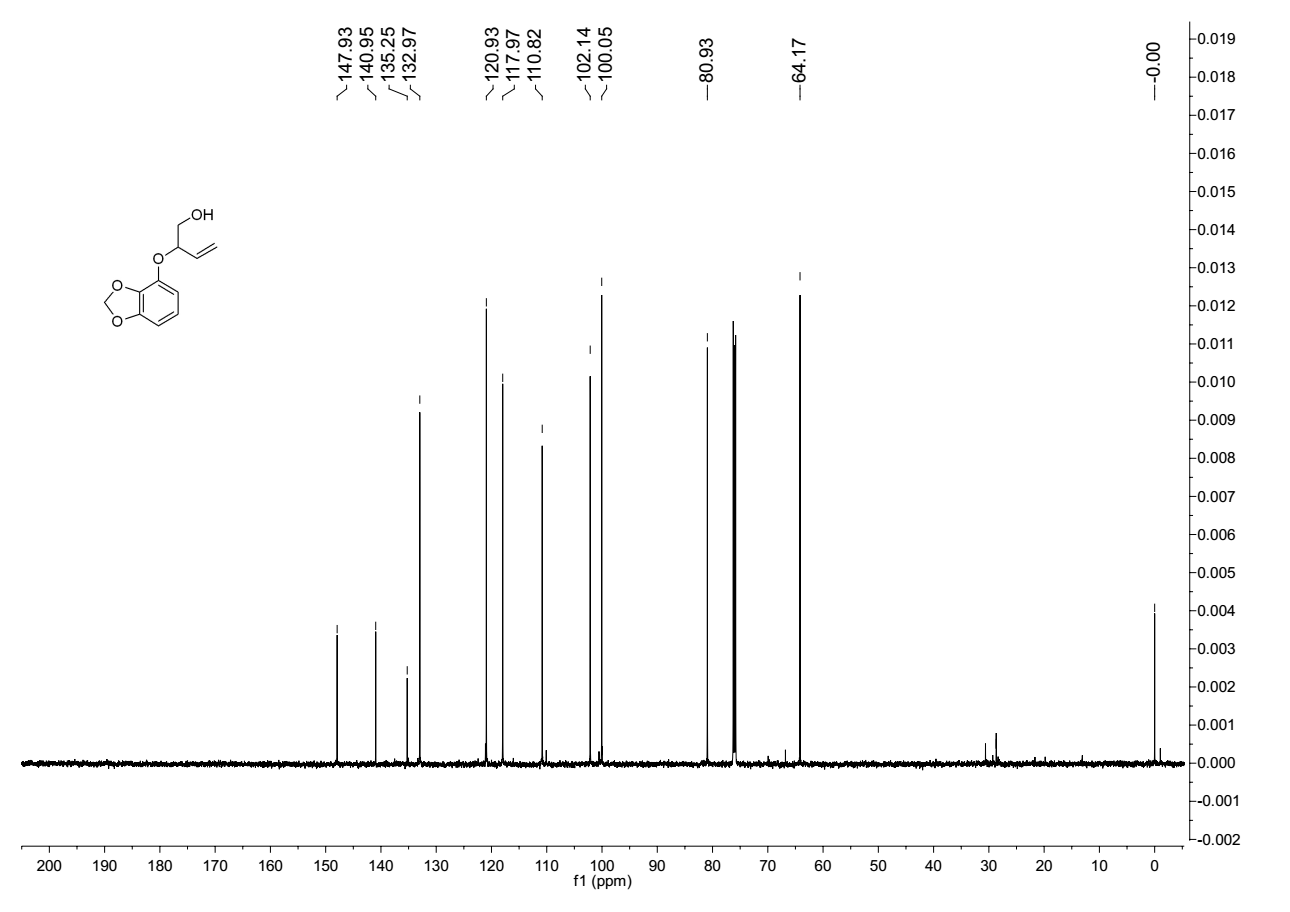
**

**
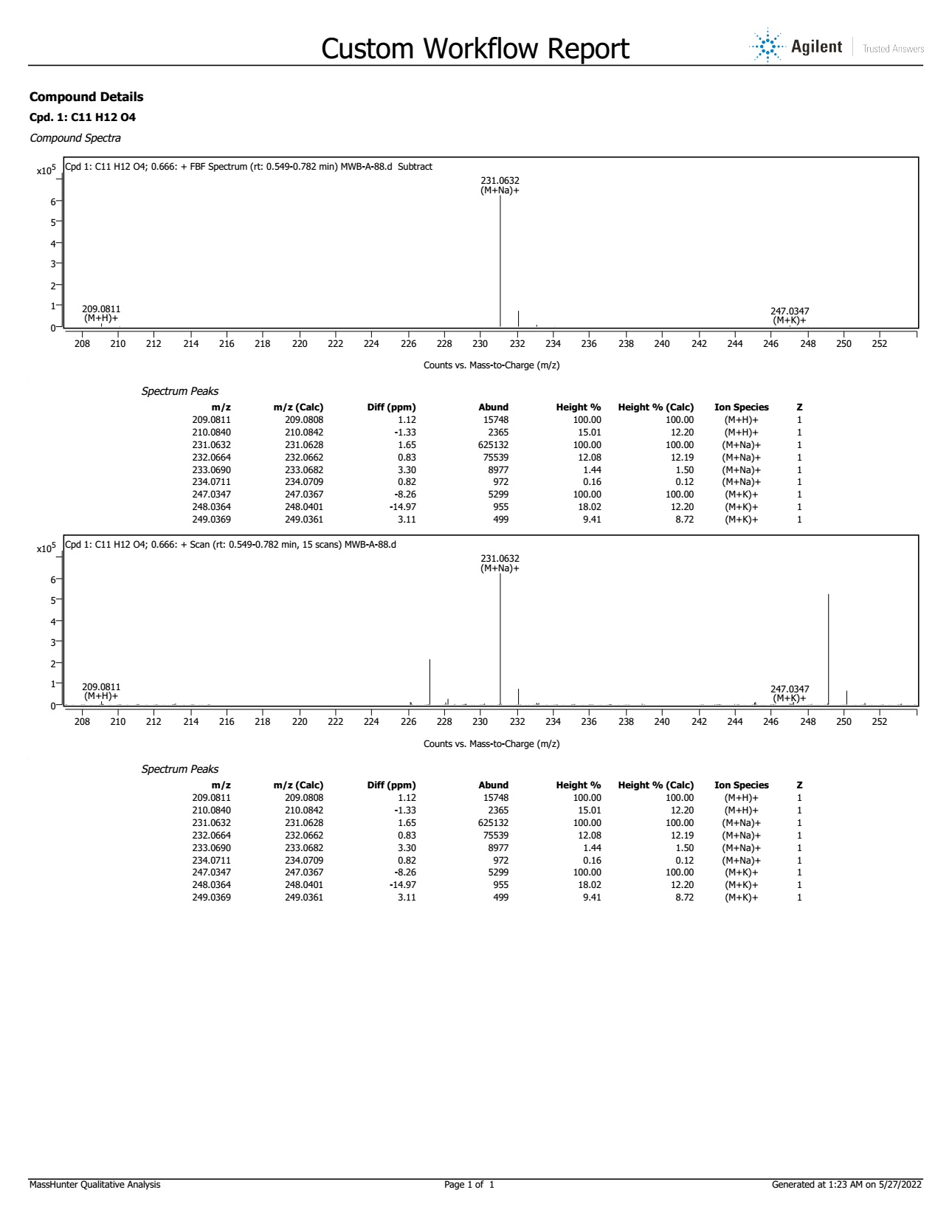
**

^1^H, ^13^C NMR and HRMS Spectra of Compound **3t**

**
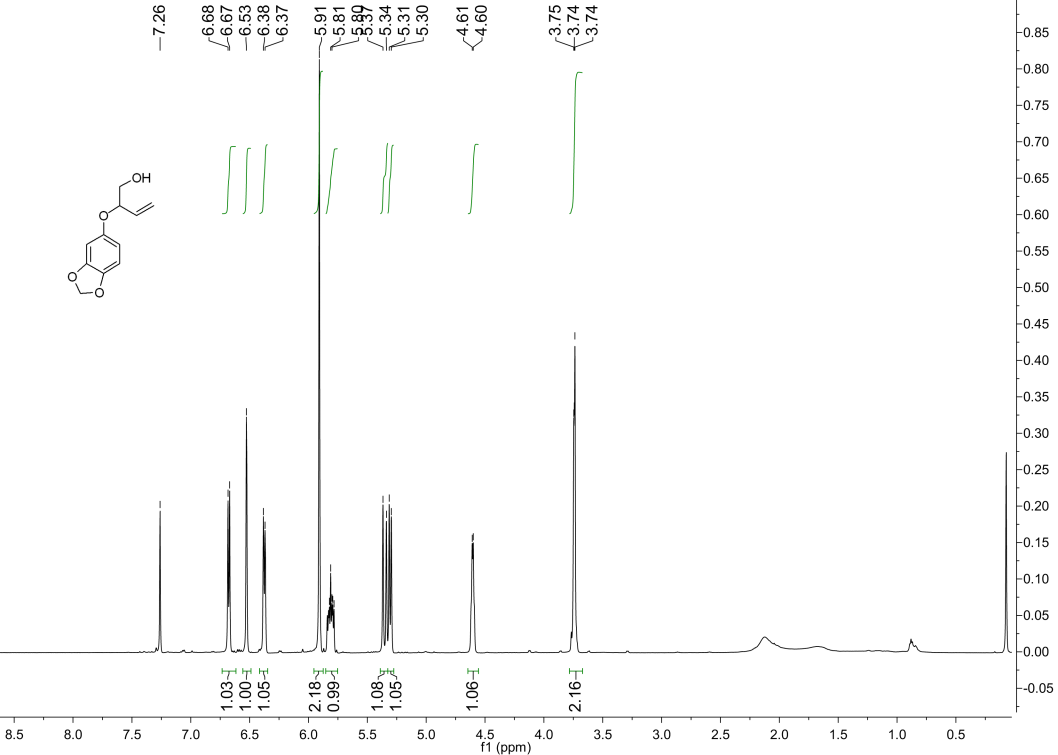
**

**
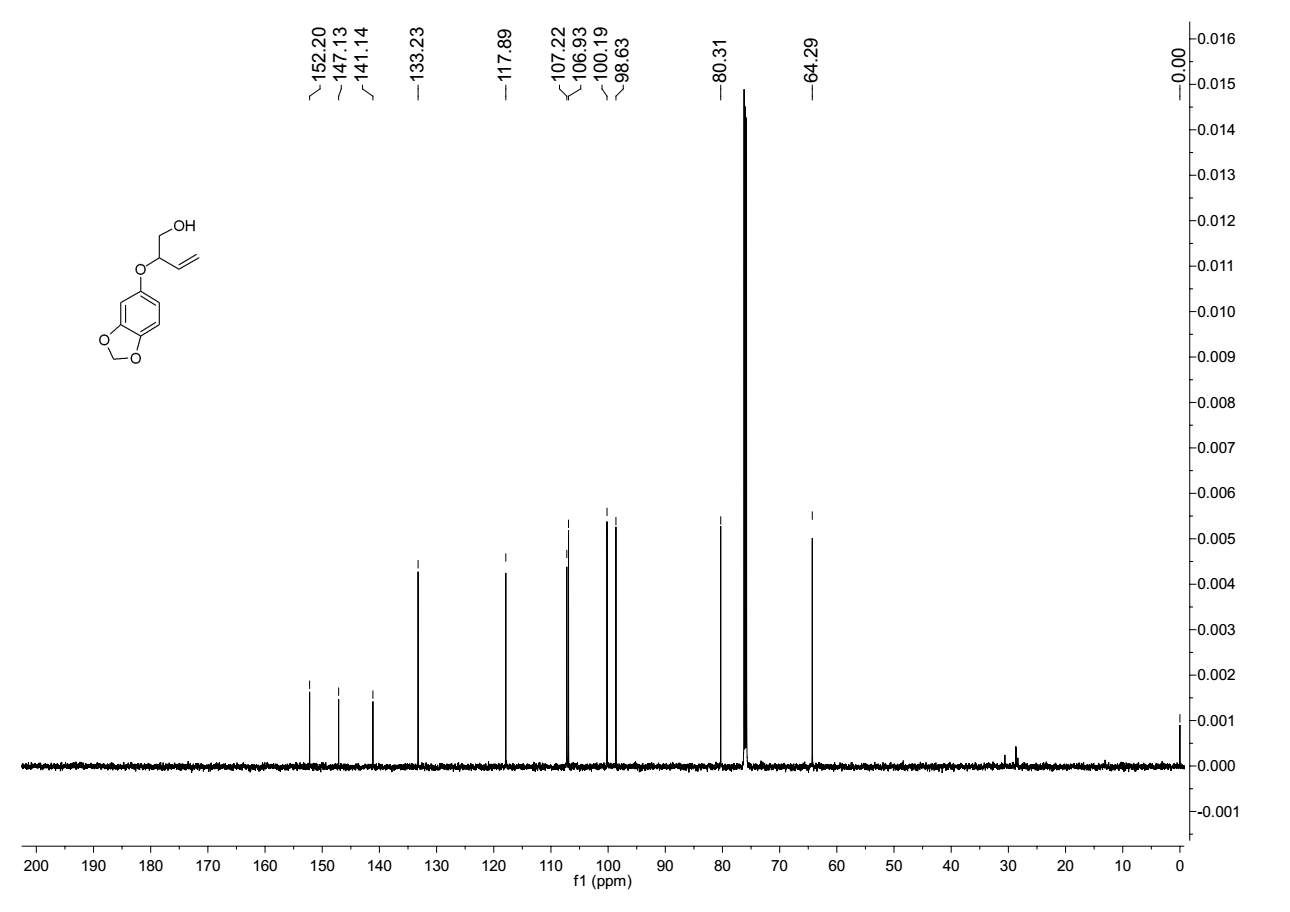
**

**
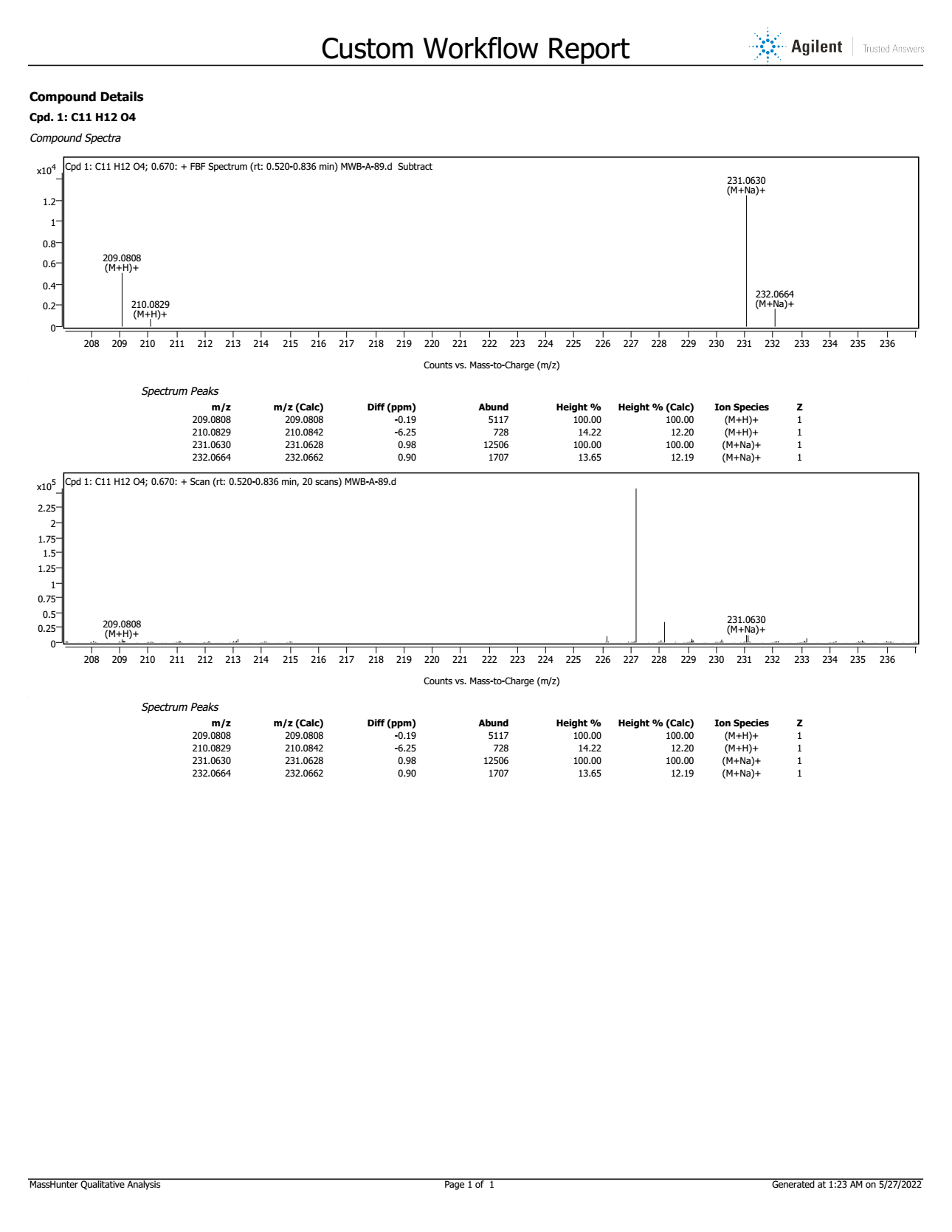
**

^1^H, ^13^C NMR and HRMS Spectra of Compound **3u**

**
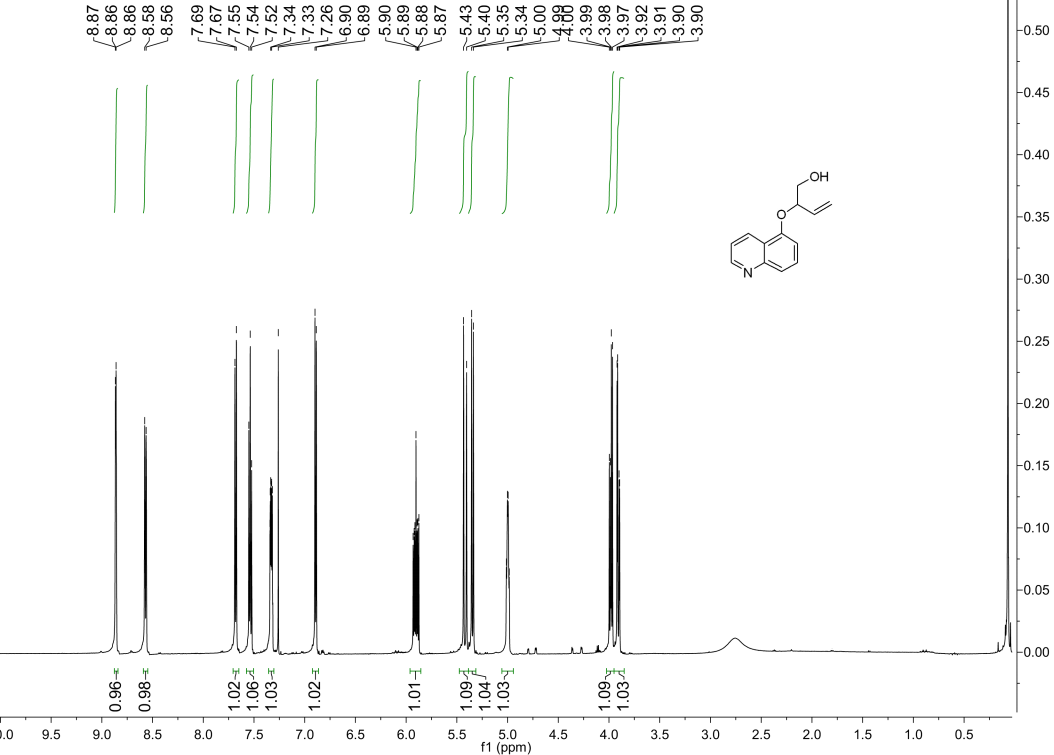
**

**
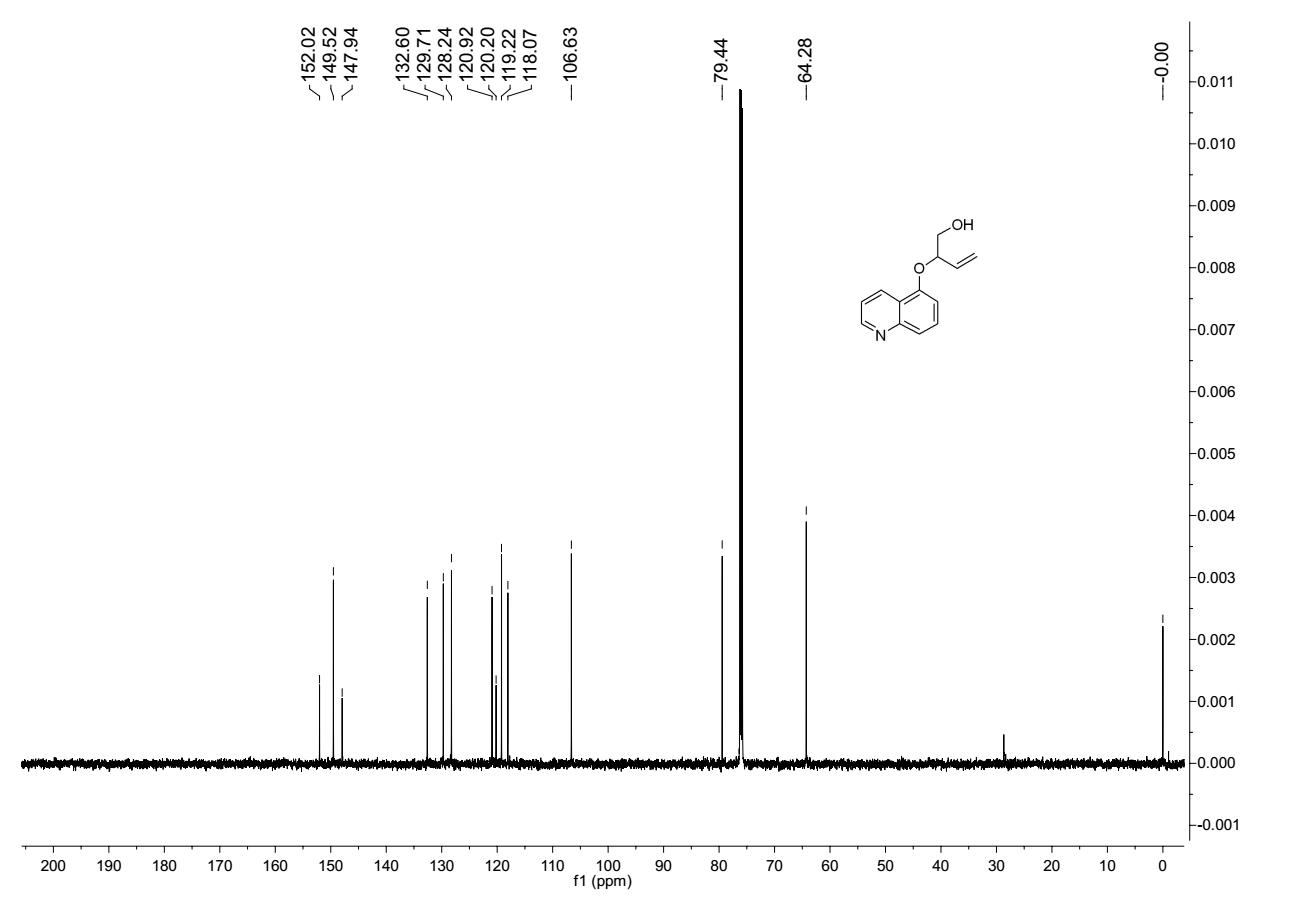
**

**
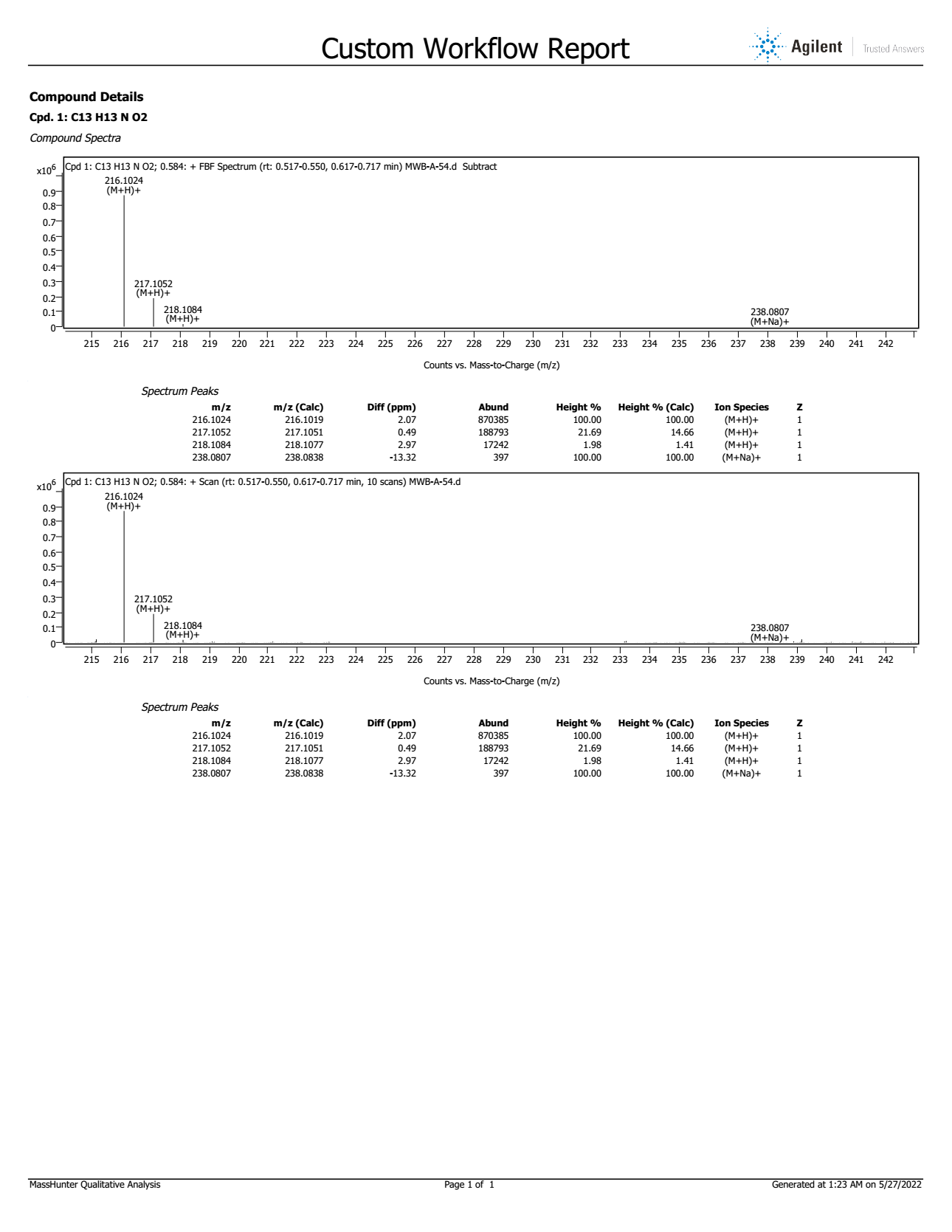
**

^1^H, ^13^C NMR and HRMS Spectra of Compound **3v**

**
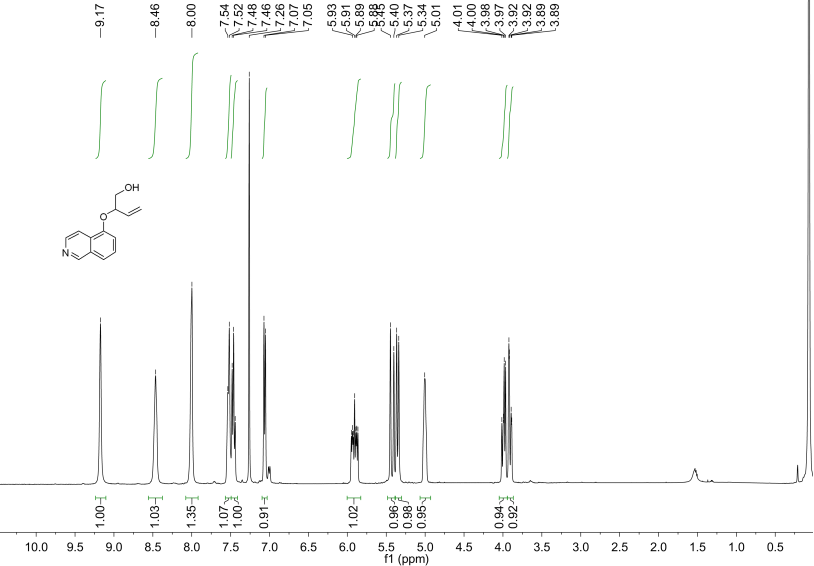
**

**
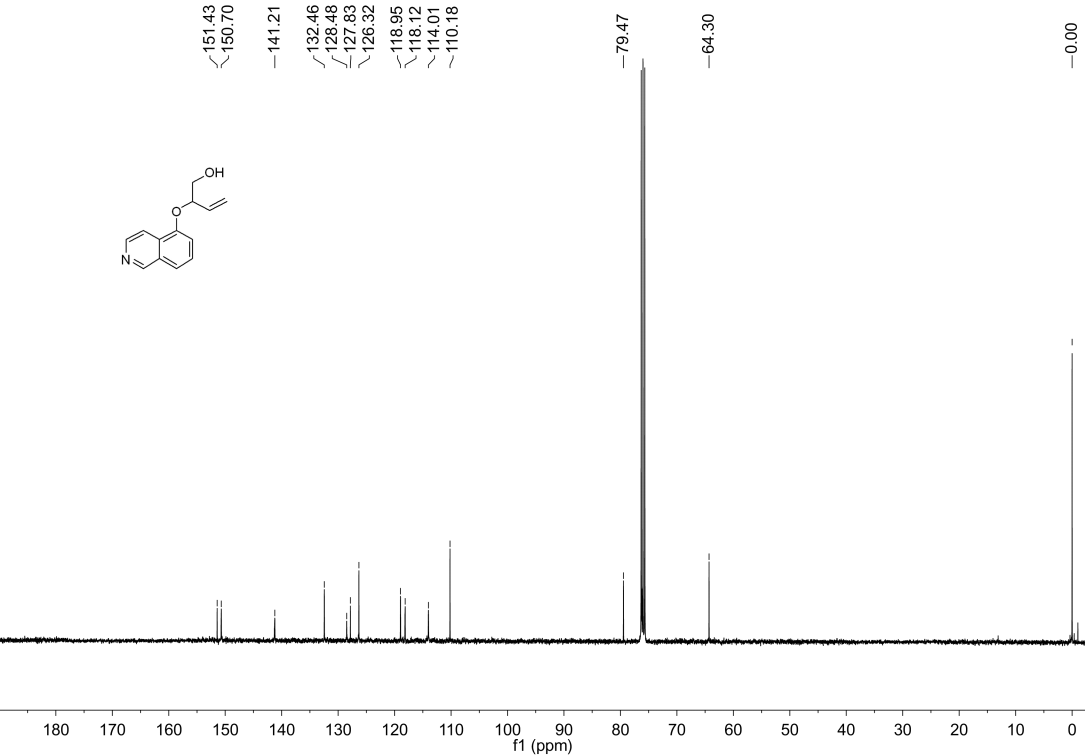
**

**
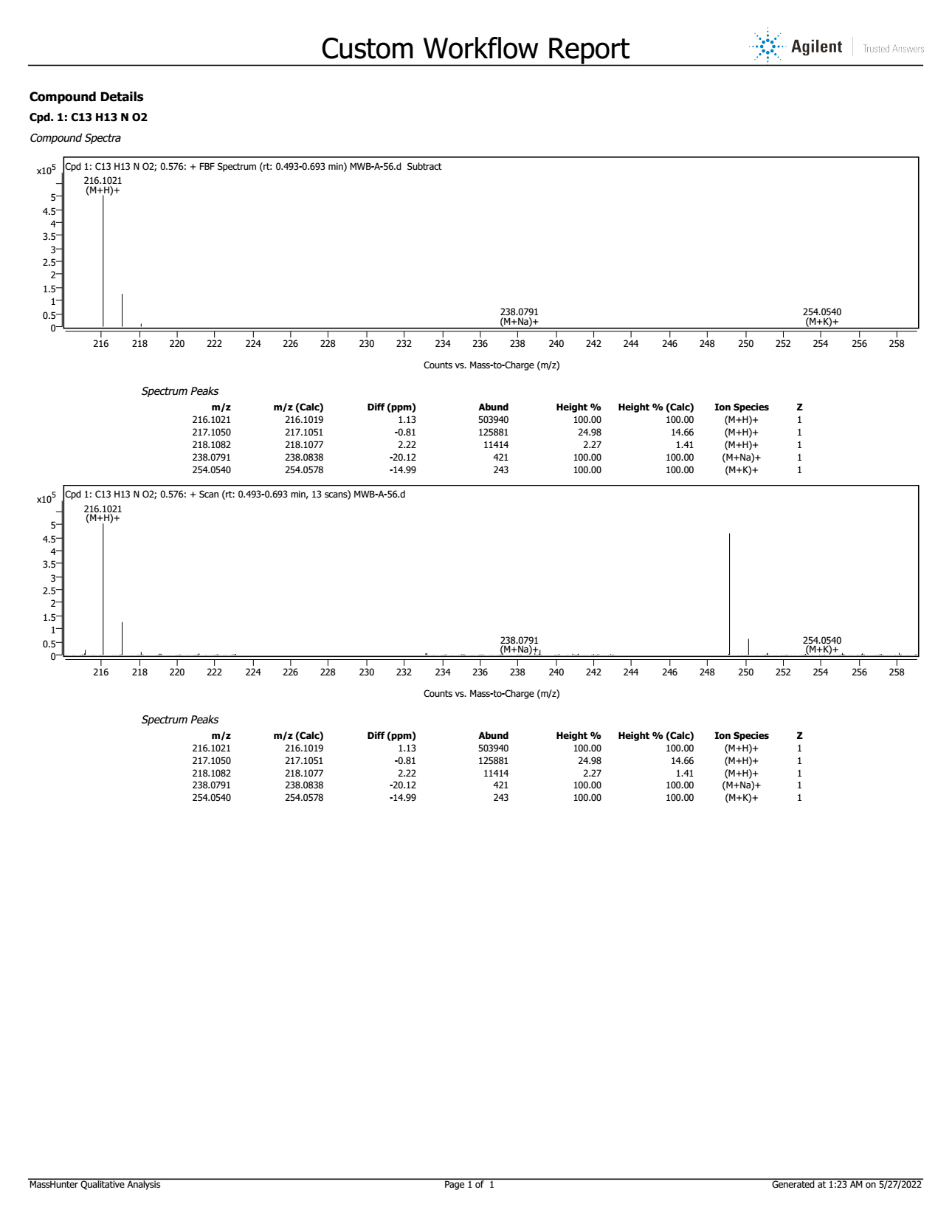
**

^1^H, ^13^C NMR and HRMS Spectra of Compound **3w**

**
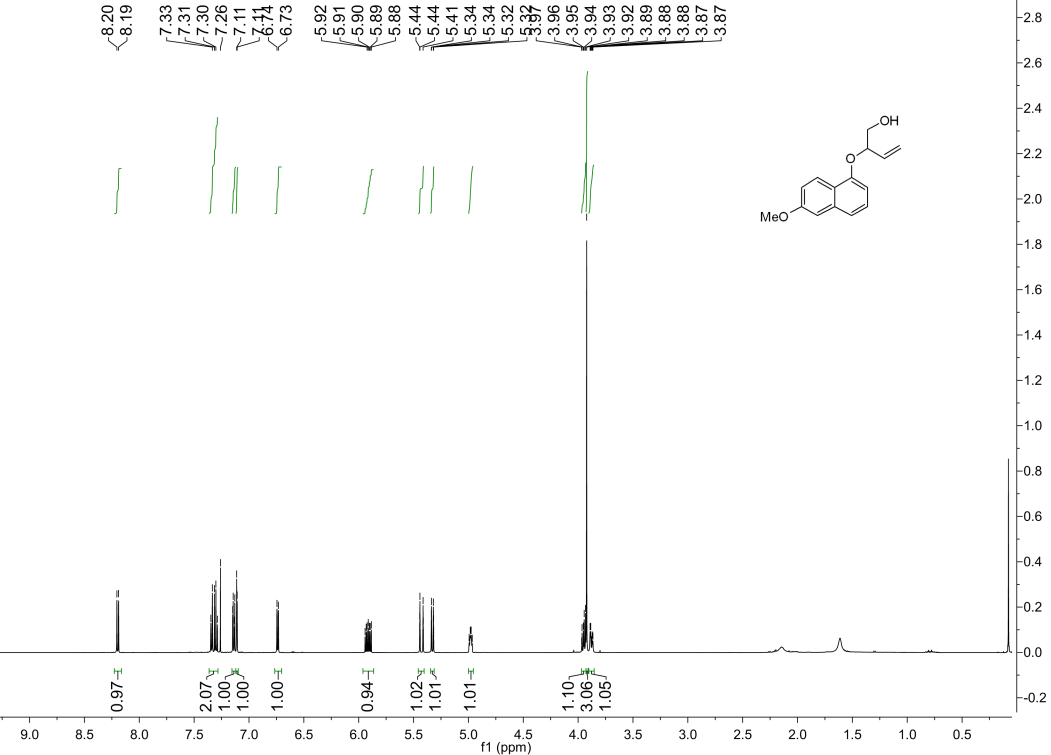
**

**
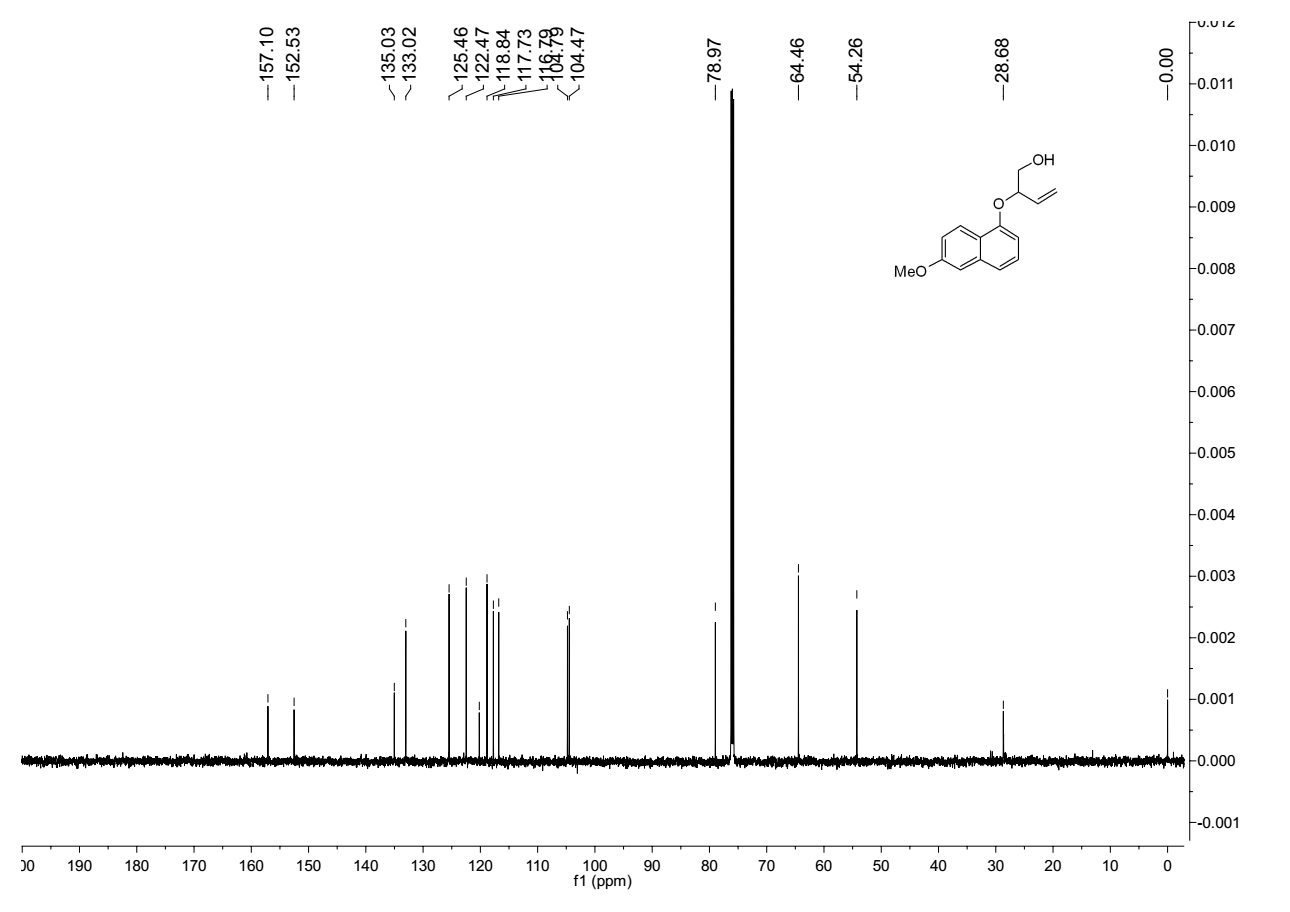
**

**
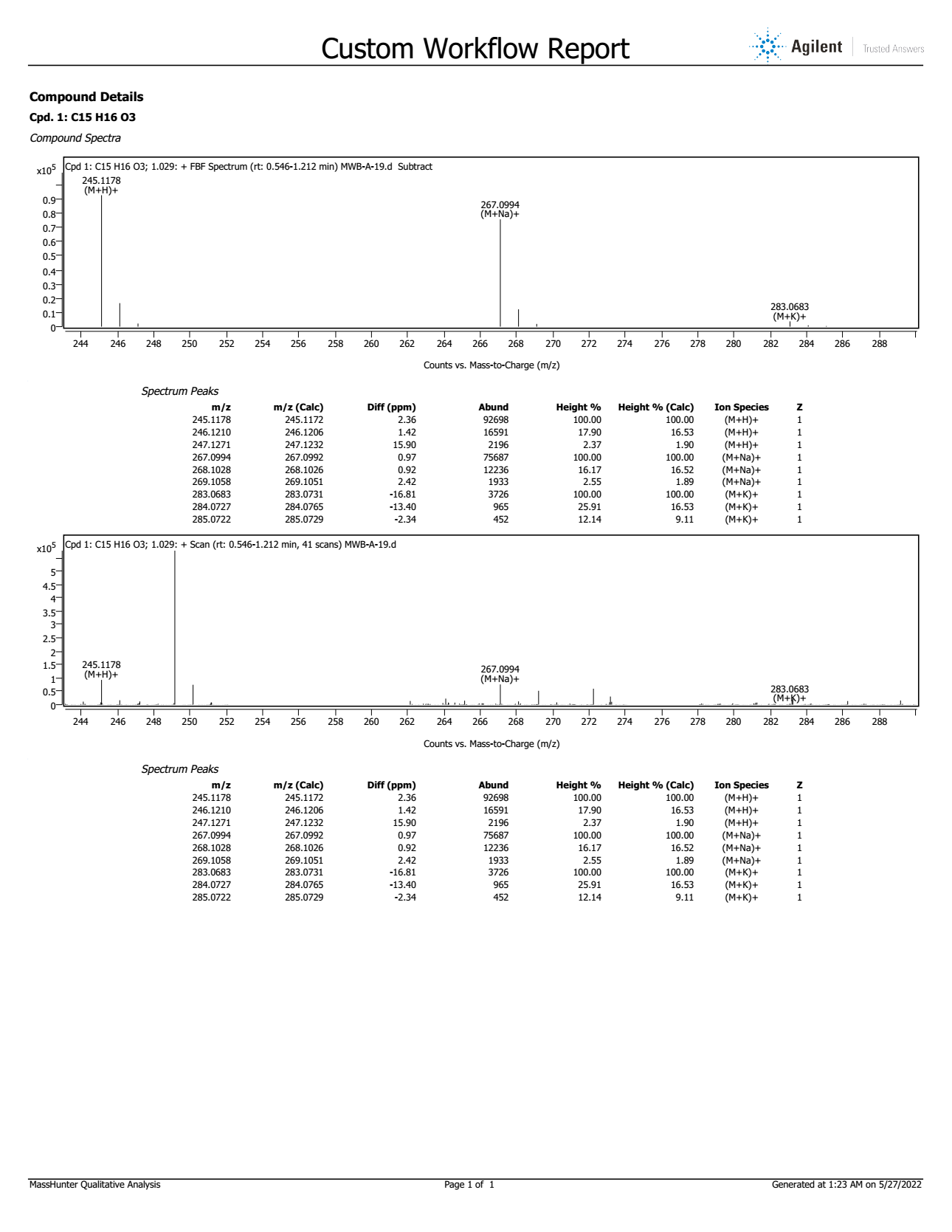
**

^1^H, ^13^C NMR and HRMS Spectra of Compound **3x**

**
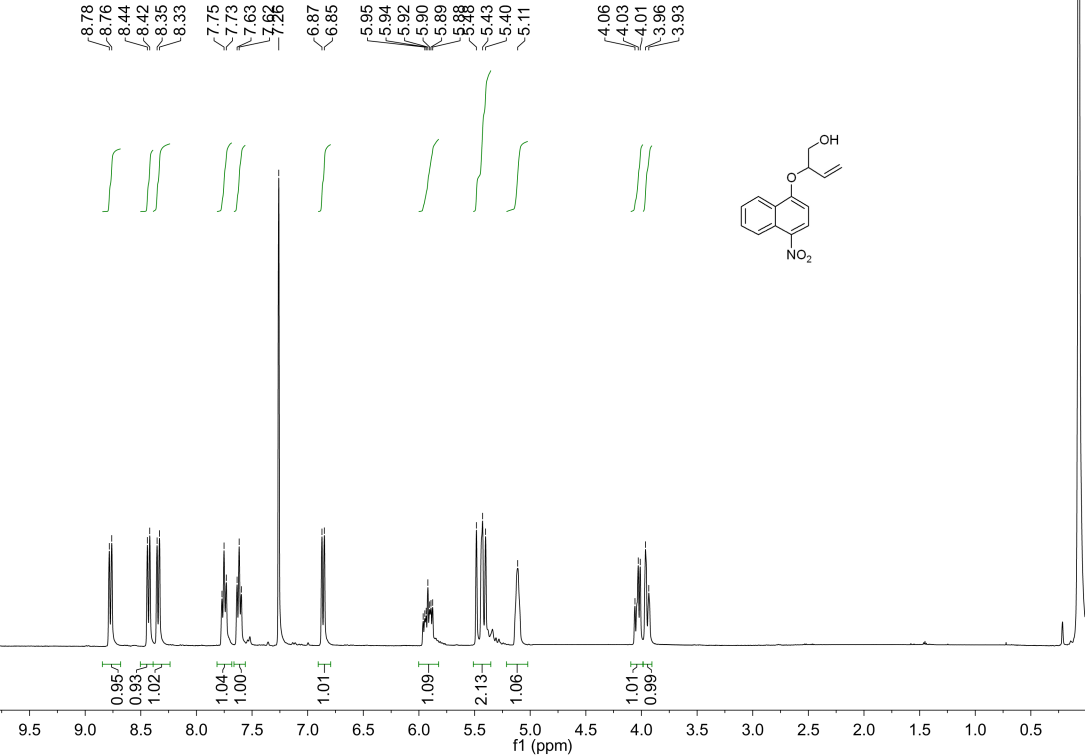
**

**
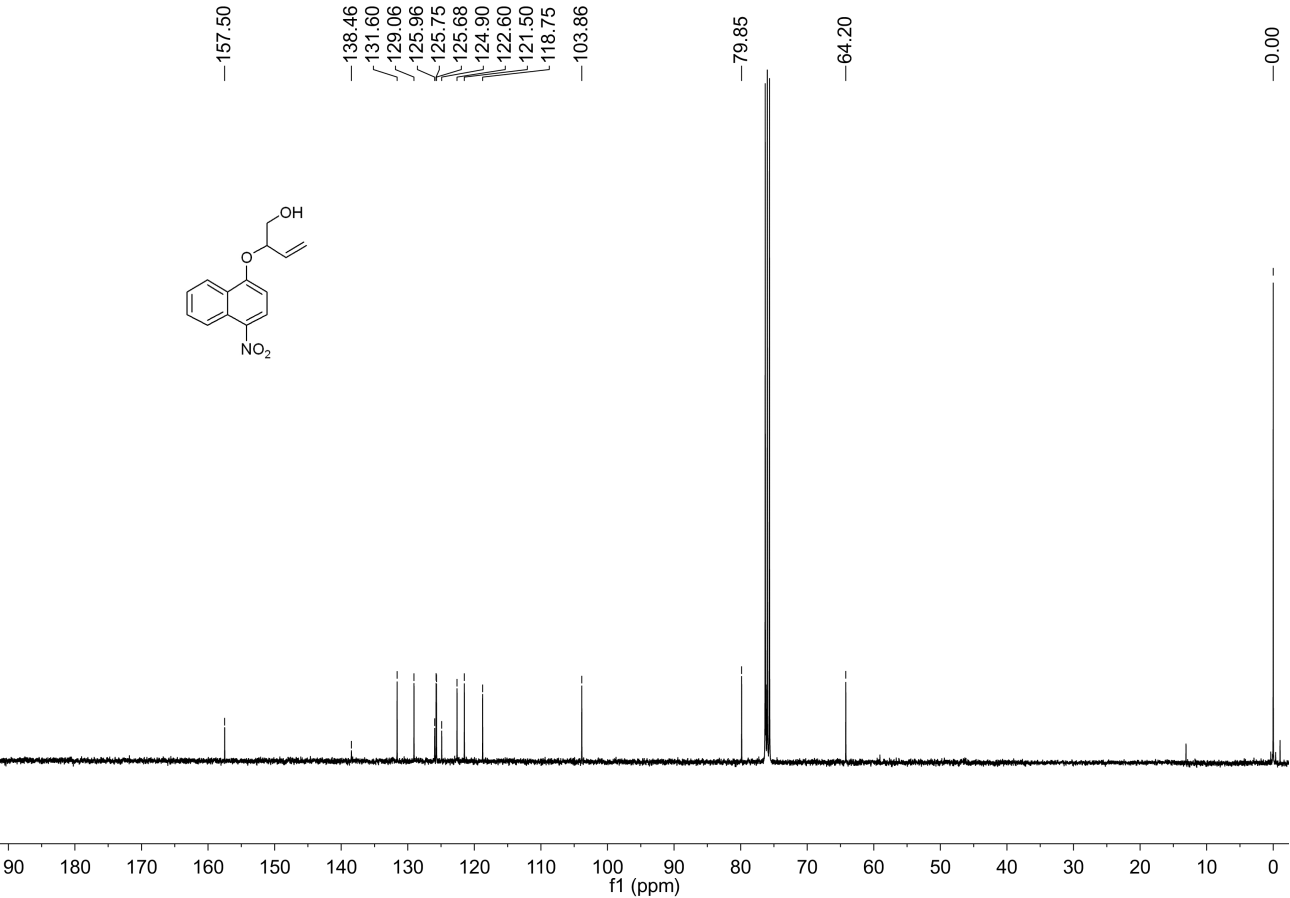
**

**
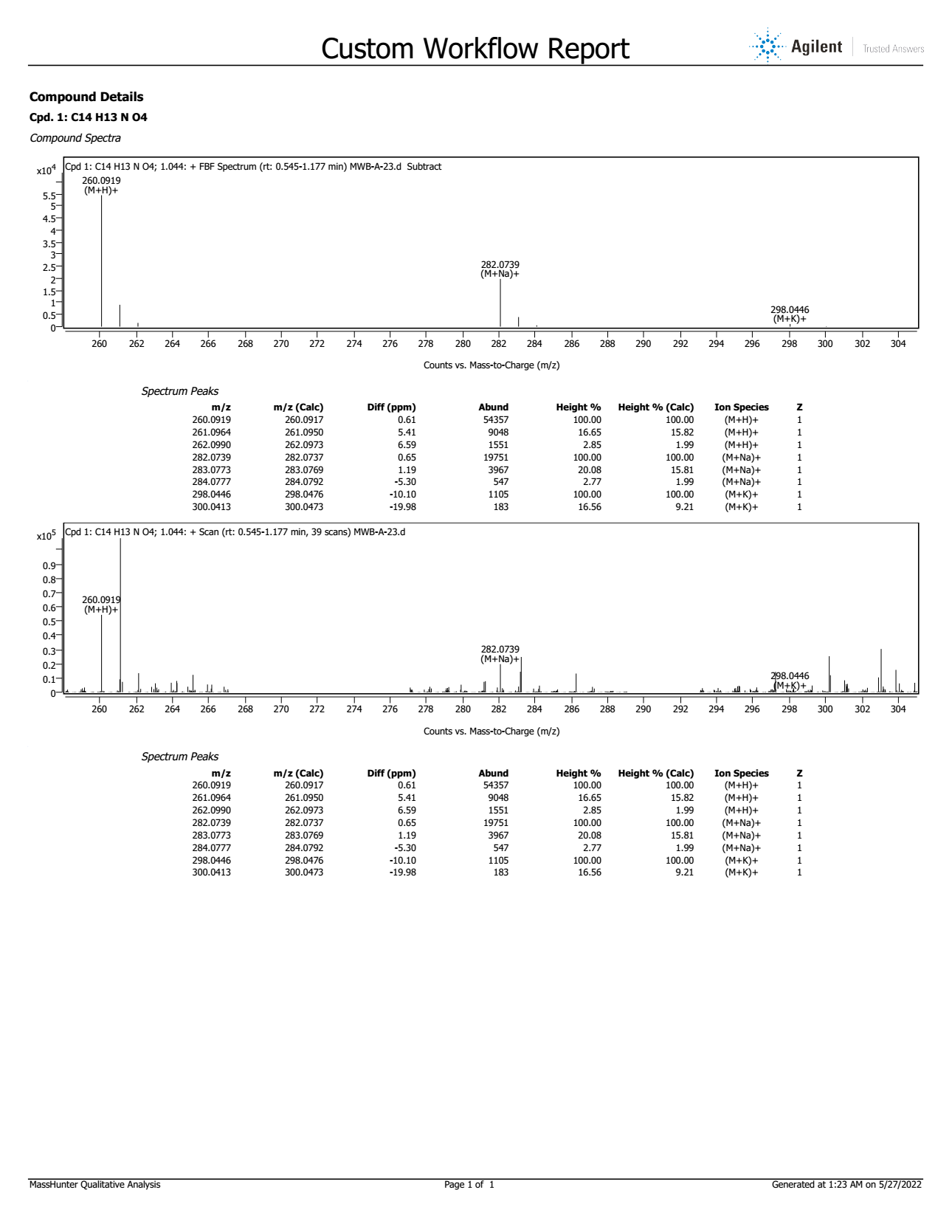
**

^1^H, ^13^C NMR and HRMS Spectra of Compound **3y**

**
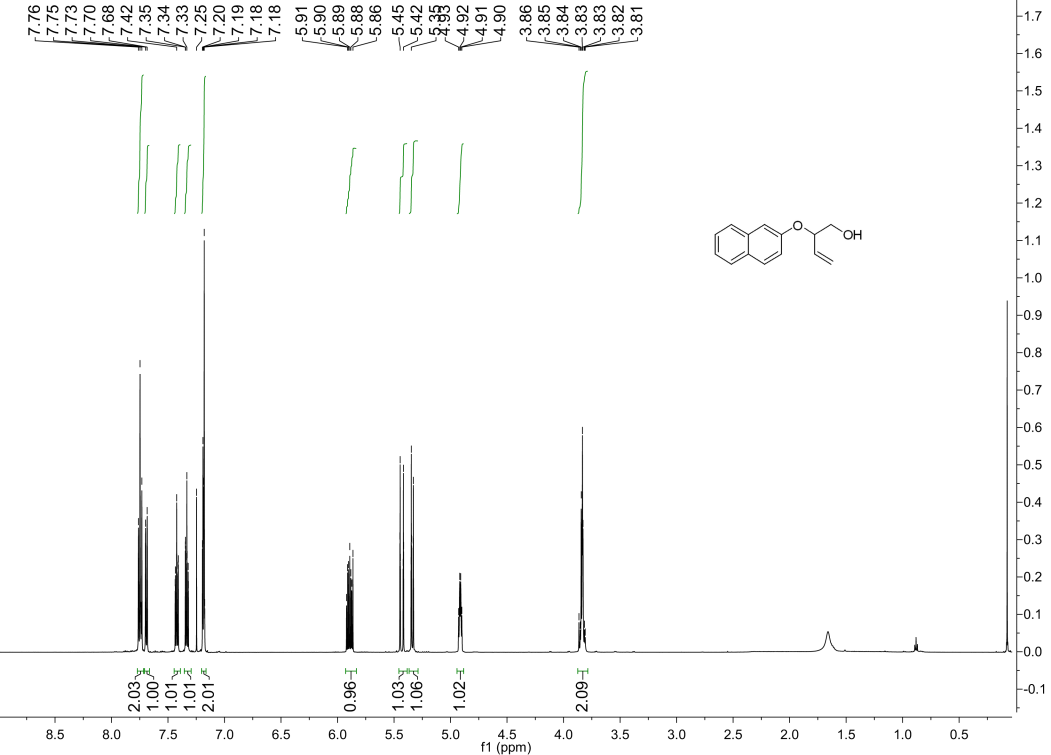
**

**
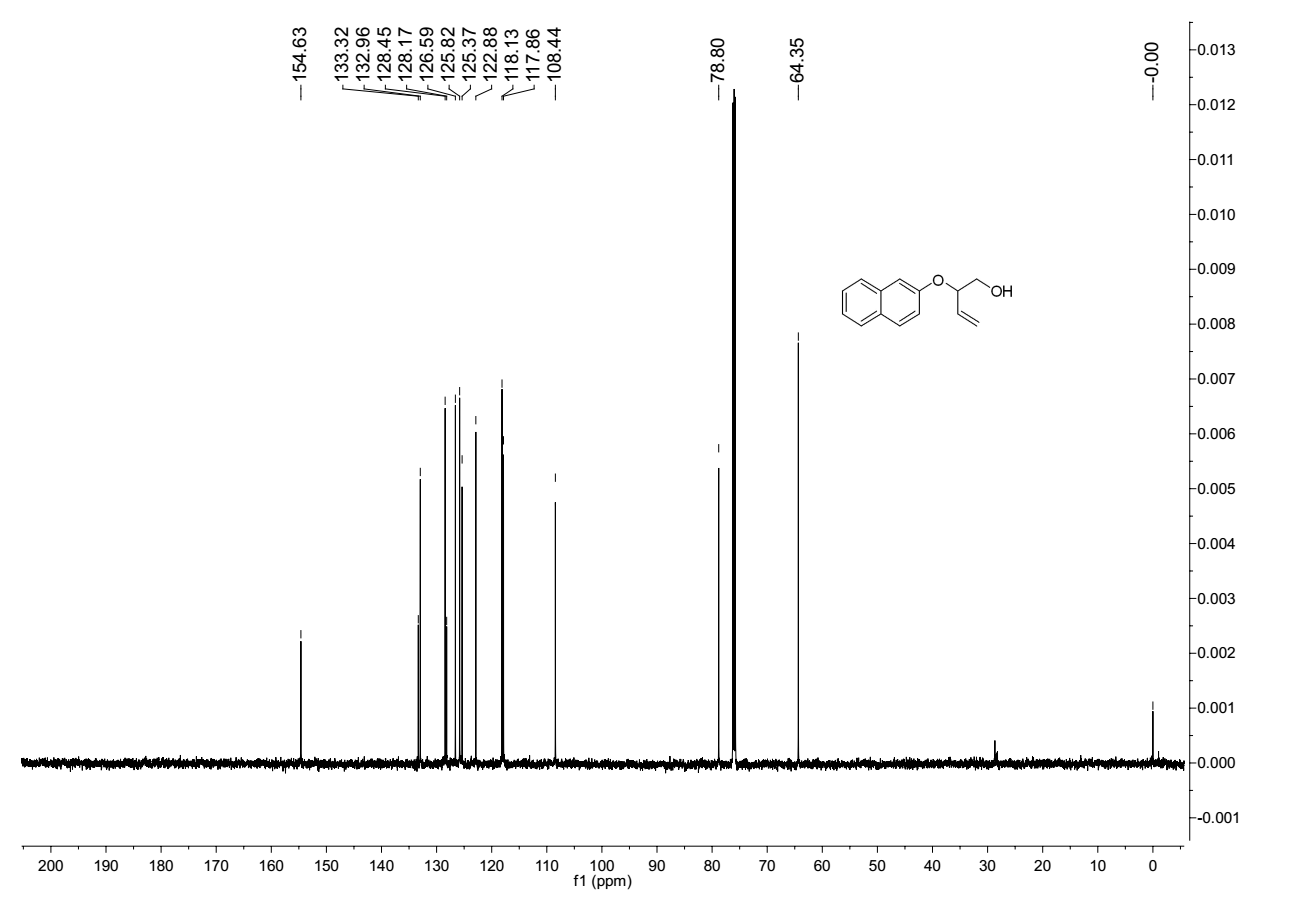
**

**
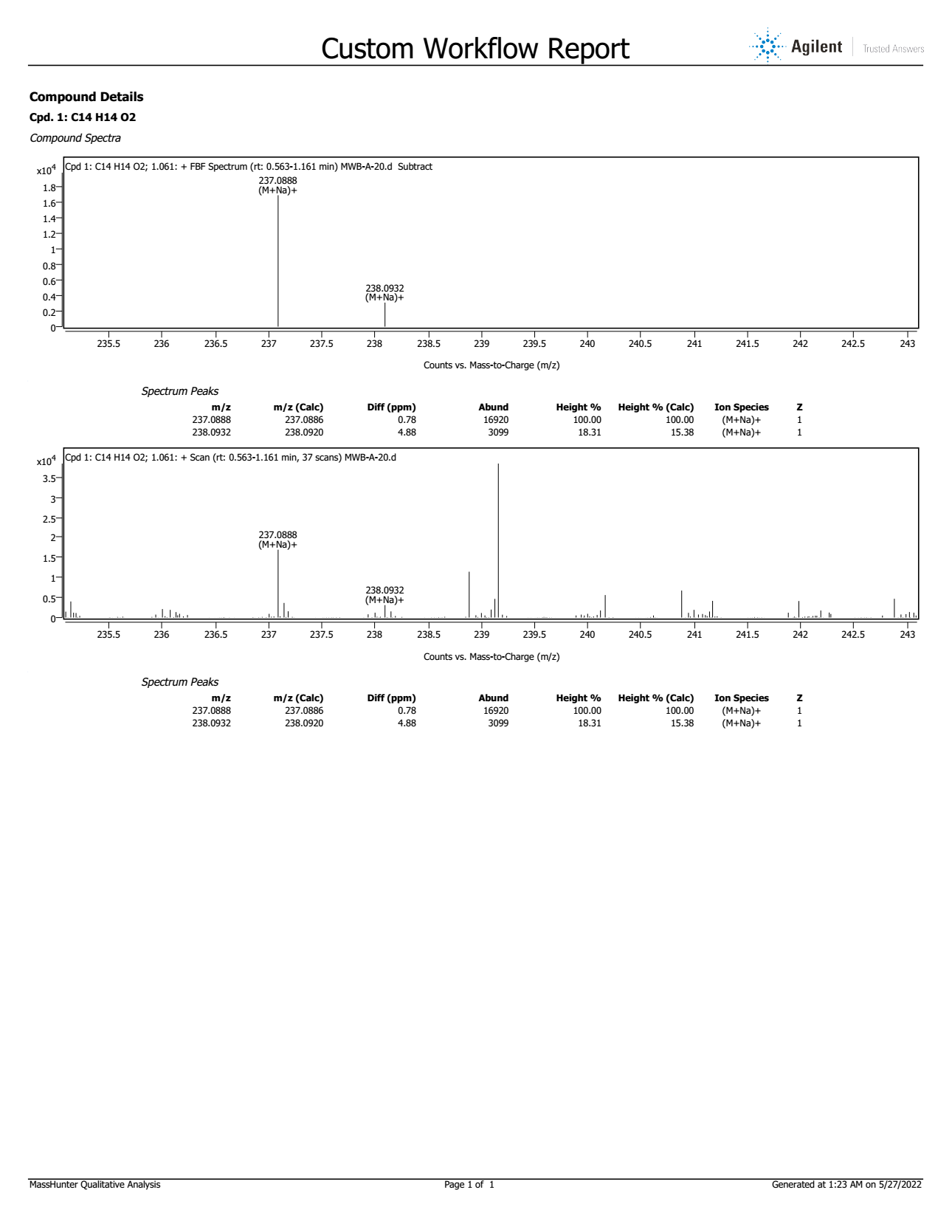
**

^1^H, ^13^C NMR and HRMS Spectra of Compound **3z**


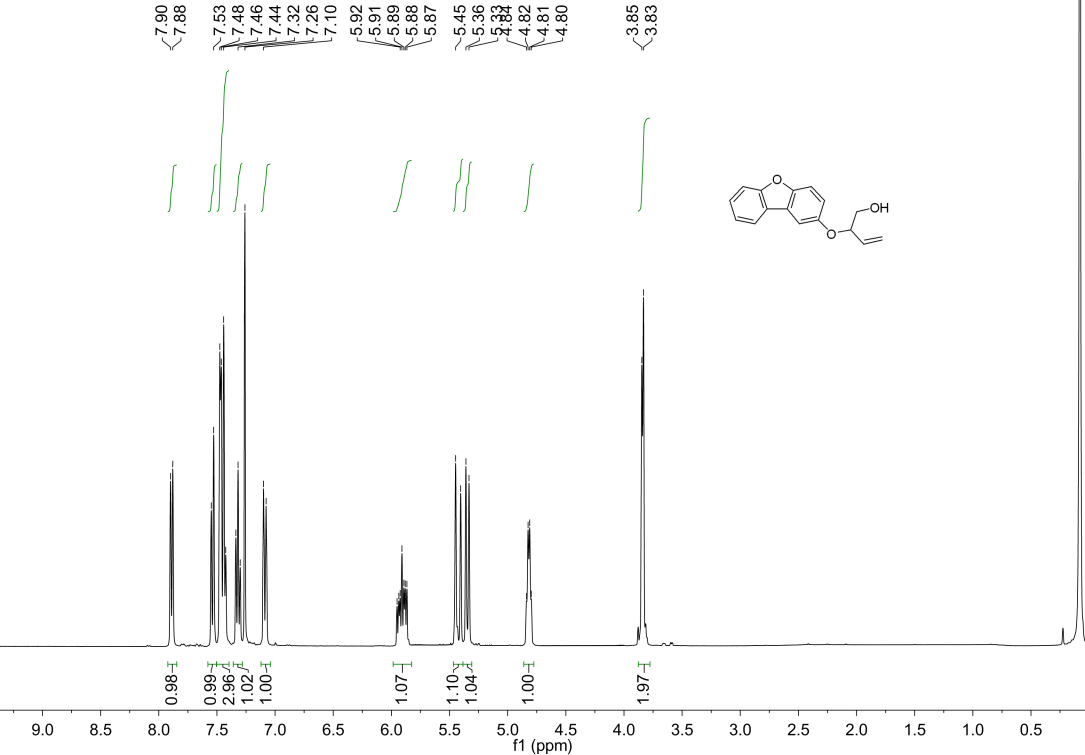


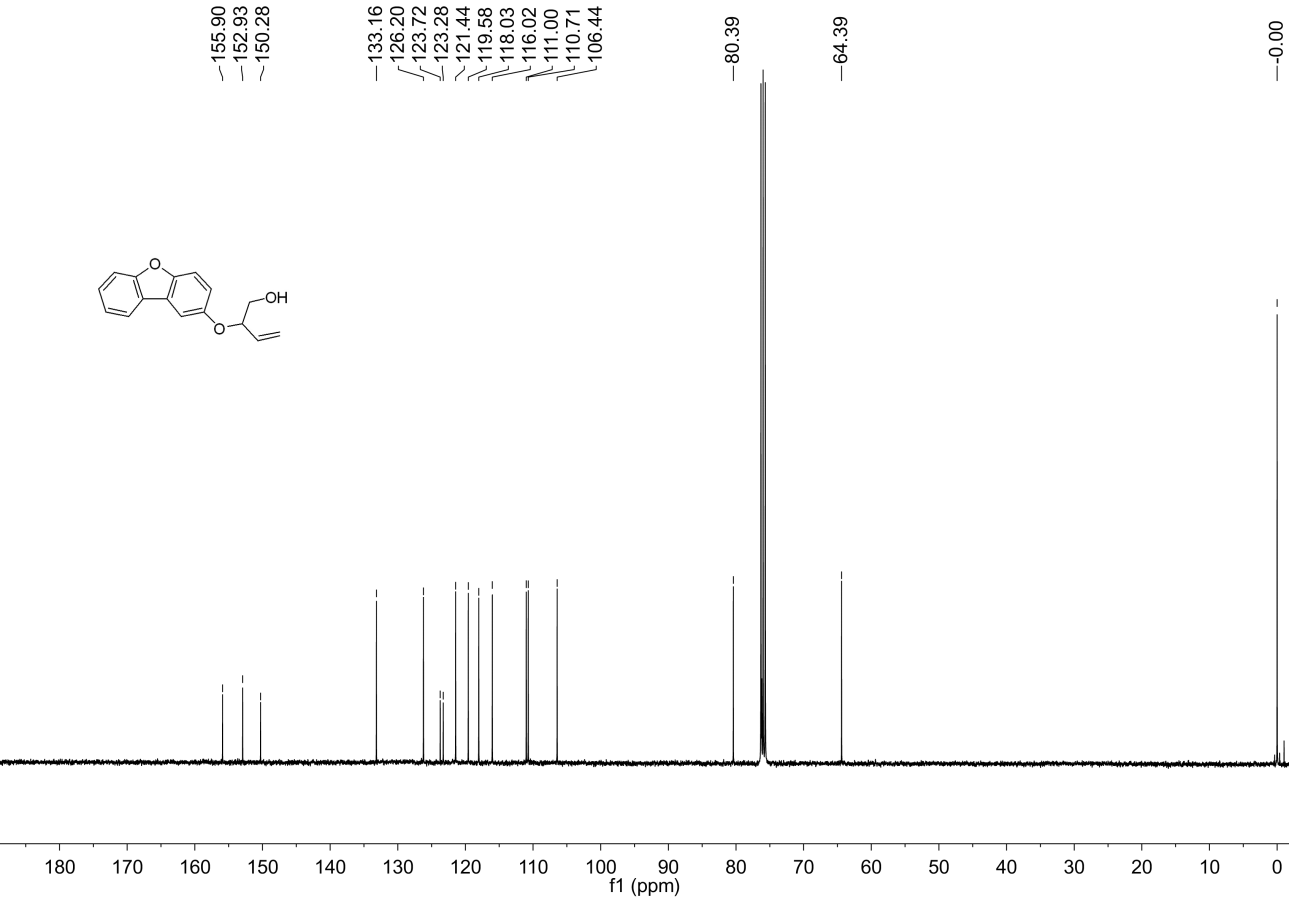


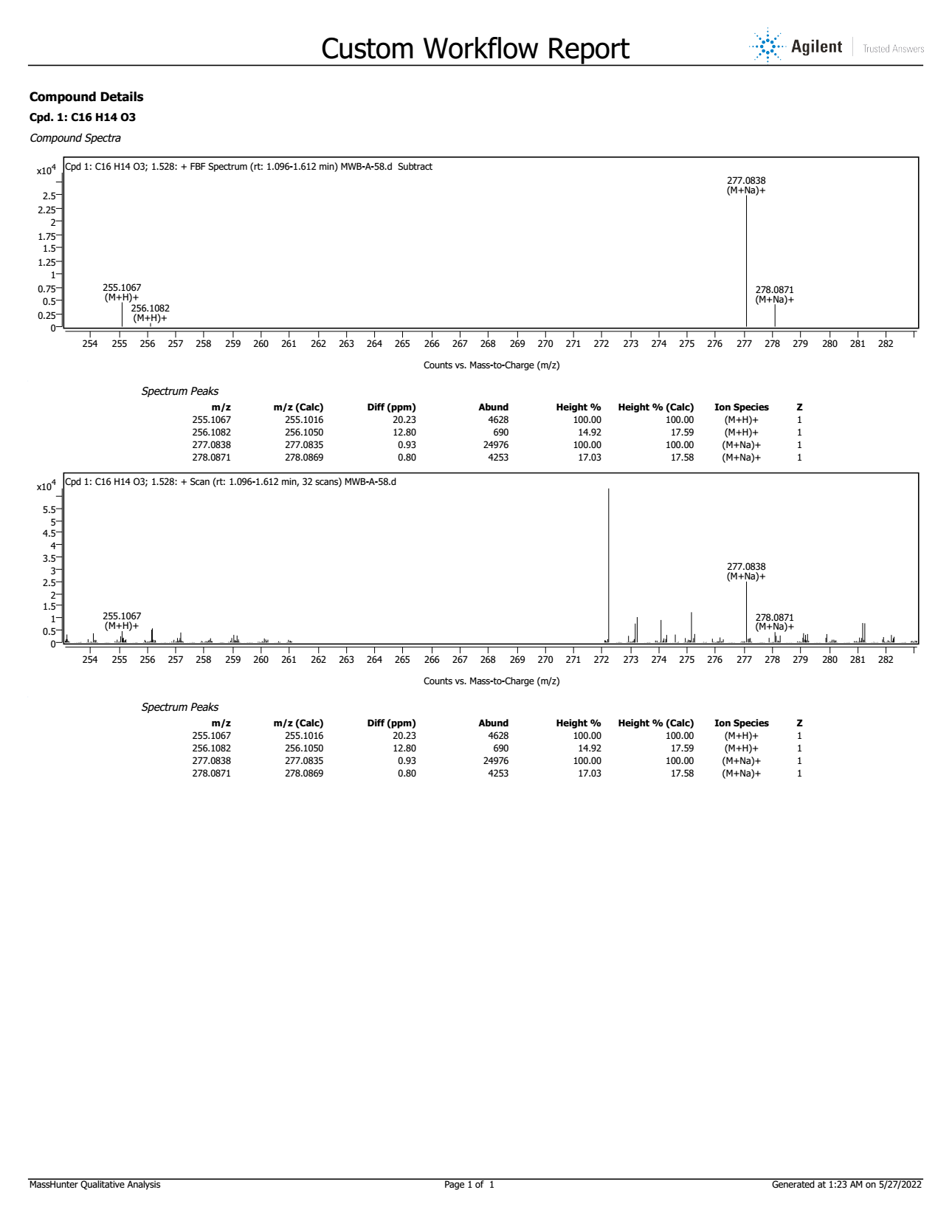


^1^H, ^13^C NMR and HRMS Spectra of Compound **3ab**


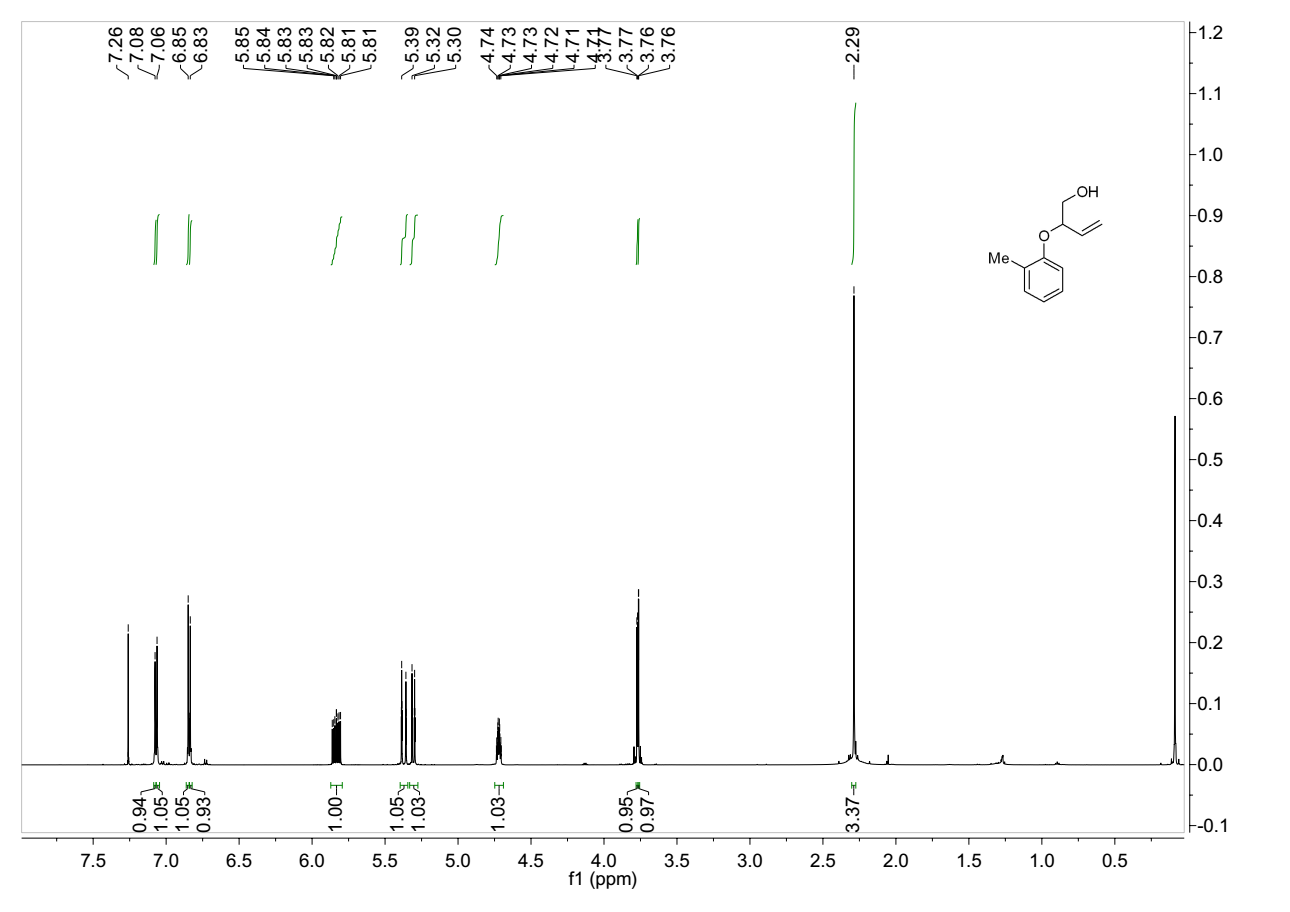


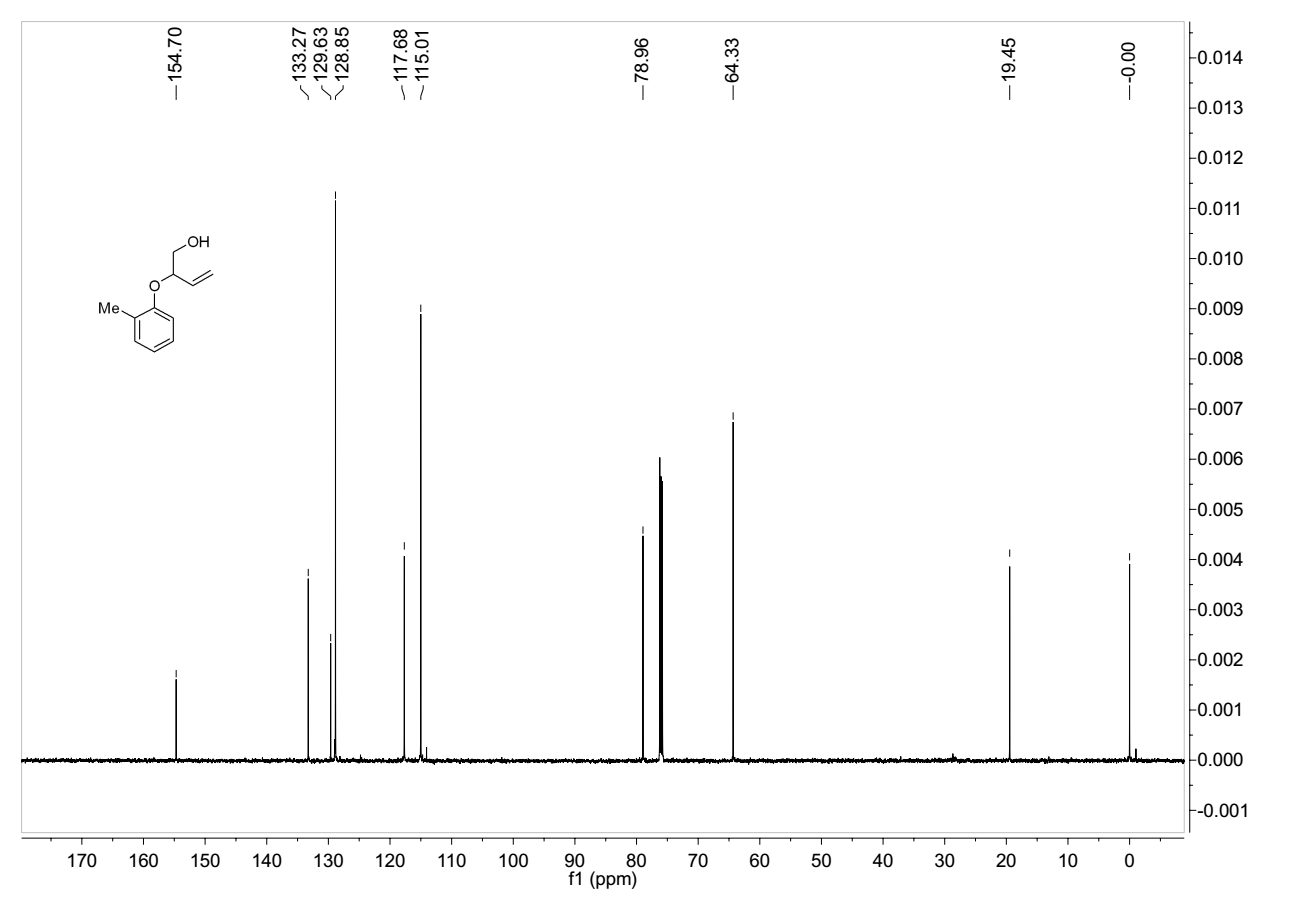


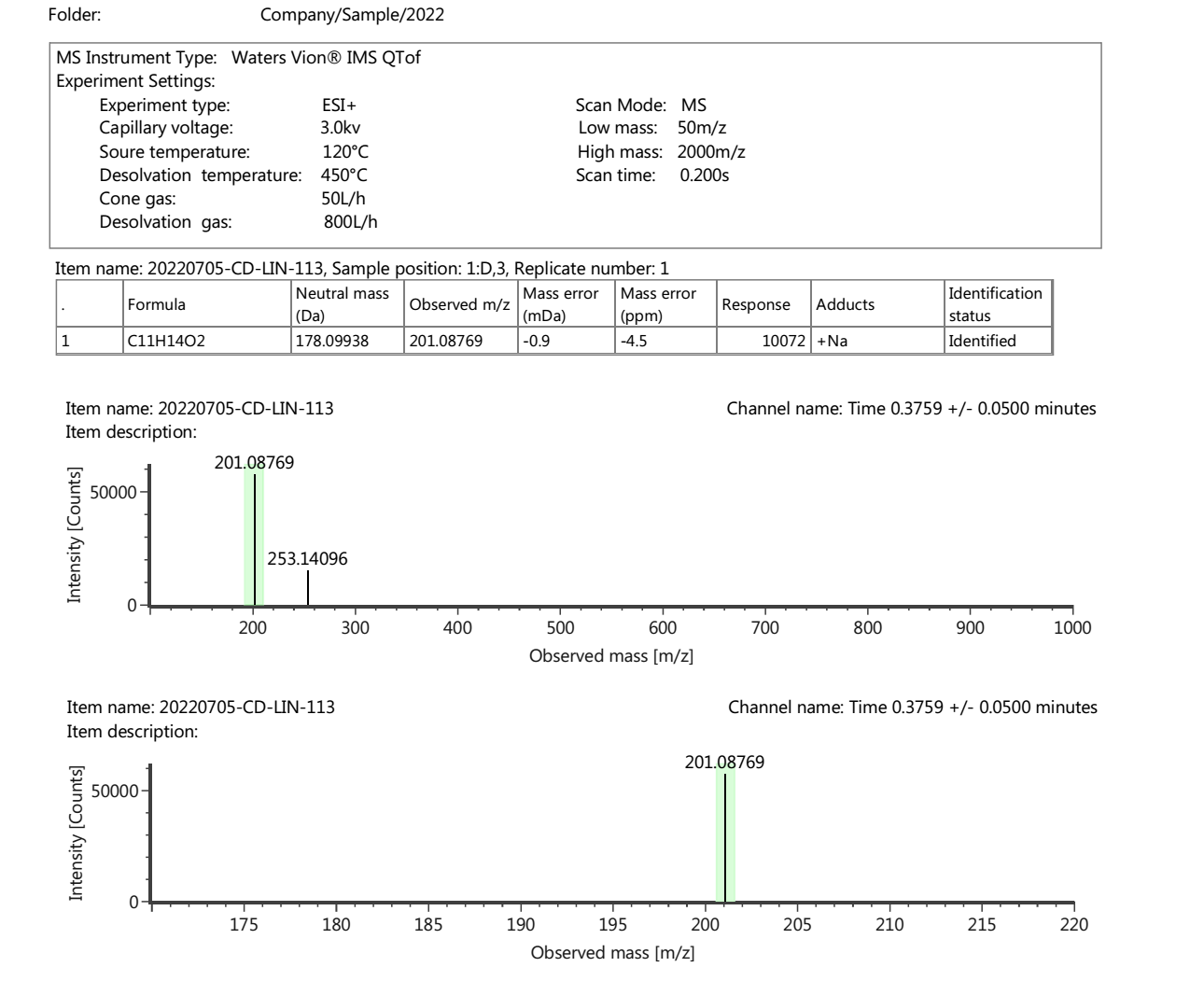

Supplement: Supplementary file 1 [file DataSheet1.docx]
